# Dendritically localized RNAs are packaged as diversely composed Ribonucleoprotein particles with heterogeneous copy number states

**DOI:** 10.1101/2024.07.13.603387

**Published:** 2024-08-04

**Authors:** Renesa Tarannum, Grace Mun, Fatima Quddos, Sharon A. Swanger, Oswald Steward, Shannon Farris

**Affiliations:** 1 Fralin Biomedical Research Institute at Virginia Tech Carilion, Center for Neurobiology Research, Roanoke, Virginia; 2 Translational Biology, Medicine & Health Graduate Program, Virginia Tech, Blacksburg, Virginia; 3 University of California Irvine, Irvine, California; 4 Department of Biomedical Sciences & Pathobiology, Virginia-Maryland College of Veterinary Medicine, Virginia Tech, Blacksburg, Virginia; 5 Virginia Tech Carilion School of Medicine, Roanoke, Virginia

**Keywords:** mRNA localization, Neuronal RNA granule, RNP composition, Multiplex imaging

## Abstract

Localization of mRNAs to dendrites is a fundamental mechanism by which neurons achieve spatiotemporal control of gene expression. Translationally repressed neuronal mRNA transport granules, also referred to as ribonuclear proteins (RNPs), have been shown to be trafficked as single or low copy number RNPs and as larger complexes with multiple copies and/or species of mRNAs. However, there is little evidence of either population in intact neuronal circuits. Using single molecule fluorescence in situ hybridization studies in the dendrites of adult rat and mouse hippocampus, we provide evidence that supports the existence of multi-transcript RNPs with the constituents varying in amounts for each RNA species. By competing-off fluorescently labeled probe with serial increases of unlabeled probe, we detected stepwise decreases in *Arc* RNP number *and* fluorescence intensity, suggesting *Arc* RNAs localize to dendrites in both low- and multiple-copy number RNPs. When probing for multiple mRNAs, we find that localized RNPs are heterogeneous in size and colocalization patterns that vary per RNA. Further, localized RNAs that are targeted by the same trans-acting element (FMRP) display greater levels of colocalization compared to an RNA not targeted by FMRP. Simultaneous visualization and assessment of colocalization using highly multiplexed imaging of a dozen mRNA species targeted by FMRP demonstrates that dendritic RNAs are mostly trafficked as heteromeric cargoes of multiple types of RNAs (at least one or more RNAs). Moreover, the composition of these RNA cargoes correlates with the abundance of the transcripts even after accounting for expression. Collectively, these results suggest that dendritic RNPs are packaged as heterogeneous co-assemblies of different mRNAs and that RNP contents may be driven, at least partially, by highly abundant dendritic RNAs; a model that favors efficiency over fine-tuned control for sustaining long-distance trafficking of thousands of messenger molecules.

## INTRODUCTION

Neuronal morphology is incredibly complex, and in order for neurons to function efficiently RNA transcripts need to be delivered to distant sites for on-demand translation. In particular, RNA localization to synapses and local translation are required for the synaptic plasticity underlying learning (Bradshaw et al., 2003; Huber et al., 2000). Dysregulation of these processes is a common cause of intellectual disability and autism (Fernandopulle et al., 2021; Huber et al., 2002). Thus, uncovering how mRNA cargoes are delivered to and locally regulated at the synapse is central to our understanding of the molecular basis of learning and memory. Furthermore, studying the fundamental principles of neuronal mRNA localization can uncover key aspects of post-transcriptional regulation, which could be applicable to various other organisms and cell types, such as yeasts, drosophila germ granules, cardiomyocytes, etc., as many utilize compartmentalization for gene regulation (Lewis et al., 2018; Martin & Ephrussi, 2009).

Earlier studies prior to the use of modern molecular tools detected only a handful of RNAs localized in neuronal dendrites (Steward & Schuman, 2003). Recent studies using compartment specific deep-sequencing, however, have revealed the presence of thousands of RNA species localized in the hippocampal neuropil (Cajigas et al., 2012; Farris et al., 2019). These localized mRNAs are assembled and transported as RNPs (ribonuclear particles) or granules, which are dynamic, spherical, membraneless macromolecular complexes of translationally inert RNAs, RNA-binding proteins (RBPs) and translational machinery (Kiebler & Bassell, 2006; Knowles et al., 1996). Neuronal RNPs have been shown to be actively transported by conventional kinesins for targeted delivery to subcellular destinations (Köhrmann et al., 1999). Purified RNA granules from brain lysates or dissociated cultured neurons are composed of many different RNAs (Fritzsche et al., 2013; Heraud-Farlow et al., 2013; Kanai et al., 2004) and RBPs (Fritzsche et al., 2013). These biochemically isolated endogenous RNA granules have overlapping RBP constituents and cargo mRNAs, as well as molecular interactors, proteins and mRNAs that are exclusive to specific types of granules. However, visualization of these granules in intact neural circuits have been technically challenging. Thus, there is limited evidence on the heterogeneity and diversity in composition of these granules/ RNPs as well as their spatial distribution in intact neural circuits.

Several different models have been proposed to explain how RNAs are assembled into RNPs/ granules to dictate their destination. One hypothesis is that RNAs are transported as single or low copy number molecules per RNP. This model was supported by experiments performed using single-molecule FISH in cultured neurons which revealed that individual dendritic RNPs, whether in transport or localized, might carry no more than one or two molecules of a specific type of transcript based on few instances of colocalization between different mRNA species (Batish et al., 2012; Park et al., 2014). Our published work visualizing two dendritically localized mRNA species, *Arc* and *Camk2a*, in the adult hippocampus also indicated relatively minimal colocalization, consistent with low mRNA copy number/ contents in transport RNPs (Farris et al., 2014).

However, this model is challenged by recent studies that have identified thousands of RNA species in distant neuronal compartments (Ainsley et al., 2014; Cajigas et al., 2012; Farris et al., 2019). Selective delivery of low copy number RNPs seems at odds with sustaining the localization of thousands of diverse mRNAs with varying abundance and encoded protein functions. Immunoprecipitation studies from brain lysates suggest that dendritically localized mRNAs associate selectively within distinct RNA granules that contain many different types of transcripts (some RNAs overlap, and some are exclusive to each type of granule). Although this model seems plausible and efficient for localizing vast amounts of RNAs, these studies are technically limited by the lack of spatial resolution and non-specific RNA interactions. Addressing this question requires subcellular-resolution imaging of many endogenous molecules at once, which is now technically feasible using highly multiplexed, single molecule fluorescence in situ hybridization with iterative imaging (HiPlex smFISH).

In this paper, we used HiPlex to characterize the compositions of neuronal transport RNPs in mouse hippocampal neuropil. Hippocampus, a brain region critical for learning and memory, has a laminar architecture that affords the opportunity to catalog localized RNAs and visualize their subcellular distribution, in detail, in the dendrite-enriched neuropil layer. Here we show that individual RNPs are likely composed of both single and multiple copies of individual RNAs, consistent with low and high copy number RNP populations. We also show that dendritic RNAs encoding proteins in the same signaling pathway exhibit preferential colocalization in the same RNP when targeted by the same RBP. Lastly, visualization of a dozen localized mRNAs that are targeted by the same RBP revealed that the vast majority of dendritic RNAs are localized as multimeric RNP complexes that have at least two or more different RNA species co-packaged with a relationship between RNA abundance and colocalization. Collectively, these data provide evidence that dendritic RNAs localize into distinct subpopulations with varying RNA contents and colocalization patterns that scale by abundance, thus adding new insights into the diversity and heterogeneity of neuronal transport RNP compositions.

## RESULTS

### *Arc* RNPs contain multiple copies of *Arc* transcripts

**Fig 1:**
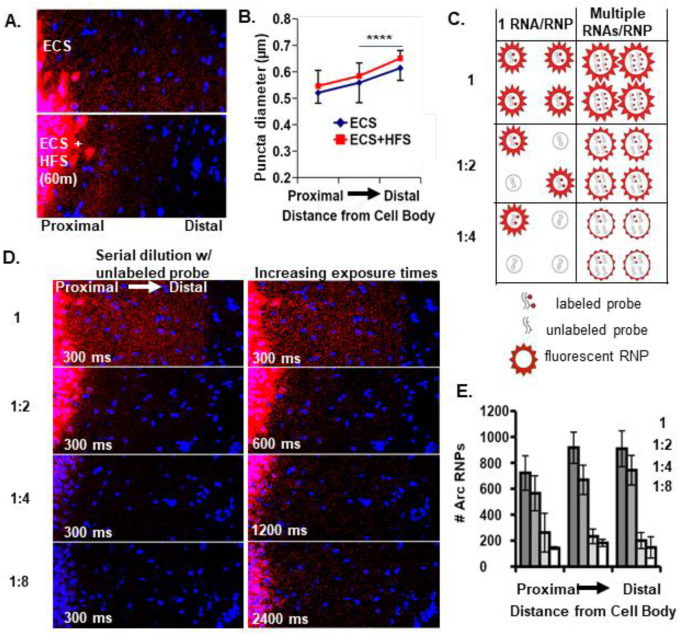
*Arc* RNP number, intensity, and size reflect multiple pools of RNPs. **A.** Representative images of *Arc* mRNA localization to the middle molecular layer of the rat dentate gyrus following ECS and 60 min unilateral HFS. **B.** Quantification of the size of localized *Arc* mRNA puncta (HFS condition) vs. non-localized puncta (ECS) across the molecular layer (middle molecular layers of HFS vs ECS, p>0.05; HFS or ECS middle molecular layer vs outer molecular layer, p<0.0001, KS test). **C.** Possible outcomes of serial probe dilution on single and multiple copy number *Arc* RNPs in terms of number and size of fluorescent RNPs. **D.** Representative images after ECS only labeled with 1X undiluted *Arc* probe or serially diluted with unlabeled *Arc* probe and imaged with identical exposure times, 300 ms,(Left). Images acquired with doubling exposure times (600, 1200, 2400 ms) revealed undetected RNPs at 300 ms (Right), indicating a decrease in RNP intensity as would be expected with multiple RNAs per RNP. **E.** Quantification of *Arc* RNP number for each dilution at 300 ms. A stepwise decrease in *Arc* RNP number suggests single RNA RNPs.

To begin to address whether neuronal RNAs are trafficked into dendrites singly and/or in multiple copy number RNPs, we investigated the RNP properties of the well-known dendritically localized mRNA *Arc* (activity-regulated cytoskeleton-associated mRNA). *Arc* mRNA expression in dentate granule (DG) cell dendrites is unique in that it is tightly regulated by activity-dependent transcription and degradation (Farris et al 2014). At baseline, dendritic *Arc* expression is low or absent in most dentate granules cells, but after a single electroconvulsive shock (ECS) *Arc* mRNA is rapidly transcribed (within ~3 minutes) and transported throughout the DG dendritic laminae by 30 min to 1 hour (Steward et al 1998). Given the short half life of *Arc* mRNA (~45 min (Rao et al., 2006), the prolonged presence of *Arc* mRNA in DG dendrites (e.g. at 2 hrs) is maintained by ongoing transcription and dendritic transport (Farris et al., 2014). Subsequent unilateral high frequency stimulation (HFS) of the entorhinal cortical perforant path inputs to the DG further boosts *Arc* transcription and leads to the accumulation of newly transcribed *Arc* RNA selectively near the activated synapses in the middle molecular layer and a depletion of *Arc* mRNA from the outer molecular layer (Steward et al 1998; Farris et al 2014). Using this stimulation paradigm (ECS + HFS) on adult female rats and fluorescence in situ hybridization (FISH), we assessed the size and number of dendritically-localized (ECS) and synaptically-localized (HFS) *Arc* mRNA puncta as putative RNPs (Fig. 1). We found that *Arc* RNPs are generally of similar size, as measured by feret’s diameter, whether they are localized to dendrites or targeted to recently activated synapses (Fig 1AB, middle molecular layers of ECS vs HFS, p>0.05 KS test). RNPs are larger in distal vs proximal dendrites under both conditions (ECS and ECS+HFS, p<0.0001, KS test). These data suggest that resident and newly transcribed dendritic *Arc* RNPs contain a similar amount of *Arc* RNA per RNP.

Next, in order to assess the transcript occupancy of *Arc* RNPs, we measured *Arc* RNP size and number in rats that received an ECS only (dendritically localized) after serial dilution of 1X labeled *Arc* probe with unlabeled (cold) *Arc* probe (1:2, 1:4, 1:8). We reasoned that a stepwise decrease in *Arc* RNP number would reflect singly transported RNAs that were no longer detectable when half, or fourth or eighth of the probe was unlabeled (Fig 1C). Alternatively, a decrease in apparent size of *Arc* RNPs would reflect a reduction in the number of labeled transcripts from multiple copy *Arc* RNPs (Fig 1C). When acquiring images using identical acquisition parameters (optimized for 1X labeled probe), we detected a stepwise decrease in *Arc* RNP number, with the largest drop off between one half and one fourth cold probe dilution (Fig 1DE). These data are consistent with a population of low copy number (2–4) *Arc* RNPs. However, when we doubled the exposure time after each dilution, we qualitatively saw an increase in the number of *Arc* RNPs indicating that a proportion of the *Arc* RNPs labeled with cold probe dropped below the detection threshold (Fig 1D), consistent with a population of *Arc* RNPs containing multiple copies of *Arc* transcripts. Collectively, the data suggest that there are multiple populations of *Arc* RNPs, those with low and high *Arc* copy numbers that exist in DG dendrites. Given that we did not detect any changes in RNP size after HFS, we assume this finding would translate to synaptically targeted *Arc* RNPs.

### smFISH probes can detect RNA colocalization

**Fig 2.**
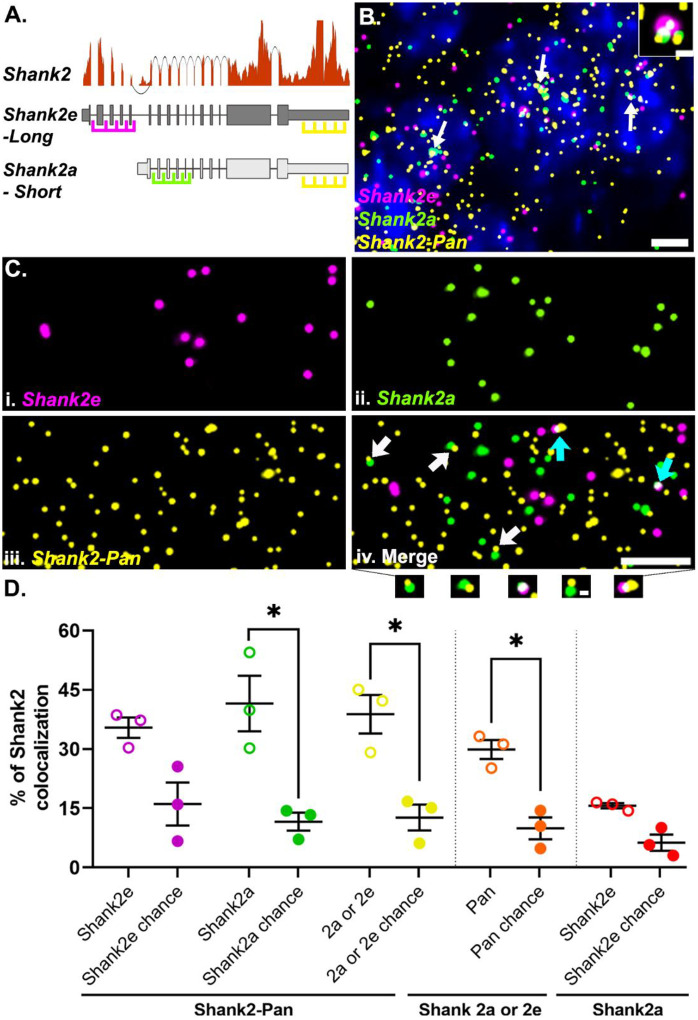
Shank2 isoform-specific 5’ probes are highly colocalized with the Pan 3’ probe. **A.** Shank2 isoform gene models with RNAseq read depth data showing the relative expression levels in hippocampal CA2. Sequences from either long (Shank2e) or short (Shank2a) transcripts targeted by different 5’ probes (magenta and green, respectively) and both targeted by the Pan 3’ probe (yellow) are shown. **B.** Representative image of the three Shank2 probes in CA2 cell bodies. Nuclei are labeled with DAPI (blue). White arrows indicate example transcriptional foci. Dashed white box is the inset showing a transcriptional focus labeled by all three probes. **C.** High-magnification images of (i) Shank2e, (ii) Shank2a, (iii) Shank2-Pan and (iv) the merged image. Arrows indicate colocalization of Shank2-Pan 3’ probe with either Shank2e 5’ probe (cyan arrows) or Shank2a 5’ probe (white arrows) as shown below. **D.** Quantification of the percent colocalization between the Shank2e (magenta) and Shank2a (green) or both (yellow) with the Shank2-Pan probe (open circles) compared to that expected by chance (closed circles). The percent of Shank2-Pan colocalized with either Shank2a or Shank2e (orange) compared to chance and the percent of Shank2e colocalizing with Shank2a (red) compared to chance, many of which are transcription foci, as shown in B. Error bars indicate SEM; N=3 mice; * denotes p<0.05 from paired one-tailed t-test.Scale bars: B) 5μm, 1μm, C) 5μm, 0.5μm

In addition to dendritic RNPs consisting of low or multiple copies of the same RNA (homomeric), we wanted to test whether dendritic RNPs are composed of multiple species of RNA (heteromeric) as described *in situ* for established RNA granules like germ plasm granules (Trcek et al., 2015) or p-bodies (Ford et al., 2019; Teixeira et al., 2005) and identified biochemically for neuronal transport granules (Elvira et al., 2006; Fatimy et al., 2016; Fritzsche et al., 2013; Heraud-Farlow et al., 2013). In order to assess the colocalization of different RNA transcripts, we first needed to confirm that we can reliably detect colocalized smFISH signals. To test this, we took advantage of the fact that there are (at least) two isoforms of *Shank2* expressed in hippocampal area CA2 (Farris et al 2019). The two isoforms are generated via alternative 5’ promoters and thus differ in their 5’ untranslated regions (UTRs), but have identical 3’ UTRs (Jiang & Ehlers, 2013; Monteiro & Feng, 2017). Using isoform specific probes targeted to the two distinct 5’ UTRs (Shank2e-long and Shank2a-short) and a pan Shank2 probe targeted to the common 3’UTR (Shank2-pan, Fig. 2A), we calculated the percentage of colocalized RNPs, defined as puncta in separate channels overlapping by at least 1%. In agreement with RNAseq expression data (Fig 2A), we detected Shank2 expression from all three probes in area CA2 (Fig 2BC). In general, the Shank2-pan probe detected more Shank2 RNPs than the 5’ UTR probes combined (# of RNPs: Shank2-pan = 3056 ± 472, Shank2e = 1122 ± 268, Shank2a = 1463 ± 79.3, N= 3 mice), either due to the (presumed) greater accessibility of the 3’ UTR from less RNA secondary structure compared to the 5’ UTRs, or potentially due to expression of other isoforms that include the 3’UTR but not either of the two 5’UTRs (e.g. Shank2C, Monteiro & Feng, 2017) that cannot be resolved via short-read sequencing. We found that nearly 40% of Shank2e (35.41 ± 2.57%) or Shank2a (41.53 ± 7.05%) colocalizes with the Shank2-pan probe (Fig. 2D). The Shank2-pan probe also colocalizes with either 5’ UTR probe at ~30% (29.88 ± 2.42%). The fact that this relative percentage is not greater than the colocalization of the individual 5’ UTR colocalization is due to both the greater number of Shank2-pan RNPs (described above) and instances where all three probes colocalize at presumed transcriptional foci (Fig 2B, inset). These data are consistent with our previous findings, where ~30% of 5’ and 3’ *Arc* RNA probes colocalize in the dendrites of dentate granule cells in rats (Farris et al., 2014). To account for the amount of colocalization that occurs by chance and is influenced by differences in individual transcript expression levels, we remeasured colocalization for each probe pair after rotating one of the channels 180 degrees. In most cases, we detected a significantly greater percent colocalization than is expected by chance (Fig. 2D). In the instances where percent colocalization is near chance, such as with the two 5’ probes, we assume this to indicate that these two transcripts do not colocalize often outside of transcriptional foci. In summary, our method is able to reliably detect colocalization of two probes targeted to the same RNA, which is a higher bar than for detecting two RNAs within the same RNP, which we assess below.

### Dendritic RNAs encoding G protein signaling proteins show multimodal size distributions and different patterns of colocalization

**Fig 3.**
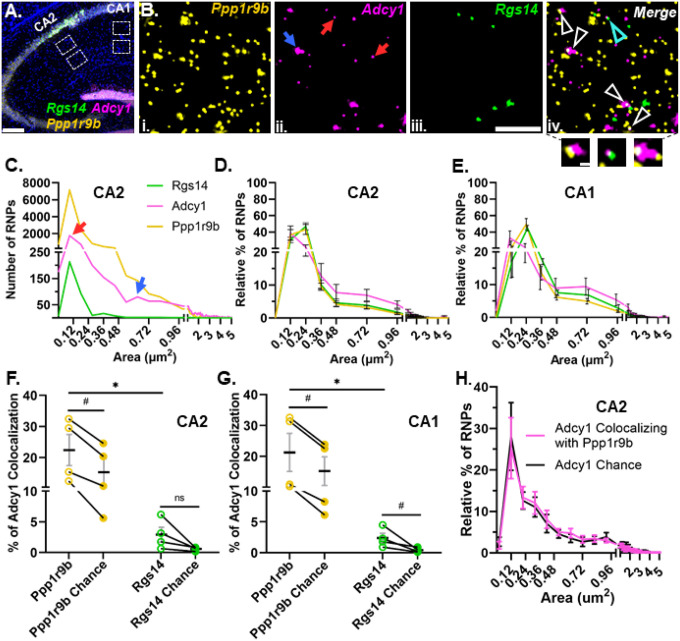
G protein signaling dendritic RNAs show heterogeneity in size distribution and colocalization patterns. **A.** Representative image of *Rgs14* (green), *Adcy1*(magenta) and *Ppp1r9b* (yellow) mRNA and nuclei (in blue) in adult mouse hippocampus. Dashed white boxes are regions analyzed from CA1 and CA2 proximal and distal dendrites. **B.** High-magnification representative images of (i) *Ppp1r9b*, (ii) *Adcy1*, (iii) *Rgs14* and (iv) merged in CA2 distal dendrites. Arrows in red (small sized puncta) and blue (large sized puncta) indicate two distinct populations of *Adcy1* mRNA based on the size of the fluorescent puncta. (iv) Arrows indicate colocalization of *Adcy1* RNP with either *Ppp1r9b* RNP (white) or *Rgs14* RNP (cyan). Three example images of RNP colocalization in the callout section. **C.** Histogram from a representative mouse showing the size distributions of *Rgs14*, *Adcy1* and *Ppp1r9b* RNPs measured as the size of fluorescent puncta in CA2 distal dendrites. Red and blue arrows denote the two populations of *Adcy1* RNPs based on size. **D & E.** Relative percent histograms showing the average size distributions of *Rgs14*, *Adcy1* and *Ppp1r9b* from N=4 mice in CA2 and CA1 dendrites. Error bars indicate SEM. **F & G.** Quantification of the percent of *Adcy1* RNPs colocalizing with *Ppp1r9b* (yellow, open circles) and *Rgs14* (green, open circles) in CA2 (*Adcy1*/*Ppp1r9b* = 22.47 ± 4.99%; *Adcy1*/*Rgs14* = 2.93 ± 1.2%, p-value= 0.019, paired one-tailed t-test) and CA1 dendrites (*Adcy1*/*Ppp1r9b* = 21.24 ± 6.17%; *Adcy1*/*Rgs14* = 2.378 ± 0.76%, p-value= 0.023, paired one-tailed t-test). The percent colocalization expected due to chance was quantified by rotating the *Adcy1* image 90 degrees and calculating the percentage of puncta overlapping with *Ppp1r9b* (yellow, closed circle) or *Rgs14* mRNAs (green, closed circles). The percentages of *Adcy1* RNPs colocalizing with *Ppp1r9b* or *Rgs14* were significantly greater than what would be expected by chance (*Ppp1r9b* CA2: 22.47 ± 4.99%, chance 15.37 ± 4.30%, p-value=0.003; *Ppp1r9b* CA1: 21.24 ± 6.17%, chance 15.18 ± 4.64%, p-value= 0.016; *Rgs14* CA2: 2.93 ± 1.2%, chance 0.6±0.15% p-value 0.06; *Rgs14* CA1: 2.378 ± 0.76%, chance 0.41 ± 0.18%, p-value 0.0405, paired one-tailed t-tests) **H.** Size distribution histogram for the subpopulation of *Adcy1* RNPs that colocalize with *Ppp1r9b* shows there is no size bias for colocalization with *Ppp1r9b*. Error bars indicate SEM. Scale bars: A) 200μm, B) 5μm, 0.5μm.

Once we demonstrated that our method can reliably detect colocalization when we expect it, we explored whether known dendritic mRNA transcripts localize singly or in association (colocalized) with each other as heteromeric RNPs. We started with known dendritic mRNAs that encode signaling proteins in the same biological pathway (i.e. G-protein signaling), rationalizing that this might be an efficient way to co-assemble RNAs for transport to a similar destination or signaling hub. We performed smFISH for three known hippocampal dendritic RNAs within the G-protein signaling pathway, *Rgs14* (Farris et al., 2019; Squires et al., 2018), *Adcy1* (Farris et al., 2019; Lein et al., 2007) and *Ppp1r9b* (Hale et al., 2021; Zeitouny et al., 2023). Consistent with previous observations, all three mRNAs are localized throughout the dendritic laminae, in both proximal and distal dendritic regions of the neuropil in CA2 and CA1 cell types of the hippocampus (Fig 3AB). However, each mRNA expressed a distinct dendritic expression pattern, including differences in RNP number and size, that did not differ by cell type. Quantification of the number and pixel size of fluorescent puncta of individual RNPs revealed that their expression varies from hundreds (*Rgs14*) to thousands (*Adcy1* and *Ppp1r9b*) of transcripts (Fig 3C). The distal dendrites exhibit a significantly lower abundance of *Rgs14* RNPs compared to the proximal dendrites in CA2 and CA1 (# of *Rgs14* RNPs: CA2 proximal = 784 ± 145 and distal = 177 ± 66, p-value = 0.026; CA1 proximal = 413 ± 38 and distal = 133 ± 36, p-value = 0.013, N=4 mice, paired one-tailed t-tests), while the abundance of *Adcy1* and *Ppp1r9b* RNPs remains relatively equivalent across layers of both cell types (# of *Adcy1* RNPs: CA2 proximal = 2993 ± 939 and distal dendrites = 2683 ± 307, p-value= 0.193; CA1 proximal = 2138 ± 798 and distal dendrites = 2500 ± 782, p-value = 0.27; # of *Ppp1r9b* RNPs: CA2 proximal = 8133 ± 1875 and distal = 9502 ± 2208 p-value= 0.061; CA1 proximal = 6631 ± 1923 and distal= 7279 ± 1955, p-value = 0.178, paired one-tailed t-tests). We quantified the size distributions of these mRNAs in CA1 and CA2 proximal and distal dendrites and found no detectable differences between proximal and distal dendrites and therefore the data were collapsed and represented as one population per animal. Across animals, we consistently observed the same pattern of size heterogeneity across mRNAs in both hippocampal cell types (Fig 3DE). However, the distributions of RNP sizes were quite heterogeneous across RNAs; in particular, *Adcy1* displayed (at least) two qualitatively distinct populations of RNP sizes; those measuring less than 0.25 μm^2^ and a population measuring greater than 0.5 μm^2^ (Fig. 3C indicated with red and blue arrows, respectively). Notably, the larger sized *Adcy1* RNP population makes up over 20% of total *Adcy1* RNPs, whereas it makes up less than 10% of total *Ppp1r9b* RNPs and large RNPs are not present for *Rgs14* (Fig. 3DE). When we compared the size distributions, we found that *Adcy1* is significantly different from that of both *Rgs14* and *Ppp1r9b* regardless of the cell type (p<0.0001 for all comparisons, two-tailed unpaired Kolmogorov-Smirnov tests, N=4 mice). We interpret the larger sized RNPs to potentially represent RNPs with multiple copies of the same transcript whereas the smaller RNPs may represent RNPs containing fewer copies of transcripts or a single copy.

Subcellular localization of a given RNA is assumed to be affected by the composition of the transport RNP (Mikl et al., 2011; Mitsumori et al., 2017). Thus, we tested whether variable RNP size distribution patterns could reflect distinct RNP compositions that have different combinations and/or amounts of RNAs. In both cell types, *Adcy1* mRNA colocalized with *Ppp1r9b* significantly more than it did with *Rgs14* (*Ppp1r9b* CA2: 22.47 ± 4.99% vs *Rgs14* CA2: 2.93± 1.20%, p = 0.019; *Ppp1r9b* CA1: 21.24 ± 6.17% vs *Rgs14* CA1: 2.38 ± 0.76%, p=0.023, N=4 mice, paired one-tailed t-tests) (Fig 3FG). However, the number of *Ppp1r9b* RNPs is almost tenfold higher compared to the number of *Rgs14* RNPs in the hippocampal neuropil. To account for the potential bias of expression differences influencing the extent of colocalization, we rotated the *Adcy1* image 90 degrees and re-calculated the overlapping pixels by chance with either *Ppp1r9b* or *Rgs14*. We found that the chance levels of overlap between *Adcy1* and *Ppp1r9b* were significantly lower than the levels of overlap calculated from the properly registered image in both cell types (Chance CA2: 15.37 ± 4.30, p = 0.003; Chance CA1: 15.18 ± 4.64%, p= 0.016, paired one-tailed t-test, Fig 3FG). Similar results were found for percent colocalization of *Adcy1* with *Rgs14* in that colocalization values were above chance (Chance CA2: 0.6 ± 0.15% p= 0.06; Chance CA1: 0.41 ± 0.18% p= 0.0405, paired one-tailed t-tests). Interestingly, both *Adcy1* and *Ppp1r9b* are targeted by the same RNA-binding protein, fragile X messenger ribonucleoprotein (FMRP) but *Rgs14* is not (Darnell et al., 2011; Hale et al., 2021). Thus, one interpretation of our finding is that RNAs that are bound by the same RBP show preferential colocalization compared to RNA pairs not bound by the same RBP. This suggests that there may be a biologically efficient form of coregulation for RNAs harboring the same or similar cis sequence motifs.

To test whether the larger *Adcy1* RNPs reflected a unique pool of RNPs with more or less colocalization, we compared the size of *Adcy1* RNPs that colocalized with *Ppp1r9b* in experimental images vs the size of *Adcy1* RNPs that colocalized by chance (Fig 3H). We found that the size of *Adcy1* RNPs did not influence its’ colocalization with *Ppp1r9b* as demonstrated by the similar size distributions of colocalized *Adcy1* RNPs in experimental vs random images in CA2 (Fig 3H) and CA1 (data not shown). The larger RNPs colocalized slightly more than the smaller RNPs presumably due to consisting of more pixels with more opportunity to colocalize by chance. Indeed, the average median size of colocalized *Adcy1* RNPs did not differ from chance (CA2: 0.36 ± 0.09 μm^2^ vs. chance = 0.36 ± 0.09 μm^2^, p>0.05, unpaired two-tailed t test; CA1: 0.34 ± 0.06 μm^2^ vs. chance 0.32 ± 0.07 μm^2^, p>0.05, unpaired two-tailed t test, N=4 mice). Collectively, these data extend our findings to suggest that, in addition to low and multiple copy homomeric RNPs, there are heteromeric RNPs, potentially targeted by the same RBPs, indicative of multiple potential mechanisms regulating RNP composition in dendrites.

### Putative FMRP-targeted dendritic RNAs show heterogeneous size distributions

**Fig 4.**
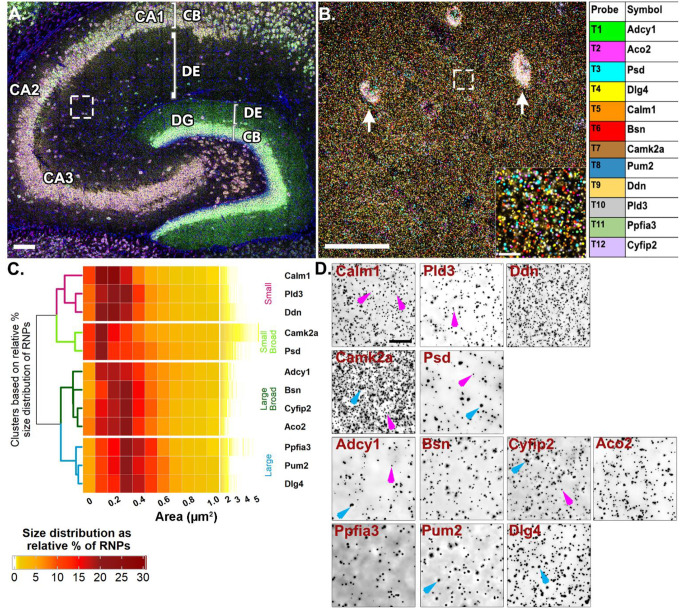
Highly multiplexed RNA imaging reveals dendritic RNAs have variable size distributions **A.** Representative image of mouse hippocampus with *Adcy1, Aco2, Psd, Dlg4* labeling in round 1 of Hiplex smFISH. White box represents ROI from CA2 dendrites. **B.** Representative high-magnification merged image of 12 mRNAs from CA2 dendrites. Arrows denote interneuron expression in the neuropil layer that is removed before analysis (see methods) and inset is the dashed white box. Each mRNA is colored based on the table on the right. **C.** Heatmap of RNP size distributions hierarchically clustered by similarity. **D.** Representative images of each RNA channel. Magenta arrows denote small RNPs in *Calm1*, *Pld3*, *Camk2a*, *Psd*, *Adcy1* and *Cyfip2*. Blue arrows denote larger sized RNPs in *Camk2a*, *Adcy1*, *Cyfip2*, *Ppfia3* and *Dlg4*. Scale = A. 100μm, B. 50μm, 10μm, D. 10μm

Inspired by the observation of homomeric RNA cargoes as well as preferential colocalization between RNAs targeted by the same RBP (heteromeric RNPs), our next experiments were done to 1) extend our findings on dendritic RNP sizes and compositions and 2) to test whether the patterns of colocalization vary when many RNAs are probed at once in intact neural circuit. Particularly, we wanted to test whether RNAs that are more likely to colocalize based on their shared RBP cis motifs show any selectivity in how they associate with other RNAs based on their encoded protein functions or targeted subcellular destination. To test these, we took advantage of the relatively well characterized RBP, FMRP, and its dendritically localized target RNAs to quantitatively map their colocalization at subcellular resolution in hippocampal dendrites. We generated a list of candidate target RNAs by cross-referencing datasets that identified hundreds of putative FMRP target RNAs using HITS-CLIP (high-throughput sequencing of RNA isolated by crosslinking immunoprecipitation) on whole brain (Darnell et al., 2011) and hippocampal CA1 neuropil (Sawicka et al., 2019) with datasets that identified high-confidence hippocampal dendritic RNAs (Farris et al 2019; Cajigas 2012; Ainsley 2014). This list of dendritically localized candidate FMRP target RNAs was further curated based on expression, different encoded protein functions (signaling, cytoskeletal, synaptic plasticity, etc.) and target destinations (mitochondria, cytoplasm, cell membrane, dendritic spine) (Supp. Table 1). To spatially map the association of putative FMRP target RNAs, we probed for 12 RNAs at once and iteratively imaged 4 at a time using Hiplex smFISH followed by FMRP immunostaining (Supp. Fig 1). Each of the mRNAs were present in CA2 dendrites with varying degrees of abundance (Fig 4AB, Supp. Fig 2). Based on the findings from our previous experiments (Fig 1 and Fig 3), which indicated the presence of distinctly sized populations of homomeric RNPs within the same RNA species, we first calculated the median sizes (supplemental table 2) and plotted the size distributions of the 12 putative FMRP target RNAs. Unsupervised hierarchical clustering analysis of the size distributions revealed four different patterns (Fig. 4CD). The first cluster is comprised of RNAs with consistently “small” RNPs (*Calm1*, *Ddn*, and *Pld3* dendrogram labeled in magenta, Fig 4C), whereby, on average, ~55% of RNPs are less than 0.3 μm^2^ (54.57 ± 4.84%, averaged across RNAs from N=4 mice) with the largest relative percent peak (23.69 ± 1.77%) at 0.2 μm^2^. These consistently small RNPs have fewer than 10% RNPs (9.29 ± 1.46%) sized 0.6–1.0 μm^2^ and only 3% (2.50 ± 0.64%) of RNPs larger than 1.0 μm^2^. In contrast, the last cluster is comprised of RNAs with consistently “large” RNPs (*Dlg4*, *Pum2* and *Ppfia3*, labeled in blue, Fig 4C) whereby, on average, ~50% of the RNPs are 0.3–0.5 μm^2^ (49.80 ± 1.16%) with the largest relative peak (20.33 ± 0.42%) at 0.3 μm^2^. These consistently large RNPs have less than 5% RNPs larger than 1.0 μm^2^ (3.14 ± 0.26%). There are two intermediary clusters, “small broad” (*Camk2a* and *Psd*, labeled in light green, Fig 4C) and “large broad” (*Adcy1*, *Bsn*, *Aco2* and *Cyfip2*, labeled in dark green, Fig 4C) that have relatively broader size distributions that segregate with either the “small” or “large clusters”, respectively. The “small broad” cluster (*Camk2a* and *Psd*) shows a very broad distribution with the largest relative peak (21.21 ± 0.72%) equal to or less than 0.2 μm^2^ and a larger population of RNPs sized 0.6–1.0 μm^2^ that accounts for more than 15% (15.89 ± 1.71%). These “small broad” RNAs have the largest fraction of RNPs greater than 1.0 μm^2^ at nearly 10% (9.38 ± 1.34%). The “large broad” cluster (*Adcy1*, *Bsn*, *Aco2* and *Cyfip2*) also shows a broad distribution with the largest relative peak (38.85 ± 1.12%) between 0.2–0.3 μm^2^ and a larger population of RNPs sized 0.6–1.0 μm^2^ that accounts for ~15% (15.31 ± 1.05%). However, these “large broad” RNPs have only ~3% of RNPs larger than 1.0 μm^2^ (2.69 ± 0.60%). Small and large populations are denoted on the representative images with magenta and blue arrows, respectively (Fig. 4D). It is interesting to note that even some of the most abundant dendritic RNAs visualized here contain consistently small populations of RNPs (i.e., *Ddn*, *Calm1*). These data extend our findings from Fig. 3 to show that RNAs, regardless of abundance, vary considerably in RNP sizes, both within a transcript population and across different transcripts. While it is likely that the differently sized RNPs reflect copy number variation, it is not clear whether differences in RNP size reflect functional differences, such as associations with other RNAs and/or their capacity for translation.

### Putative FMRP-targeted dendritic RNAs colocalize based on abundance

**Fig 5.**
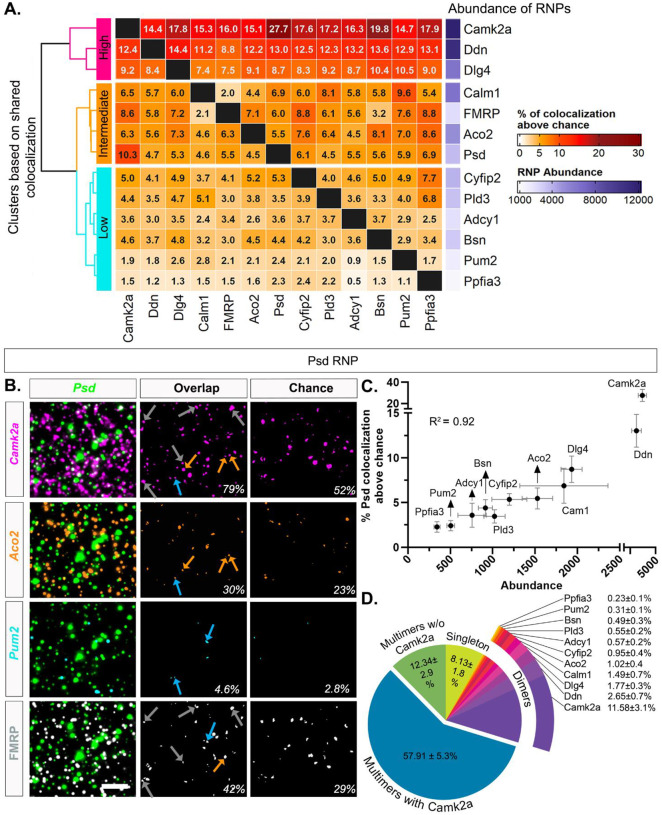
RNP pairwise colocalization patterns correlate with abundance **A.** Percentage of RNPs colocalized with each other above chance in pairwise combinations. Percentage was calculated by dividing the number of overlapping puncta by the total number of RNPs that correspond to each channel. **B.** Representative images of colocalization. The first column consists of merged images of *Psd* (green,) with i) *Camk2a* (magenta), ii) *Aco2* (orange), iii) *Pum2* (cyan) and iv) FMRP (white). The middle column shows the intersecting area of each pair of RNPs *Psd*/ *Camk2a* (magenta,85 out of107 or 79% of *Psd* RNPs colocalize with *Camk2a* in this image), *Psd*/*Aco2* (orange, 33/107, 30%), *Psd*/ *Pum2* (cyan, 5/107, 4.6%) and *Psd*/ FMRP (white,45/107, 42%). The third column shows the intersect of *Psd* by chance (rotated 90 degrees) (*Psd*/ *Camk2a* = 56/107, 52%; *Psd*/ *Aco2* = 25/107, 23%; *Psd*/ *Pum2* = 3/107, 2.8%; *Psd*/ FMRP = 32/107, 29%). Arrows denote examples of *Psd ‘*Multimers with *Camk2a*’ colored by channel. **C.** Correlation plot of percent *Psd* colocalized with other 11 RNPs after chance subtraction and their abundance (R^2^ = 0.92, p<0.0001). **D.** Pie chart of *Psd* RNP compositions. The data shown here is from four 512X512 ROIs per animal then averaged across N=4 mice. 91.86 ± 1.77% (vs. 65.1 ± 5.37% chance in Supp. Fig. 5) of *Psd* RNPs have overlapping pixels from other RNA channels, i.e., colocalized RNAs that include dimers (only one other RNA) or multimers (at least two other RNAs) before chance subtraction. Of the dimers, the percentage of colocalized *Psd* RNPs are almost at the level of chance for every other RNA that *Psd* colocalizes with except *Camk2a*. This is shown by 0.23 ± 0.1% of *Psd-Ppfia3* dimers (vs. chance 0.23 ± 0.1%) compared to 11.58 ± 3.1% of *Psd-Camk2a* dimers (vs. chance 6.68 ± 1.1%) (the highest expressor). 57 ± 5.3% of *Psd* RNPs are multimers that have *Camk2a* and at least one other RNA colocalized which is considerably higher than chance (34.89 ± 4.9%). 12.34 ± 2.9% of *Psd* RNPs are also multimers (vs. chance 8.29 ± 1.6%) but do not have *Camk2a*. Lastly, 8.13 ± 1.8% of *Psd* RNPs are not colocalized with any of the other RNAs in our dataset that is noticeably lower than expected by chance (34.9 ± 5.4%). Scale: B. 5μm

To systematically characterize whether any particular FMRP target RNAs display similar colocalization profiles (and thus suggestive of co-regulation), we measured overlapping pixels across RNA channels to determine pairwise colocalization values, which were corrected for chance colocalization as described above. Pairwise RNA-RNA colocalization percentages were computed by counting the number of overlapping puncta in between two channels of interest and expressed as a percentage separately for each RNA of the pair. The degree of colocalization across pairs in properly registered images ranged from 4.21 ± 0.48% (*Ppfia3*/*Adcy1*) to 71.38 ± 4.91% (*Camk2a*/*Ppfia3*) (Supp. Fig 3A). The degree of colocalization that would be expected by chance (one image from every pair rotated 90 degrees, which controls for the expression of both RNAs in the pair) ranged from 3.07 ± 0.41% (*Aco2*/*Ppfia3*) to 53.74 ± 4.75% (*Pum2*/*Camk2a*) (Supp. Fig 3B). We then subtracted the chance colocalization percentage from the percentage obtained from the properly registered image and visualized the result as a heatmap (Fig 5A). The range of colocalization percentages above chance spanned from 1.11 ± 1.28% to 27.74 ± 5.70%. Thus, after chance correction some RNAs were rarely colocalized whereas others showed ~20–30 times more colocalization suggesting a difference in the propensity of RNA species to be colocalized. We hierarchically clustered the shared colocalization patterns which revealed three distinct clusters displaying consistently “high”, “intermediate”, or “low” levels of colocalization across all pairwise comparisons. Unexpectedly, levels of colocalization increased with RNA abundance such that the two most abundant dendritic RNAs in our dataset, *Camk2a*, *Ddn* and *Dlg4* (# of RNPs: *Camk2a*= 12,829 ± 1,646; *Ddn*= 11,114 ± 1,262, *Dlg4* = 6,426 ± 424, N=4 mice, Supp Fig. 2) exhibited uniformly high colocalization patterns with each of the other RNAs. RNAs with intermediate levels of abundance (*Calm1*= 6,451 ± 2,096, *Aco2* = 5,054 ± 450, *Psd* = 3,648 ± 764; N=4 mice, Supp. Fig 2) consistently demonstrated intermediary levels of colocalization across all pairwise comparisons. RNAs with relatively lower levels of abundance (*Pld3* = 3,457 ± 427, *Cyfip2* = 3,919 ± 725, *Adcy1* = 2,327 ± 407, *Bsn* = 3,157 ± 450, *Pum2* = 1,694 ± 208, *Ppfia3* = 1,162 ± 178) typically showed lower levels of colocalization across all pairwise comparisons. Representative images of high (*Camk2a*), intermediate (*Aco2*) and low (*Pum2*) levels of colocalization with *Psd* RNP are shown in Fig 5B, including the intersecting pixel overlaps for properly registered and rotated/chance images.

To visualize the influence of RNP abundance on colocalization, we plotted the % *Psd* colocalization above chance versus abundance in a correlation plot (Fig 5C). We found that abundance is highly correlated with the % *Psd* overlap (R^2^= 0.92). We observed the same pattern regardless of the expression of the RNA that is being colocalized (Supp. Fig. 4, *Ddn* (high) & *Pum2* (low). As pairwise colocalization cannot portray the scale of heterotypic RNP compositions, we then quantified the percentage of *Psd* RNPs that are localized singly or in association with at least one (dimers) or more species of RNAs (multimers) in both registered and rotated images (chance) (Fig 5D and Supp. Fig 5). The pie chart of *Psd* RNP compositions shows that only 8.13 ± 1.8% of *Psd* are not colocalized with any RNA (singleton) in our experimental dataset which is much lower than would be expected by chance (34.9 ± 5.4%) indicating that the majority of the *Psd* RNPs are localized as heteromeric RNPs that have at least two types of transcripts. *Psd* RNPs that are colocalized with only one other type of RNA (dimers) range from 0.23 ± 0.07% (*Psd*/*Ppfia3*) to 11.58 ± 3.12% (*Psd*/*Camk2a*) that also mirrors the abundance of the RNAs it colocalized with in both registered and rotated images. However, the percentage of *Psd/Camk2a* (11.58 ± 3.12%) dimers are much higher than chance (6.68 ± 1.1%) although all other Psd-dimers are almost at chance. Importantly, we observed that 70.25 ± 4.84% of *Psd* RNPs have at least three or more transcripts (multimers) of which the greatest fraction has *Camk2a* (57.91 ± 5.3%). Only 12.34 ± 2.9% of multimeric *Psd* RNPs had three or more types of transcripts that were without *Camk2a*, underscoring the dominating presence of *Camk2a* in both *Psd* dimers and multimers in our data. As opposed to higher percentage of Psd-singletons in chance quantification, the percentage of Psd multimers with and without Camk2a (34.89 ± 4.9%, 8.29 ± 1.6%) are noticeably lower than experimental images suggesting that it is unlikely for a given RNA to be present as a homomeric RNP not colocalized with other RNAs.

Furthermore, when other RNAs were quantified similarly, we found that an average of 88.6 ± 0.73% of each dendritic RNA is localized with at least one other RNA in our dataset compared to 65.62 ± 0.66% expected by chance (Supp. Fig. 6). Thus, only ~11.40 ± 0.73% of the dendritic RNAs are uncolocalized or more likely associated with other RNAs not visualized here. Combined together, our hiplex data demonstrate that 1) RNP size heterogeneity reflects dendritic RNPs composed of varying amounts of the same RNA and 2) the vast majority of RNPs are localized as multiple different species of RNAs (heteromeric RNPs) that scale with RNA abundance, and this effect stands significant despite being corrected for abundance.

## DISCUSSION

In this study, we examined the variety of flavors of neuronal RNPs and their co-assemblies in intact mouse hippocampus using high resolution multiplexed smFISH. First, we provide evidence supporting the substantial diversity of dendritic RNP compositions that encompasses varying amounts of a single RNA species and/or a combination of multiple different types of RNA species. Second, we demonstrate that dendritic RNAs targeted by the same trans-acting element, FMRP, tend to colocalize more than they do with RNAs not targeted by FMRP. Third, by simultaneously visualizing a dozen putative FMRP target mRNAs in an attempt to uncover whether transcripts selectively colocalize more or less based on any discernible molecular logic (molecular function or targeted location), we found that every RNA, regardless of its abundance, colocalizes more with highly abundant RNAs compared to RNAs lower in abundance. This result stands after correcting for the abundance of the two RNAs being compared. Lastly, we show limited evidence that dendritic RNPs are composed of only one type of RNA species. Our data also suggest that RNA abundance may be one of the crucial drivers of heteromeric RNA cargo assembly, a model that could potentially satisfy how neurons maneuver trafficking thousands of transcripts over long distances to maintain and modify neuronal communication.

### Dendritic RNPs contain varying amounts of a single RNA species

Neurons require the localization of thousands of different types of RNAs of variable abundance and subcellular distributions to support synaptic function. Yet, few studies have systematically characterized how dendritic RNAs are sorted into RNPs/ granules and their compositions that could support the delivery of thousands of RNAs encoding proteins involved in many different biological processes. Mikl and colleagues (Mikl et al., 2011) investigated the localization of *Map2* and *Camk2a* RNAs in hippocampal neurons in culture, showing that these mRNAs are present in dendrites in distinct RNPs, each containing as few as one or only a few copies of the same transcript with minimal colocalization between the two transcripts. Another study by Batish and colleagues (Batish et al., 2012), visualized pairwise combinations of eight dendritically localized transcripts with smFISH in hippocampal cultured neurons, also showing unimodal distribution of RNA puncta fluorescence intensities and ~4% of colocalization between pairs of RNAs, suggesting that mRNA molecules are trafficked singly and independently of others in neurons. In addition, there is evidence from in situ studies supporting RNAs localizing as unique populations of RNPs with distinct mRNA contents. Single molecule FISH detected **β**-actin mRNA in hippocampal culture neurons showed that mRNPs may contain multiple copies of **β**-actin mRNA and the copy number decreased with increasing distance from the cell soma (Park et al., 2014). Variation in size and intensity of individual *Camk2a*, *Arc* and *Ng (neurogranin)* RNPs were also reported by Gao et al. in developing neurons which is consistent with our observation (Gao et al., 2008). Furthermore, a recent study by Donlin-Asp and colleagues used molecular beacons in cultured neurons to track endogenous mRNAs, *Camk2a* and *Psd95*, and, in addition to detecting single mRNA transport events, they observed mRNAs in heterogeneous copy number states (Donlin-Asp et al., 2021). While extremely informative, most of these studies were done in primary neuronal cultures and limited in the species of dendritic RNAs investigated, demonstrating a need to evaluate RNP composition for the growing list of dendritically localized RNAs in intact neuronal circuits.

Our data, obtained using single- and multiplexed-smFISH to measure RNP sizes in three different cell types in the intact rat and mouse hippocampus (DG, CA1, CA2), corroborate previous observations in culture that RNP content varies from low-copy number RNAs to higher order homomeric RNA clusters of the same transcript (multiple copies of the mRNA derived from the same gene). While we cannot exclude the possibility that the differences in RNP sizes could somehow arise from the labeling or imaging techniques, we did not observe any particular round of imaging or fluorophore wavelength to behave in a certain way that would explain the observation. Further, it is difficult to imagine a technical explanation for observed differences within the same transcript species, unless there is a biological mechanism restricting access to specific populations of transcripts that is impenetrable to protease digestion. Observations from non-neuronal systems, such as drosophila RNA germ granules (Niepielko et al., 2018; Trcek et al., 2020) also indicate that localized RNAs sort into homotypic clusters. However, with the limited number of dendritically localized RNAs visualized for RNP composition, it is not yet clear whether the existence of distinct copy number states is a transcript-specific feature or a transcriptome-wide phenomenon. Our evidence of heterogeneous copy number RNPs within and across 15 dendritic RNAs is consistent with the idea that multiple RNP assembly states coexist for localizing RNPs, potentially providing flexibility to respond to a diverse range of synaptic inputs/ signals. Fractionation studies using RNA-pull down assays with brain lysates, neuronal culture lysates or synaptoneurosomal fractions suggest that mRNAs are present in monosomes, actively translating pools (polysomes) and translationally silent RNA granules (Hafner et al., 2019; Kanai et al., 2004; Krichevsky & Kosik, 2001). Whether different sized RNPs reflect their association with ribosomes or translational status is yet to be determined. Work on p-bodies and stress granules show that granule size correlates with increased granule stability (Moon et al., 2019). Further studies are needed to identify whether differences in RNP size and composition reflect different structural properties and/or functional RNP states.

### RNAs targeted by same RBP demonstrates preference for colocalization (may relate to cis sequence commonality but further experiments needed to confirm)

Previous work showed that RNAs with similar cis-acting A2RE sequences (*Camk2a*, *Arc* and *Ng*) were targeted by the same RBP, hnRNP A2, which was necessary and sufficient to mediate RNA granule assembly of these RNAs in cultured neurons (Gao et al., 2008). Observation in yeast cells using both transcript-specific RNA pulldowns and smFISH showed that RNAs that encode proteins functioning in a common biological pathway are multiplexed into the same RNP complexes (Nair et al., 2021). Consistent with this, our data visualizing three G-protein signaling dendritic RNAs also show that RNAs targeted by the same RBP (FMRP targets *Adcy1* and *Ppp1r9b*) demonstrated a bias for colocalization compared to an RNA that is not a demonstrated target of FMRP (*Rgs14*). However, we cannot exclude the fact that lower colocalization of *Adcy1*/ *Rgs14* could also be driven by lower *Rgs14* abundance in CA2 and CA1 compared to *Ppp1r9b*, as would be predicted by our Hiplex results. Further experiments using RNAs with comparable abundance and with and without similar FMRP binding motifs are needed to strengthen our interpretation.

### Majority of dendritic RNPs localize co-assembled with multiple species of RNAs

Our pairwise colocalization data, in both 3plex and Hiplex experiments, show ~20–30% colocalization significantly above chance between RNAs, similar to studies done before that looked at pairwise colocalization (Farris et al., 2014; Tübing et al., 2010). This number increased to ~80–90% (chance ~65%) when colocalization was assessed for a dozen RNAs, all targeted by the same RBP. However, our definition of colocalization is based on 2D overlapping in situ signals, which may over- or under-estimate the true level of colocalization. Thus, we cannot exclude the possibility that our techniques are failing to detect every instance of colocalization or detecting false positives due to the limits of our x-y resolution (250 nm). Therefore, additional super-resolution techniques (i.e. STORM) are required to prove whether any RNAs investigated in this study are physically interacting (<250 nm) within an RNP. However, similar to what we report here using isoform specific probes targeting *Shank2*, our previous work showed that probes targeting different regions of *Arc* RNA (*Arc* 5’ and 3’ UTRs) colocalized ~ 30% in rat hippocampal neuropil (Farris et al 2014). Colocalization in both instances was less than 100% likely due to the different efficiencies of the probes to bind, e.g. 5’UTR has more structure and detected fewer RNPs compared to the probe targeting the 3’UTR. However, there was a high degree of colocalization when more than one RNA transcript was present, i.e., in transcriptional foci in the nucleus where 100% colabeling of 5’ and 3’ probes was detected. Thus, we reason that this technique (RNAscope/Hiplex) is more limited in its ability to detect co-labeling of the same individual RNA transcript with two probes (~30%), perhaps due to steric hindrance or competition of the DNA-based labeling approach, but it is highly likely to detect colocalization when more than one transcript is being labeled (e.g. two transcripts of the same RNA or two distinct dendritically localized RNAs). RNPs containing multiple types of transcripts can possibly indicate a mechanism of transport that prefers efficiency over precision. There are multiple lines of evidence that show FMRP targets are differentially altered in the absence of FMRP at the level of RNA localization (Dictenberg et al., 2008; Miyashiro et al., 2003; Steward et al., 1998). It will be interesting for future studies to assess whether distinct RNP identity and compositions make the cargoes more or less vulnerable to FMRP loss. Moreover, there is compelling evidence of FMRP granule remodeling after synaptic activity to support local protein synthesis (Kharod et al., 2023; Sidorov et al., 2013). If the majority of RNPs are assemblies of many different types of RNA transcripts, how selectivity and specificity is achieved to supply nascent synaptic proteomes needs further investigation. *Camk2a* mRNA has been shown to interact with multiple other RBPs in addition to FMRP (RNG105, CPEB, and Staufen) (Moon et al., 2019; Ortiz et al., 2017; Shiina et al., 2005; L. Wu et al., 1998). In our data, *Camk2a* was also present in the highest percentage of heteromeric (both dimeric and multimeric) RNPs. It seems reasonable to hypothesize that *Camk2a*-containing heteromeric RNPs might show some degree of selectivity that is dependent upon which RBP is present in the granule.

### RNP composition tracks with RNA abundance

Studies in neurons and non-neuronal cell types have shown that RNA localization is influenced by several factors including, but not limited to, sequence length, stability and degradation (Farris et al., 2014; Lipshitz & Smibert, 2000; Middleton et al., 2019; Wu et al., 2020). In developing neurons (DIV 8 and DIV 15) in culture, localization of RNP granules have been shown to be affected by the constituents of the granules as demonstrated by visualizing staufen-containing and puralpha-containing granules and their colocalization in dendritic spine (Mitsumori et al., 2017). Our Hiplex data, using p17 mouse brains, shows that pairwise colocalization across RNA species tracks with abundance of dendritic mRNAs implying availability of transcripts can possibly dominate dendritic RNP co-packaging. This is consistent with data from Wang & colleagues who used MERFISH (Multiplexed Error-Robust Fluorescence In Situ Hybridization- a single-cell transcriptome imaging method) and spatial proximity clustering in cultured (DIV 18) neurons and showed that RNAs with high abundance spatially cluster together (*Camk2a, Ddn, Dlg4, Ppp1r9b, Shank1, Palm*) (Wang et al., 2020). *Camk2a, Ddn* and *Pp1r9b* transcripts, in addition to being highly abundant, are also the mRNAs that are highly translated in the dendrite as shown by their high ribosomal densities/ associations (Glock et al., 2020). During neuronal maturation, both in culture and *in vivo*, alteration in the availability of RNA has been shown to affect Dead-Box helicase 6 (DDX6)-positive physiological granule assembly indicating RNA supply as one of the critical drivers for transport RNPs (Bauer et al., 2022). Fatimy & colleagues, through live imaging of FMRP containing granules, have demonstrated that smaller RNPs fuse to form larger RNA granules and dissociate again to smaller RNPs before moving through the spine neck in hippocampal neurons in culture (Fatimy et al., 2016). Experiments in germ cell granules (non-neuronal) (Niepielko et al., 2018) show that highly abundant RNAs have higher seeding events to initiate homomeric RNP formations and subsequently recruit other mRNAs to the RNPs. A plausible explanation of our findings could also be that highly abundant dendritic RNAs have greater advantage of being present in small RNPs that then assemble into larger RNA transport granules (as found by Krichevsky and coworkers) (Krichevsky & Kosik, 2001). This would indicate that some general mechanisms exist influencing RNP compositions that transcend cell morphology (Goering et al., 2023). Further investigation into the sequence-specific features (5’ and 3’ UTRs length and the RBP-binding motifs/ cis sequences within them, RNA modifications for example: m6A methylation) of the twelve mRNAs in our dataset is needed to determine whether and/or how highly abundant dendritic RNPs sequester other classes of RNPs into the same granule (Niepielko et al., 2018).

In summary, by taking advantage of advanced tools in spatial imaging, the results of this study provide strong evidence for diversity and heterogeneity in dendritic RNPs in intact neural circuits and suggest a possible role of highly abundant mRNAs in steering the packaging of RNA packages for translocation. Subsequent studies will elucidate how different flavors of RNPs influence the localization and translation of messages at synapses.

## METHODS

### Animals

Sexually naive adult female Sprague Dawley rats were used for the Arc dilution studies in Fig. 1. Both male and female C57BL/6J were used at 8–16 weeks of age for Fig. 2 & 3 and at p17 (post-natal day 17) age for Fig 4 & 5. Animals were group-housed under a 12:12 light/dark cycle with access to food and water ad libitum. All procedures were approved by the Animal Care and Use Committee of Virginia Tech or University of California Irvine and were in accordance with the National Institutes of Health guidelines for care and use of animals.

### Stimulation Paradigm

The stimulation paradigm used in Fig. 1 was as previously described (Steward and Worley, 2001) with the following modifications. Briefly, an electroconvulsive seizure (ECS) was induced in unanesthetized adult female Sprague Dawley rats by delivering AC current (60Hz, 40mA for 0.5s). Anesthesia was induced immediately after ECS by i.p. injection of 20% urethane. The animals were then placed in a stereotaxic apparatus and a stimulating electrode was positioned to selectively activate one side of the medial perforant path projections (1.0 mm anterior to transverse sinus and 4.0 mm lateral from the midline). The electrode depth was empirically determined to obtain a maximal evoked response in the dentate gyrus at a minimal stimulus intensity, typically 3–4mm deep from the cortical surface. A recording electrode was positioned in the molecular layer of the dorsal blade of the dentate gyrus (3.5 mm posterior from bregma, 1.8 mm lateral from the midline, 3–3.5 mm from the cortical surface based on evoked responses generated by stimulation). Single test pulses were then delivered at a rate of 1/10 s for 20 minutes to determine baseline response amplitude. Two hours after the ECS delivery, high frequency stimulation (trains of eight pulses at 400hz) were delivered at a rate of 1/10 s. After 60 minutes the brain was removed and flash frozen. Brains were embedded in OCT and sectioned in the coronal plane on a cryostat at 20 μm and processed for FISH as described below.

### Fluorescent in situ hybridization (FISH)

FISH was performed as previously described (Guzowski et al., 1999; Farris et al 2014) to examine *Arc* mRNA puncta in dendritic fields of the dentate gyrus. For the dilution experiments, a 1X saturating stock of full length dig-labeled *Arc* probe was serially diluted with full length unlabeled *Arc* probe at 1:2 and 1:4.

### Quantitative Analyses of *Arc* puncta number and size

Sections processed for FISH were imaged across the molecular layer of the dentate gyrus at 63X using a confocal microscope. The size and number of *Arc*-positive puncta were determined using imageJ particle analysis function (NIH). Briefly, the images were overlaid using DAPI and a region of interest (ROI) was determined so as to count each *Arc*-puncta only once. The entire image was then set to an unbiased threshold and watershed to segment individual *Arc*-puncta. The ROI was then cropped out of the original image and subjected to particle analysis. The number and feret’s diameter of *Arc*-puncta were averaged across three sections from one animal and data are presented as mean +/− SEM across animals.

### Single Molecule Fluorescence in situ hybridization

Brains were embedded in OCT and sectioned in the horizontal plane (mouse studies) on a cryostat at 20 μm and processed for single molecule FISH according to the RNAscope Fluorescent Multiplex kit instructions (Advanced Cell Diagnostics, Hayward, CA). The following mus musculus specific probes were used with the RNAscope fluorescent multiplex reagent kit (Cat# 320850): Rgs14 (Cat #416651), *Adcy1* (Cat #451241), Shank2-Pan (Cat #513711, NM_001113373.3/ENSMUST00000105900.8), Shank2-O3 (Cat # 851661-C2, ENSMUST00000146006.2/NM_001113373.3), Shank2-O4 (Cat # 852961-C3, ENSMUST00000105900.8/NM_001081370.2), mouse 3 plex positive control (Cat # 320881), 3 plex negative control (Cat # 320871). Mus musculus specific probes used for Hiplex assay includes *Adcy1* (Cat #451241-T1), *Aco2* (Cat #1120581-T2), *Psd* (Cat #449711-T3), *Dlg4* (Cat #462311-T4), *Calm1* (Cat #500461-T5), *Bsn* (Cat #1119681-T6), *Camk2a* (Cat #445231-T7), *Pum2* (Cat #546751-T8), *Ddn* (Cat #546261-T9), *Pld3* (Cat #507241-T10)*, Ppfia3* (Cat #1119691-T11), *Cyfip2* (Cat #561471-T12), Hiplex positive control (Cat #324321) and negative control (Cat #324341).

### smFISH Image Acquisition and Quantification

All images were acquired on a confocal microscope using a 40X oil immersion lens. Acquisition parameters were set using 3plexed negative controls (cDNA probes against bacterial RNAs not present in mouse tissue) in each of the 3 channels (Alexa 488, Atto 550, Atto 647) so that any signal above the level of background was acquired. Area CA2 borders were identified using Pcp4 or Rgs14 as molecular markers; areas CA1, CA3 and DG were identified using defined anatomical locations. Each image was auto thresholded and particle number was quantified across the entire image (354.25 × 354.25μm) or a square region of interest (ROI, of constant size) using the analyze particle function in Fiji (NIH, v2) (Schindelin et al., 2012). Particle counts per subregion were averaged across sections (typically 2–4 sections per animal) to obtain one value per animal, and data are represented as mean particle count across animals ± SEM. All statistical analyses were carried out using Graphpad PRISM 7 software, and significance was determined using an alpha level of 0.05.

### Shank2 smFISH, image acquisition, and analysis

smFISH was performed according to the instructions provided in the kit (Cat# 320850). Probes labeling shank2e-long, shank2a-short and pan (both 2a and 2e) were imaged in channels with LEDs Alexa-647, Atto-555 and Atto-488 respectively. ROIs captured from CA2 cell body were 211 μm X 211 μm in x-y plane and 5μm in z (25 steps, step size: 0.21μm) at 63X magnification using a Leica thunder (Leica DMi 8) wide-field fluorescence microscope. Images were denoised and deconvolved in NIS elements AR to increase signal to noise ratio and remove background. After all computational processing steps for signal optimization, maximum projection was used for further analysis. A binary segmentation layer was created using the “bright spot” command in NIS AR based on the fluorescent intensity of the puncta. For overlapping signals across individual channels, additional binary layers were created combining binaries from individual layers. Data was then extracted to excel. Colocalization fraction was calculated as the percentage of overlapping puncta relative to the total number of puncta positive for that individual channel of interest. Example equation is as follows:

$$\frac{\text{Percentage}\;\text{of}\;\text{shank2}-\text{pan}\;\text{colocalzing}\;\text{with}\;\text{any}\;5^{\prime }\left(\text{shank}2\text{a}\;\text{or}\;\text{shank}2\text{e}\right)=\text{No}.\;\text{of}\;\text{shank}2-\text{pan}\;\text{puncta}\;\text{that}\;\text{overlaps}\;\text{with}\;\text{any}\;\text{puncta}\;\text{in}\;\text{shank}2\text{e}\;\text{or}\;\text{shank}2\text{a}}{\text{Total}\;\text{number}\;\text{of}\;\text{Shank}2-\text{pan}\;\text{puncta}}\times 100$$

For calculation of pixels that are randomly colocalized (chance), each image in a pair of comparisons was rotated 180 degrees and then the computation was repeated again and presented as ‘chance’ in fig 2D.

### Multiplex smFISH, image acquisition, and analysis

Mouse brains were flash frozen in chilled isopentane followed by blocking in OCT. 20μm cryosections were slide mounted for smFISH. smFISH was performed using RNAscope fluorescent multiplex reagent kit (# Cat info: ) as described in the protocol. 211 μm X 211 μm ROI (in x-y plane) of 5μm thickness Z-stack images (25 steps, step size 0.21μm) were acquired by a Leica thunder (Leica DMi 8) wide-field fluorescence microscope at 63X magnification (Numerical aperture 1.4, refractive index 1.51). CA2 was identified using Rgs14 and *Adcy1* labeling. *Rgs14, Adcy1* and *Ppp1r9b* were imaged using 488, 550 and 647nm LED. CA1 and CA2 proximal and distal dendritic regions were imaged from N=4 adult mouse hippocampus. Exported TIFF images were then processed using NIS elements AR. Each image including negative control was computationally processed by denoising and automatic deconvolution algorithm. A binary segmentation layer was created per channel on the post-processing max projection images based on fluorescent intensity and manually edited to best represent the data. Any signal overlapping plus 10% of DAPI was excluded from quantification to confirm only RNAs in the pyramidal neuron dendrites but not in glia or interneurons are included in the analysis. After manually editing each binary layer, size data was exported to excel and plotted with prism.

Size data of *Rgs14, Adcy1, Ppp1r9b* from proximal and distal dendrites of CA2 and CA1 was plotted in prism as individual histograms (size in X-axis and number of RNAs in y-axis) of each animal at first (data not shown). Although, total number of RNA puncta varied for each mRNA species from mouse to mouse, a consistent pattern of size distribution was noted when plotted as a percentage fraction for size distribution. Due to consistent patterns across dendritic subregions, data from proximal and distal dendrites were then combined to represent the size histogram as the relative percentage of RNAs of different sizes per hippocampal region (Fig 3. D&E). For RNPs of size 0.0 to 0.48μm^2^, bin width is kept at 0.12 and for RNPs of area>0.48μm^2^, binwidth is kept as 0.24 as plotted on the X-axis (Fig 3D & E). Anything equal to or greater than 5 μm^2^ is stacked into the same bin using overflow binning in excel.

Colocalization between individual channels was defined as touching or overlapping binary objects from two separate channels of interest. For object-based colocalization analysis, additional binary layers were created as “*Adcy1* mRNA puncta having any overlapping pixel from *Rgs14*” and “*Adcy1* mRNA puncta having any overlapping pixel from *Ppp1r9b*”. Number of *Adcy1* puncta that colocalized with either *Rgs14* or *Ppp1r9b* was then divided by the total number of *Adcy1* puncta and plotted as a percentage (Fig 3. F&G). To calculate the percentage of randomly overlapping pixels, *Adcy1* image was rotated 90 degrees and the steps of creating an additional binary layer that includes signal overlaps from Rgs14 and Ppp1r9b was repeated and the percentage was calculated against the total number of *Adcy1* particles.

### Hiplex smFISH, image acquisition and analysis

smFISH was performed on slide mounted 20μm sections using RNAscope Hiplex Assay V2(Cat# 324120). After fixation, samples were dehydrated in % ethanol and treated with protease IV for 30 mins. Samples were hybridized at 40°C with the twelve probes for 2 hours followed by signal amplification steps (check notes). T1-T4 fluorophores were added to image *Adcy1, Aco2, Psd* and *Dlg4* mRNAs in round one. For each round, 488, 550, 647 and 750 nm LED were used to image four RNAs at 63X magnification (Numerical aperture 1.4, refractive index=1.51). Leica Thunder epifluorescence microscope (Leica DMi 8) was used for imaging with recorded stage positions to acquire the same ROIs across rounds. *Adcy1* mRNA probe was in round one and used as a CA2 marker and DAPI signal was acquired using a 390 nm LED. After round one, coverslips were taken off by keeping slides in 4X SSC, fluorophores were cleaved and FFPE reagent was used to decrease background. Subsequently T5-T8 fluorophores were added to image *Calm1, Bsn, Camk2a* and *Pum2* in the second round. This was followed by similar steps of cleaving the fluorophores and background removal. For the final round, T9-T12 fluorophores were added to image *Ddn, Pld3, Ppfia3, Cyfip2* mRNAs. Exposure was adjusted in each round matching with the expression of individual RNAs but kept consistent across all animals. After completion of smFISH, fluorophores were cleaved and slides were washed in TBS for 2×5mins, blocked in TSA-blocking solution for 30 mins and incubated with anti-rabbit-FMRP primary antibody (1: 100, Abcam, Cat# ab17722, Lot# 632949982) at 4C for two consecutive nights. Subsequently slides were washed in TBS-T (0.05% Tween) for 3×5 mins and 2% H2O2 in TBS for 10 mins at room temperature. Following that, slides were incubated with goat-anti-rabbit HRP (1:250, Jackson Immunoresearch, Cat#111035144, Lot# 149770) for 2 hours at room temperature. Slides were washed in TBS-T before they underwent incubation with brand new TSA-Cy3 kit (1:50, Cat# NEL704A001KT, Lot# 210322048) for 30 mins at room temperature. Slides were then washed in TBS-T for several times and cover slipped with prolong gold antifade mounting medium. Images of FMRP immunostaining were done using tissue from N=2 animals, imaged using 550 LED and signals were adjusted using negative control slide. ROIs from proximal and distal dendrites of CA1 and CA2 were imaged that were 211μm X 211 μm in x-y plane and 5μm in z-plane (step size 0.21μm) at 63X magnification (Numerical aperture 1.4, refractive index 1.51). All z-stack images of individual channels and rounds were exported as TIFF images and converted to nd2 format for further processing on NIS elements AR. Denoising ai and Richardson-Lucy deconvolution algorithm was used to increase the signal to noise ratio and minimize background pixel intensity. Images were then max projected and registered using ACD RNAscope Hiplex image registration software based on the DAPI signal of each round. After registration, a composite image of 12 RNA channels and DAPI was created for segmentation and puncta size and colocalization analysis. Each channel was segmented to a binary layer based on fluorescence intensity threshold. Any binary object/fluorescence signal overlapping DAPI was removed from the analysis. Binaries were optimized manually for every channel to best represent the ground truth. Size data was exported per RNA channel for N=4 mice and then averaged for plotting on the heatmap (Fig 4C). For colocalization analysis, four 512×512 ROIs were cropped from the image. For each RNA, subsequent binary layers were created that would contain only RNA puncta having any overlap from each of the other channels/ RNAs in consideration. Thus, eleven binaries were created for each RNA channel to calculate the number of that RNA having pixel overlaps with any of the other eleven RNAs. This number was then expressed as a percentage of the given RNA (Supp Fig 2A.). Data from four 512×512 ROIs were averaged for each animal and then data from N=4 animals were averaged. For the quantification of the chance level of overlaps, the same method of calculation was followed only after rotating the image of the given RNA to 90 degrees right (Supp Fig 2B). Percentage of colocalization due to chance was subtracted from the percentage of colocalization calculated from experimental images and plotted in a heatmap (Fig 5A).

Calculation of Psd percent colocalization with any RNA in Hiplex dataset was done by creating a union layer of all intersect binary layers for Psd. Thus “*Psd* having *Camk2a* (RNA 1)”, “*Psd* having *Ddn* (RNA 2)”….” *Psd* having *Ppfia3* (RNA 11)” all layers were merged to create a union layer that includes Psd puncta that has pixel overlaps from any of the other 11 channels. This data was then exported for N=4 mice, averaged and plotted with abundance of RNAs in Fig 5C. Similarly, correlation of RNP colocalization with RNA abundance was calculated for *Ddn* and *Pum2* (Supp. Figure 4).

### Statistical analyses

All statistical analyses were done using graphpad prism (Graphpad Prism 10) with a significance level of 0.05 or lower (**α**=0.05).

## Supplementary Material

1

## MAIN POINTS

- Dendritic RNAs exist as diversely composed RNPs that contain variable quantities of an individual transcript as well as combination of multiple different transcripts.
- RNAs targeted by the same RBP display a bias to localize together as opposed to being colocalized with a non-target RNA.
- Dendritically localized RNAs, regardless of their individual abundance, colocalize more with highly abundant RNAs compared to less abundant RNAs, even after correcting for random colocalization based on the abundance of RNAs being compared.
- There is limited evidence of dendritic RNPs being composed of a single RNA species, suggesting that dendritic RNA cargoes are multimeric complexes *in vivo* and RNA abundance possibly drives the assembly of heteromeric RNA cargos, facilitating the transport of thousands of transcripts to distal compartments.
