# Dendritically localized RNAs are packaged as diversely composed ribonucleoprotein particles with heterogeneous copy number states

**DOI:** 10.1101/2024.07.13.603387

**Published:** 2024-08-25

**Authors:** Renesa Tarannum, Grace Mun, Fatima Quddos, Sharon A. Swanger, Oswald Steward, Shannon Farris

**Affiliations:** 1 Fralin Biomedical Research Institute at Virginia Tech Carilion, Center for Neurobiology Research, Roanoke, Virginia; 2 Translational Biology, Medicine & Health Graduate Program, Virginia Tech, Blacksburg, Virginia; 3 University of California Irvine, Irvine, California; 4 Department of Biomedical Sciences & Pathobiology, Virginia-Maryland College of Veterinary Medicine, Virginia Tech, Blacksburg, Virginia; 5 Virginia Tech Carilion School of Medicine, Roanoke, Virginia

**Keywords:** mRNA localization, Neurons, RNA granule, RNP composition, Multiplex imaging

## Abstract

Localization of mRNAs to dendrites is a fundamental mechanism by which neurons achieve spatiotemporal control of gene expression. Translationally repressed neuronal mRNA transport granules, also referred to as ribonucleoprotein particles (RNPs), have been shown to be trafficked as single or low copy number RNPs and as larger complexes with multiple copies and/or species of mRNAs. However, there is little evidence of either population in intact neuronal circuits. Using single molecule fluorescence in situ hybridization studies in the dendrites of adult rat and mouse hippocampus, we provide evidence that supports the existence of multi-transcript RNPs with the constituents varying in amounts for each RNA species. By competing-off fluorescently labeled probe with serial increases of unlabeled probe, we detected stepwise decreases in *Arc* RNP number and fluorescence intensity, suggesting *Arc* RNAs localize to dendrites in both low- and multiple-copy number RNPs. When probing for multiple mRNAs, we find that localized RNPs are heterogeneous in size and colocalization patterns that vary per RNA. Further, localized RNAs that are targeted by the same trans-acting element (FMRP) display greater levels of colocalization compared to an RNA not targeted by FMRP. Simultaneous visualization of a dozen FMRP-targeted mRNA species using highly multiplexed imaging demonstrates that dendritic RNAs are mostly trafficked as heteromeric cargoes of multiple types of RNAs (at least one or more RNAs). Moreover, the composition of these RNA cargoes, as assessed by colocalization, correlates with the abundance of the transcripts even after accounting for the expected differences in colocalization based on expression. Collectively, these results suggest that dendritic RNPs are packaged as heterogeneous co-assemblies of different mRNAs and that RNP contents may be driven, at least partially, by highly abundant dendritic RNAs; a model that favors efficiency over fine-tuned control for sustaining long-distance trafficking of thousands of messenger molecules.

## INTRODUCTION

Neuronal morphology is incredibly complex, and in order for neurons to function efficiently RNA transcripts need to be delivered to distant sites for on-demand translation. In particular, RNA localization to synapses and subsequent local translation are required for the synaptic plasticity underlying learning (Bradshaw et al., 2003; Huber et al., 2000). Dysregulation of these processes is a common cause of intellectual disability and autism (Fernandopulle et al., 2021; Huber et al., 2002). Thus, uncovering how mRNA cargoes are delivered to and locally regulated at the synapse is central to our understanding of the molecular basis of learning and memory. Furthermore, studying the fundamental principles of neuronal mRNA localization can uncover key aspects of post-transcriptional regulation, which could be applicable to various other organisms and cell types, such as yeasts, drosophila germ granules, cardiomyocytes, etc., that use compartmentalization for gene regulation (Lewis et al., 2018; Martin & Ephrussi, 2009).

Early studies, prior to the use of modern molecular tools, detected only a handful of RNAs localized in neuronal dendrites (Steward & Schuman, 2003). More recent studies using compartment specific deep-sequencing, however, have revealed the presence of thousands of RNA species localized in the hippocampal neuropil (Cajigas et al., 2012; Farris et al., 2019). These localized mRNAs are assembled and transported as ribonucleoprotein particles (RNPs, otherwise known as RNA granules), which are dynamic, spherical, membraneless macromolecular complexes of translationally inert RNAs, RNA-binding proteins (RBPs), and translational machinery (Kiebler & Bassell, 2006; Knowles et al., 1996). Neuronal RNPs have been shown to be actively transported by conventional kinesins for targeted delivery to subcellular destinations (Köhrmann et al., 1999). Further, bidirectional trafficking of RNPs within dendrites has also been observed by many laboratories (Bauer et al., 2019; Braselmann et al., 2020; Dynes & Steward, 2007; Knowles et al., 1996). Purified RNA granules from brain lysates or dissociated cultured neurons are composed of many different RNAs (Fritzsche et al., 2013; Heraud-Farlow et al., 2013; Kanai et al., 2004) and RBPs (Fritzsche et al., 2013). These biochemically isolated endogenous RNA granules have overlapping RBP constituents and cargo mRNAs, as well as protein and RNA interactors that are exclusive to specific types of granules. However, due to limitations in multi-color labeling, simultaneous visualization of several RNP components has been technically challenging. Thus, there is limited evidence on the heterogeneity (how much of a given RNA) and diversity (how many types of different RNAs) in composition of RNPs as well as their spatial distribution in intact neural circuits.

Several different models have been proposed to explain how RNAs are assembled into RNPs to dictate their destination. One hypothesis is that RNAs are transported as single or low copy number molecules per RNP. This model was supported by experiments performed using single-molecule FISH in cultured neurons which revealed that individual dendritic RNPs, whether in transport or localized, might carry no more than one or two molecules of a specific type of transcript based on few instances of colocalization between different mRNA species (Batish et al., 2012; Park et al., 2014). Our published work visualizing two dendritically localized mRNA species, *Arc* and *Camk2a*, in the adult rat hippocampus also indicated relatively minimal colocalization, consistent with low mRNA copy number/ contents in transport RNPs (Farris et al., 2014). However, this model is challenged by recent studies that have identified thousands of RNA species in distant dendritic compartments (Ainsley et al., 2014; Cajigas et al., 2012; Farris et al., 2019). Selective delivery of low copy number RNPs appears at odds with sustaining the localization of thousands of diverse mRNAs with varying abundance and encoded protein functions. Immunoprecipitation studies from brain lysates suggest that mRNAs associate selectively within distinct RNA granules that can contain many different types of transcripts (some RNAs overlap and some are exclusive to each type of granule) (Elvira et al., 2006; Fritzsche et al., 2013; Kanai et al., 2004). Although this model seems plausible and efficient for localizing vast amounts of RNAs, these studies are technically limited by the lack of spatial resolution and non-specific RNA interactions. Addressing this question requires subcellular-resolution imaging of many endogenous molecules at once, which is now technically feasible using highly multiplexed, single molecule fluorescence in situ hybridization with iterative imaging (HiPlex smFISH).

In this paper, we used single, 3-color, and HiPlex smFISH to characterize the compositions of neuronal transport RNPs in rodent hippocampal neuropil. Hippocampus, a brain region critical for learning and memory, has a laminar architecture that affords the opportunity to catalog localized RNAs and visualize their subcellular distribution, in detail and in situ, in the dendrite-enriched neuropil layer. Here we show that, based on heterogeneity in RNP sizes, individual RNPs are likely composed of both single and multiple copies of individual RNAs, consistent with low and high copy number RNP populations. We also show that dendritic RNAs encoding proteins in the same signaling pathway exhibit preferential colocalization in the same RNP when targeted by the same RBP. Lastly, simultaneous visualization of a dozen localized mRNAs that are targeted by the same RBP revealed that the vast majority of dendritic RNAs are localized as multimeric RNP complexes. These complexes contain at least three or more different RNA species. The composition of multimeric RNP complexes showed a positive correlation between RNA abundance and colocalization. Collectively, these data provide evidence that dendritic RNAs localize into distinct subpopulations with varying RNA contents and colocalization patterns that scale by abundance, thus adding new insights into the diversity of constituents and heterogeneity of RNA copy number states of neuronal transport RNP compositions.

## RESULTS

### *Arc* RNPs contain multiple copies of *Arc* transcripts

**Fig 1:**
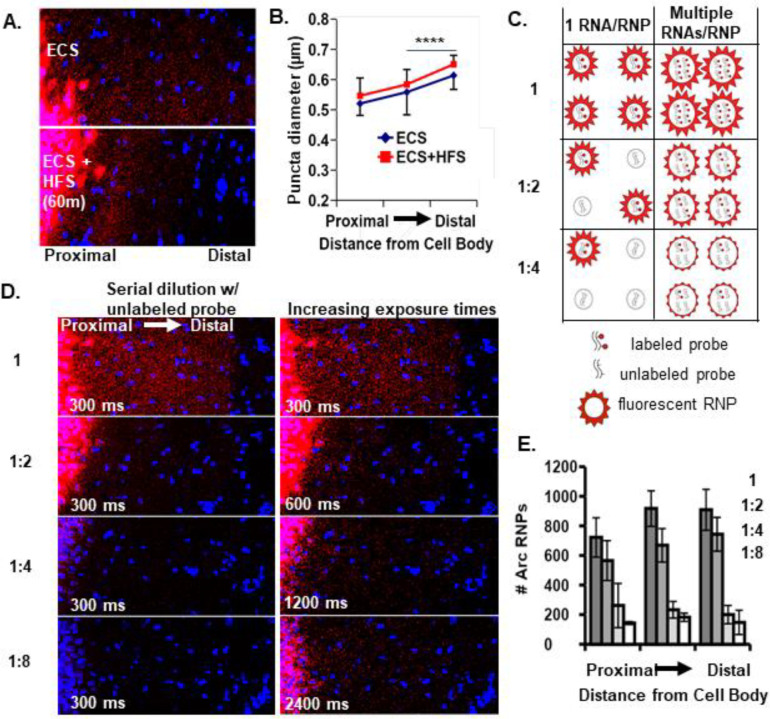
*Arc* RNP number, intensity, and size reflect multiple pools of RNPs. **A.** Representative images of *Arc* mRNA localization to the middle molecular layer of the rat dentate gyrus following ECS and 60 min unilateral HFS. **B.** Quantification of the size of localized *Arc* mRNA puncta (HFS condition) vs. non-localized puncta (ECS) across the molecular layer (middle molecular layers of HFS vs ECS, p>0.05; HFS or ECS middle molecular layer vs outer molecular layer, ****p<0.0001, KS test). **C.** Possible outcomes of serial probe dilution on single and multiple copy number *Arc* RNPs in terms of number and size of fluorescent RNPs. **D.** Representative images after ECS only labeled with 1X undiluted *Arc* probe or serially diluted with unlabeled *Arc* probe and imaged with identical exposure times, 300 ms,(Left). Images acquired with doubling exposure times (600, 1200, 2400 ms) revealed undetected RNPs at 300 ms (Right), indicating a decrease in RNP intensity as would be expected with multiple RNAs per RNP. **E.** Quantification of *Arc* RNP number for each dilution at 300 ms. A stepwise decrease in *Arc* RNP number suggests single RNA RNPs.

To begin to address whether neuronal RNAs are trafficked into dendrites singly and/or in multiple copy number RNPs, we investigated the RNP properties of the well-known dendritically localized mRNA *Arc* (activity-regulated cytoskeleton-associated mRNA). *Arc* mRNA expression in dentate granule (DG) cell dendrites is unique in that it is tightly regulated by activity-dependent transcription and degradation (Farris et al 2014). At baseline, dendritic *Arc* expression is low or absent in most dentate granule cells, but after a single electroconvulsive shock (ECS) *Arc* mRNA is rapidly transcribed (within ~3 minutes) and transported throughout the DG dendritic laminae by 30 min to 1 hour (Steward et al 1998). Given the short half-life of *Arc* mRNA (~45 min (Rao et al., 2006), the prolonged presence of *Arc* mRNA in DG dendrites (e.g. at 2 hours) is maintained by ongoing transcription and dendritic transport (Farris et al., 2014). Subsequent unilateral high frequency stimulation (HFS) of the entorhinal cortical perforant path inputs to the DG further boosts *Arc* transcription and leads to the accumulation of newly transcribed *Arc* RNA selectively near the activated synapses in the middle molecular layer and a depletion of *Arc* mRNA from the outer molecular layer (Steward et al 1998; Farris et al 2014). Using this stimulation paradigm (ECS + HFS) on adult female rats and fluorescence in situ hybridization (FISH), we assessed the size and number of dendritically-localized (ECS) and synaptically-localized (HFS) *Arc* mRNA puncta as putative RNPs (Fig. 1). We found that *Arc* RNPs are generally of similar size, as measured by feret’s diameter, whether they are localized to dendrites or targetted to recently activated synapses (Fig 1AB, middle molecular layers of ECS vs HFS, p >0.05 KS test). RNPs are larger in distal versus proximal dendrites under both conditions (ECS and ECS+HFS, p <0.0001, KS test). These data suggest that resident and newly transcribed dendritic *Arc* RNPs contain a similar amount of *Arc* RNA per RNP.

Next, in order to assess the transcript occupancy of *Arc* RNPs, we measured *Arc* RNP size and number in rats that received an ECS only (dendritically localized) after serial dilution of 1X labeled *Arc* probe with unlabeled (cold) *Arc* probe (1:2, 1:4, 1:8). We reasoned that a stepwise decrease in *Arc* RNP number would reflect singly transported RNAs that were no longer detectable when half, or fourth or eighth of the probe was labeled (Fig. 1C). Alternatively, a decrease in apparent size of *Arc* RNPs would reflect a reduction in the number of labeled transcripts from multiple copy *Arc* RNPs (Fig. 1C). When acquiring images using identical acquisition parameters (optimized for 1X labeled probe), we detected a stepwise decrease in *Arc* RNP number, with the largest drop off between one half and one fourth cold probe dilution (Fig. 1DE). These data are consistent with a population of low copy number (2–4) *Arc* RNPs. However, when we doubled the exposure time after each dilution, we qualitatively saw an increase in the number of *Arc* RNPs indicating that a proportion of the *Arc* RNPs labeled with cold probe dropped below the detection threshold (Fig. 1D), consistent with a population of *Arc* RNPs containing multiple copies of *Arc* transcripts. Collectively, the data suggest that there are multiple populations of *Arc* RNPs, those with low and high *Arc* copy numbers that exist in DG dendrites. Given that we did not detect any changes in RNP size after HFS, we assume this finding would translate to synaptically targeted *Arc* RNPs.

### smFISH probes can detect RNA colocalization

**Fig 2.**
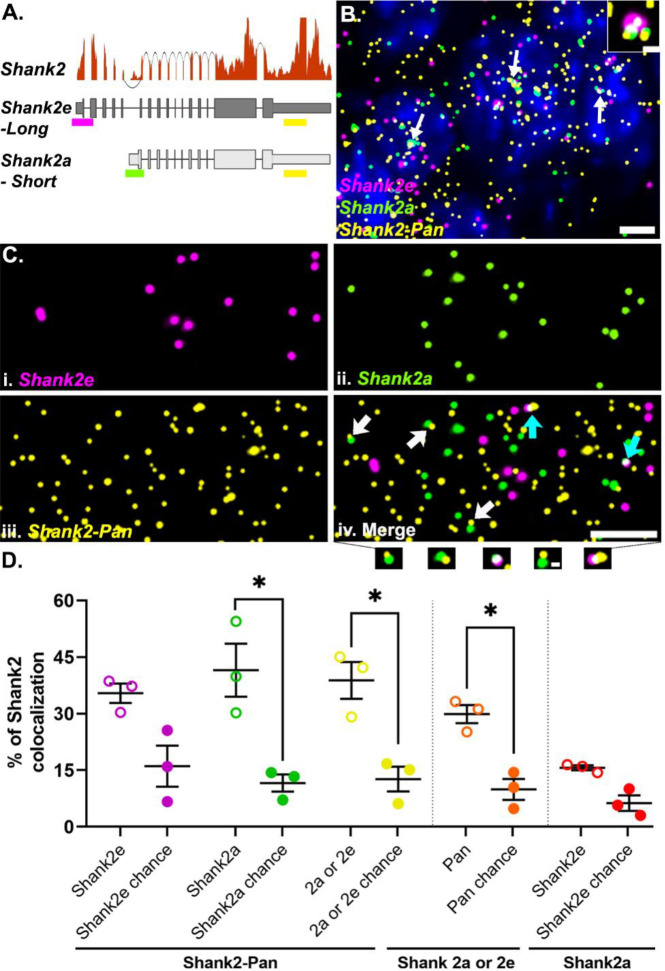
*Shank2* isoform-specific 5’ probes are highly colocalized with the Pan 3’ probe. **A.** *Shank2* isoform gene models with RNAseq read depth data showing the relative expression levels in hippocampal CA2. Sequences from either long (*Shank2e*) or short (*Shank2a*) transcripts targeted by different 5’ probes (magenta and green, respectively) and both targeted by the Pan 3’ probe (yellow) are shown. **B.** Representative image of the three *Shank2* probes in CA2 cell bodies. Nuclei are labeled with DAPI (blue). White arrows indicate example transcriptional foci. Dashed white box is the inset showing a transcriptional focus labeled by all three probes. **C.** High-magnification images of (i) *Shank2e*, (ii) *Shank2a*, (iii) *Shank2*-Pan and (iv) the merged image. Arrows indicate example colocalization of *Shank2*-Pan 3’ probe with either *Shank2e* 5’ probe (cyan arrows) or *Shank2a* 5’ probe (white arrows) as shown below. **D.** Quantification of the percent colocalization between the *Shank2e* (magenta) and *Shank2a* (green) or both (yellow) with the *Shank2*-Pan probe (open circles) compared to that expected by chance (closed circles). The percent of *Shank2*-Pan colocalized with either *Shank2a* or *Shank2e* (orange) compared to chance and the percent of *Shank2e* colocalizing with *Shank2a* (red) compared to chance, many of which are transcription foci, as shown in B. Error bars indicate SEM; N=3 mice; * denotes p <0.05 from paired one-tailed t-test. Scale bars: B) 5μm, 1μm, C) 5μm, 0.5μm

In addition to dendritic RNPs consisting of low or multiple copies of the same RNA (homomeric), we wanted to test whether dendritic RNPs are composed of multiple species of RNAs (heteromeric) as described *in situ* for established RNA granules like germ plasm granules (Trcek et al., 2015) or p-bodies (Ford et al., 2019; Teixeira et al., 2005) and identified biochemically for neuronal transport granules (Elvira et al., 2006; Fatimy et al., 2016; Fritzsche et al., 2013; Heraud-Farlow et al., 2013). In order to assess the colocalization of different RNA transcripts, we first needed to confirm that we can reliably detect colocalized smFISH signals. To test this, we took advantage of the fact that there are (at least) two isoforms of *Shank2* expressed in hippocampal area CA2 (Farris et al 2019). The two isoforms are generated via alternative 5’ promoters and thus differ in their 5’ untranslated regions (UTRs), but have identical 3’ UTRs (Jiang & Ehlers, 2013; Monteiro & Feng, 2017). Using isoform specific probes targeted to the two distinct 5’ UTRs (*Shank2e*-long and *Shank2a*-short) and a pan *Shank2* probe targeted to the common 3’UTR (Shank2-pan, Fig. 2A), we calculated the percentage of colocalized RNPs, defined as puncta in separate channels overlapping by at least 1%. In agreement with RNAseq expression data (Fig. 2A), we detected *Shank2* expression from all three probes in area CA2 (Fig. 2BC). In general, the *Shank2*-pan probe detected more *Shank2* RNPs than the 5’ UTR probes combined (# of RNPs: *Shank2*-pan = 3056 ± 472, *Shank2e* = 1122 ± 268, *Shank2a* = 1463 ± 79, N= 3 mice), either due to the (presumed) greater accessibility of the 3’ UTR from less RNA secondary structure compared to the 5’ UTRs, or potentially due to expression of other isoforms that include the 3’UTR but not either of the two 5’UTRs (e.g. *Shank2C*, Monteiro & Feng, 2017) that cannot be resolved via short-read sequencing. We found that nearly 40% of *Shank2e* (35.41 ± 2.57%) or *Shank2a* (41.53 ± 7.05%) colocalizes with the *Shank2*-pan probe (Fig. 2D). The *Shank2*-pan probe also colocalizes with either 5’ UTR probe at ~30% (29.88 ± 2.42%). The fact that this relative percentage is not greater than the colocalization of the individual 5’ UTR colocalization is due to both the greater number of *Shank2*-pan RNPs (described above) and instances where all three probes colocalize at presumed transcriptional foci (Fig. 2B, inset). These data are consistent with our previous findings, where ~30% of 5’ and 3’ *Arc* RNA probes colocalize in the dendrites of dentate granule cells in rats (Farris et al., 2014). To account for the amount of colocalization that occurs by chance and is influenced by differences in individual transcript expression levels, we remeasured colocalization for each probe pair after rotating one of the channels 180 degrees. In most cases, we detected a significantly greater percent colocalization than is expected by chance (Fig. 2D). In the instance where percent colocalization is near chance, as with the two 5’ probes, we assume this to indicate that these two transcripts do not colocalize often. In summary, our method is able to reliably detect colocalization of two probes targeted to the same RNA, which is a higher bar than for detecting two RNAs within the same RNP, which we assess below.

### Dendritic RNAs encoding G protein signaling proteins show multimodal size distributions and different patterns of colocalization

**Fig 3.**
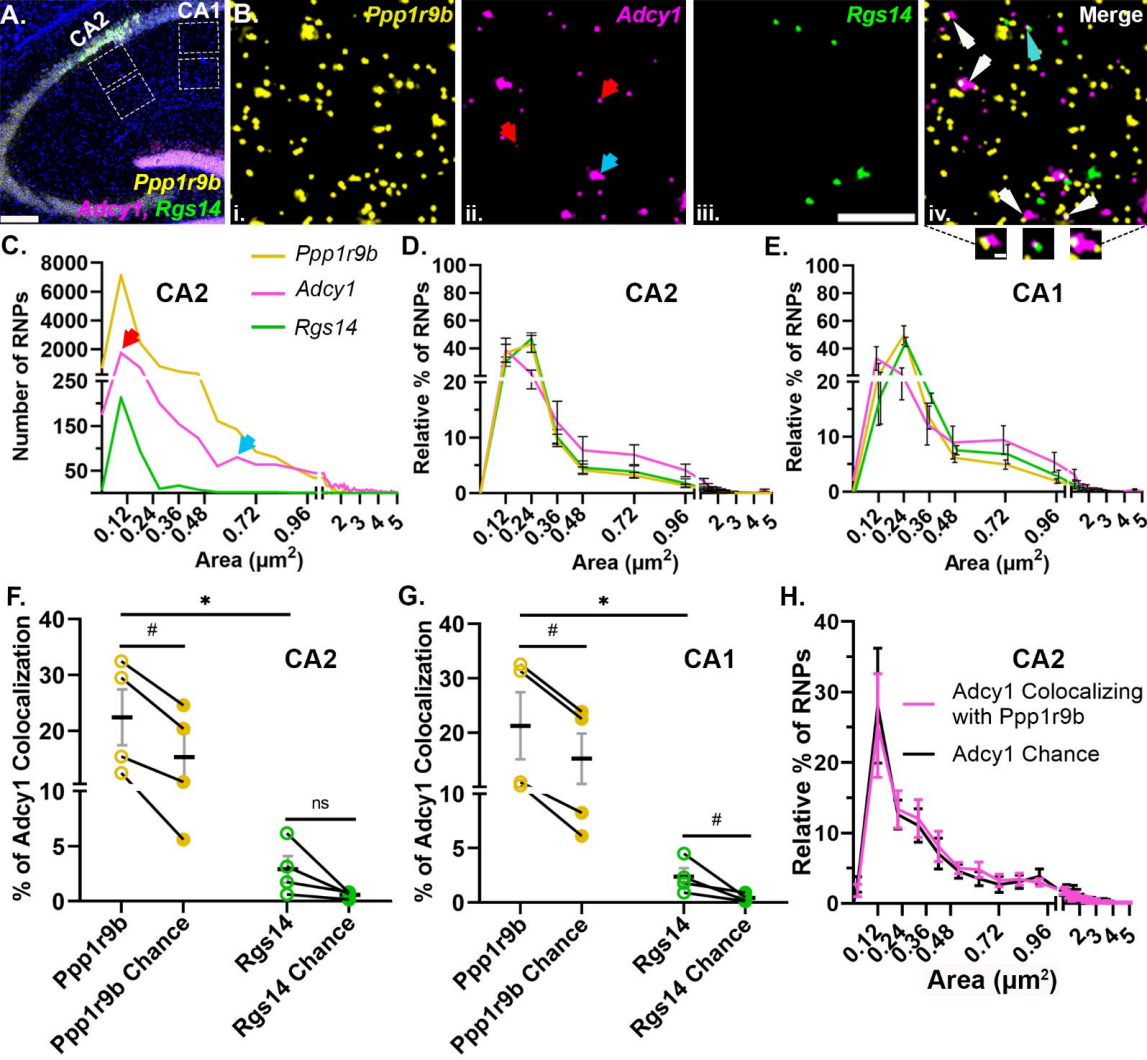
G protein signaling dendritic RNAs show heterogeneity in size distribution and colocalization patterns. **A.** Representative image of *Ppp1r9b* (yellow), *Adcy1* (magenta) and *Rgs14* (green) mRNAs and nuclei (in blue) in adult mouse hippocampus. Dashed white boxes are regions analyzed from CA1 and CA2 proximal and distal dendrites. **B.** High-magnification representative images of (i) *Ppp1r9b*, (ii) *Adcy1*, (iii) *Rgs14* and (iv) merged in CA2 distal dendrites. Arrows in red (small sized puncta) and blue (large sized puncta) indicate two distinct populations of *Adcy1* mRNA based on the size of the fluorescent puncta. (iv) Arrows indicate colocalization of *Adcy1* RNP with either *Ppp1r9b* RNP (white) or *Rgs14* RNP (cyan). Three example images of RNP colocalization in the callout section. **C.** Histogram from a representative mouse showing the size distributions of *Rgs14*, *Adcy1* and *Ppp1r9b* RNPs measured as the size of fluorescent puncta in CA2 distal dendrites. Red and blue arrows denote the two populations of *Adcy1* RNPs based on size. **D & E.** Relative percent histograms showing the average size distributions of *Rgs14*, *Adcy1* and *Ppp1r9b* from N=4 mice in CA2 and CA1 dendrites. Error bars indicate SEM. **F & G.** Quantification of the percent of *Adcy1* RNPs colocalizing with *Ppp1r9b* (yellow, open circles) and *Rgs14* (green, open circles) in CA2 (*Adcy1*/*Ppp1r9b* = 22.47 ± 4.99%; *Adcy1*/*Rgs14* = 2.93 ± 1.2%, p = 0.038, paired two-tailed t-test) and CA1 dendrites (*Adcy1*/*Ppp1r9b* = 21.24 ± 6.17%; *Adcy1*/*Rgs14* = 2.38 ± 0.76%, p = 0.047, paired two-tailed t-test). The percent colocalization expected due to chance was quantified by rotating the *Adcy1* image 90 degrees and calculating the percentage of puncta overlapping with *Ppp1r9b* (yellow, closed circles) or *Rgs14* mRNAs (green, closed circles). The percentages of *Adcy1* RNPs colocalizing with *Ppp1r9b* or *Rgs14* were significantly greater than what would be expected by chance (*Ppp1r9b* CA2: 22.47 ± 4.99%, chance 15.37 ± 4.30%, p =0.003; *Ppp1r9b* CA1: 21.24 ± 6.17%, chance 15.18 ± 4.64%, p= 0.016; *Rgs14* CA2: 2.93 ± 1.2%, chance 0.6±0.15% p= 0.06; *Rgs14* CA1: 2.378 ± 0.76%, chance 0.41 ± 0.18%, p= 0.0405, paired one-tailed t-tests) **H.** Size distribution histogram for the subpopulation of *Adcy1* RNPs that colocalize with *Ppp1r9b* shows there is no size bias for colocalization with *Ppp1r9b*. Error bars indicate SEM. Scale bars: A) 200μm, B) 5μm, 0.5μm.

Once we demonstrated that our method can reliably detect colocalization when we expect it, we explored whether known dendritic mRNA transcripts localize singly or in association (colocalized) with each other as heteromeric RNPs. We started with known dendritic mRNAs that encode signaling proteins in the same biological pathway (i.e. G-protein signaling), rationalizing that this might be an efficient way to co-assemble RNAs for transport to a similar destination or signaling hub. We performed smFISH for three known hippocampal dendritic RNAs within the G-protein signaling pathway, *Rgs14* (Farris et al., 2019; Squires et al., 2018), *Adcy1* (Farris et al., 2019; Lein et al., 2007) and *Ppp1r9b* (Hale et al., 2021; Zeitouny et al., 2023). Consistent with previous observations, all three mRNAs are localized throughout the dendritic laminae, in both proximal and distal dendritic regions of the neuropil in CA2 and CA1 cell types of the hippocampus (Fig. 3AB). However, each mRNA expressed a distinct dendritic expression pattern, including differences in RNP number and size, that did not differ by cell type. Quantification of the number and pixel size of fluorescent puncta of individual RNPs revealed that their expression varies from hundreds (*Rgs14*) to thousands (*Adcy1* and *Ppp1r9b*) of transcripts (Fig 3C). The distal dendrites exhibit a significantly lower abundance of *Rgs14* RNPs compared to the proximal dendrites in CA1 but not in CA2 (# of *Rgs14* RNPs: CA2 proximal = 784 ± 145 and distal = 177 ± 66, p = 0.053; CA1 proximal = 413 ± 38 and distal = 133 ± 36, p = 0.026, N=4 mice, paired two-tailed t-tests), while the abundance of *Adcy1* and *Ppp1r9b* RNPs remains relatively equivalent across layers of both cell types (# of *Adcy1* RNPs: CA2 proximal = 2993 ± 939 and distal dendrites = 2683 ± 307, p = 0.386; CA1 proximal = 2138 ± 798 and distal dendrites = 2500 ± 782, p = 0.546; # of *Ppp1r9b* RNPs: CA2 proximal = 8133 ± 1875 and distal = 9502 ± 2208, p = 0.118; CA1 proximal = 6631 ± 1923 and distal= 7279 ± 1955, p = 0.339, paired two-tailed t-tests). We quantified the size distributions of these mRNAs in CA1 and CA2 proximal and distal dendrites and found no detectable differences between proximal and distal dendrites and therefore the data were collapsed and represented as one population per animal. Across animals, we consistently observed the same pattern of size heterogeneity across mRNAs in both hippocampal cell types (Fig. 3DE). However, the distributions of RNP sizes were quite heterogeneous across RNAs; in particular, *Adcy1* displayed (at least) two qualitatively distinct populations of RNP sizes; those measuring less than 0.25 μm^2^ and a population measuring greater than 0.5 μm^2^ (Fig. 3C indicated with red and blue arrows, respectively). Notably, the larger sized *Adcy1* RNP population makes up over 20% of total *Adcy1* RNPs, whereas it makes up less than 10% of total *Ppp1r9b* RNPs and large RNPs are not present for *Rgs14* (Fig. 3DE). When we compared the size distributions, we found that *Adcy1* is significantly different from that of both *Rgs14* and *Ppp1r9b* regardless of the cell type (p<0.0001 for all comparisons, two-tailed unpaired K-S tests, N=4 mice). We interpret the larger sized RNPs to potentially represent RNPs with multiple copies of the same transcript whereas the smaller RNPs may represent RNPs containing fewer copies of transcripts or a single copy.

Subcellular localization of a given RNA is assumed to be affected by the composition of the transport RNP (Mikl et al., 2011; Mitsumori et al., 2017). Thus, we tested whether variable RNP size distribution patterns could reflect distinct RNP compositions that have different combinations and/or amounts of RNAs. In both cell types, *Adcy1* mRNA colocalized with *Ppp1r9b* significantly more than it did with *Rgs14* (*Ppp1r9b* CA2: 22.47 ± 4.99% vs *Rgs14* CA2: 2.93± 1.20%, p = 0.038; *Ppp1r9b* CA1: 21.24 ± 6.17% vs *Rgs14* CA1: 2.38 ± 0.76%, p = 0.047, N=4 mice, paired two-tailed t-tests) (Fig. 3FG). However, the number of *Ppp1r9b* RNPs is almost tenfold higher compared to the number of *Rgs14* RNPs in the hippocampal neuropil. To account for the potential bias of expression differences influencing the extent of colocalization, we rotated the *Adcy1* image 90 degrees and re-calculated the overlapping pixels by chance with either *Ppp1r9b* or *Rgs14*. We found that the chance levels of overlap between *Adcy1* and *Ppp1r9b* were significantly lower than the levels of overlap calculated from the properly registered experimental image in both cell types (Chance CA2: 15.37 ± 4.30, p = 0.003; Chance CA1: 15.18 ± 4.64%, p = 0.016, paired one-tailed t-test, Fig. 3FG). Similar results were found for percent colocalization of *Adcy1* with *Rgs14* in that colocalization values were above chance (Chance CA2: 0.6 ± 0.15% p = 0.06; Chance CA1: 0.41 ± 0.18% p = 0.0405, paired one-tailed t-tests). Interestingly, both *Adcy1* and *Ppp1r9b* are targeted by the same RNA-binding protein, fragile X messenger ribonucleoprotein (FMRP) but *Rgs14* is not (Darnell et al., 2011; Hale et al., 2021). Thus, one interpretation of our finding is that RNAs that are bound by the same RBP show preferential colocalization compared to RNA pairs not bound by the same RBP. This suggests that there may be a biologically efficient form of coregulation for RNAs harboring the same or similar cis sequence motifs.

To test whether the larger *Adcy1* RNPs reflected a unique pool of RNPs with more or less colocalization, we compared the size of *Adcy1* RNPs that colocalized with *Ppp1r9b* in experimental images vs the size of *Adcy1* RNPs that colocalized by chance (Fig. 3H). We found that the size of *Adcy1* RNPs did not influence its’ colocalization with *Ppp1r9b* as demonstrated by the similar size distributions of colocalized *Adcy1* RNPs in experimental vs random images in CA2 (Fig. 3H) and CA1 (data not shown). The larger RNPs colocalized slightly more than the smaller RNPs presumably due to consisting of more pixels with more opportunity to colocalize by chance. Indeed, the average median size of colocalized *Adcy1* RNPs did not differ from chance (CA2: 0.36 ± 0.09 μm^2^ vs. chance = 0.36 ± 0.09 μm^2^, p > 0.05, unpaired two-tailed t test; CA1: 0.34 ± 0.06 μm^2^ vs. chance 0.32 ± 0.07 μm^2^, p >0.05, unpaired two-tailed t test, N=4 mice). Collectively, these data extend our findings to suggest that, in addition to low and multiple copy homomeric RNPs, there are heteromeric RNPs, potentially targeted by the same RBPs, indicative of multiple potential mechanisms regulating RNP composition in dendrites.

### Putative FMRP-target dendritic RNAs show heterogeneous size distributions

**Fig 4.**
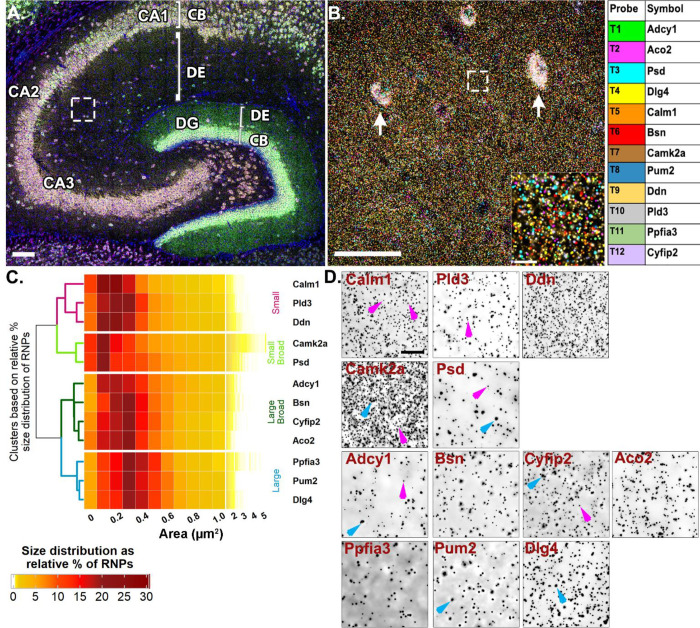
Highly multiplexed RNA imaging reveals dendritic RNAs have variable size distributions **A.** Representative image of mouse hippocampus with *Adcy1, Aco2, Psd, Dlg4* labeling in round 1 of HiPlex smFISH. White box represents ROI from CA2 dendrites. **B.** Representative high-magnification merged image of 12 mRNAs from CA2 dendrites. Arrows denote interneuron expression in the neuropil layer that is removed before analysis (see methods) and inset is the dashed white box. Each mRNA is colored based on the table on the right. **C.** Heatmap of RNP size distributions hierarchically clustered by similarity. **D.** Representative images of each RNA channel. Magenta arrows denote small RNPs in *Calm1*, *Pld3*, *Camk2a*, *Psd*, *Adcy1* and *Cyfip2*. Blue arrows denote larger sized RNPs in *Camk2a*, *Adcy1*, *Cyfip2*, *Ppfia3* and *Dlg4*. Scale = A. 100μm, B. 50μm, 10μm, D. 10μm

Inspired by the observation of homomeric RNA cargoes as well as preferential colocalization between RNAs targeted by the same RBP (heteromeric RNPs), our next experiments were done to 1) extend our findings on dendritic RNP sizes and compositions to more RNAs and 2) to test whether the patterns of colocalization vary when many RNAs are probed at once in an intact neural circuit. Particularly, we wanted to test whether RNAs that are more likely to colocalize based on their shared RBP cis motif(s) show any selectivity in how they associate with other RNAs based on their encoded protein functions or targeted subcellular destination. We took advantage of the relatively well characterized RBP, FMRP, and its dendritically localized target RNAs to quantitatively map their colocalization at subcellular resolution in hippocampal dendrites. We generated a list of candidate target RNAs by cross-referencing datasets that identified hundreds of putative FMRP target RNAs using HITS-CLIP (high-throughput sequencing of RNA isolated by crosslinking immunoprecipitation) on whole brain (Darnell et al., 2011) and hippocampal CA1 neuropil (Sawicka et al., 2019) with datasets that identified high-confidence hippocampal dendritic RNAs (Farris et al 2019; Cajigas 2012; Ainsley 2014). This list of dendritically localized candidate FMRP target RNAs was further curated based on expression, different encoded protein functions (signaling, cytoskeletal, synaptic plasticity, etc.) and target destinations (mitochondria, cytoplasm, cell membrane, dendritic spine) (Supp. Table 1). To spatially map the association of putative FMRP target RNAs, we probed for 12 RNAs at once and iteratively imaged 4 at a time using Hiplex smFISH followed by FMRP immunostaining (Supp. Fig. 1). Each of the mRNAs were present in CA2 dendrites with varying degrees of abundance (Fig. 4AB, Supp. Fig. 2). Based on the findings from our previous experiments (Fig. 1 and Fig. 3), which indicated the presence of distinctly sized populations of homomeric RNPs within the same RNA species, we first calculated the median sizes (Supp. Table 2) and size distributions of the 12 putative FMRP target RNAs. Unsupervised hierarchical clustering analysis of the size distributions revealed four different patterns (Fig. 4CD). The first cluster is comprised of RNAs with consistently “small” RNPs (*Ddn*, *Pld3* and *Calm1*, dendrogram labeled in magenta, Fig. 4C), whereby, on average, ~55% of RNPs are less than 0.3 μm^2^ (54.57 ± 4.84%, averaged across RNAs from N=4 mice) with the largest relative percent peak (23.69 ± 1.77%) at 0.2 μm^2^. These consistently small RNPs have fewer than 10% RNPs (9.29 ± 1.46%) sized 0.6–1.0 μm^2^ and only 3% (2.50 ± 0.64%) of RNPs larger than 1.0 μm^2^. In contrast, the last cluster is comprised of RNAs with consistently “large” RNPs (*Dlg4*, *Pum2* and *Ppfia3*, labeled in blue, Fig. 4C) whereby, on average, ~50% of the RNPs are 0.3–0.5 μm^2^ (49.80 ± 1.16%) with the largest relative peak (20.33 ± 0.42%) at 0.3 μm^2^. These consistently large RNPs have less than 5% RNPs larger than 1.0 μm^2^ (3.14 ± 0.26%). There are two intermediary clusters, “small broad” (*Camk2a* and *Psd*, labeled in light green, Fig. 4C) and “large broad” (*Adcy1*, *Bsn*, *Aco2* and *Cyfip2*, labeled in dark green, Fig. 4C) that have relatively broader size distributions that segregate with either the “small” or “large clusters”, respectively. The “small broad” cluster (*Camk2a* and *Psd*) shows a very broad distribution with the largest relative peak (21.21 ± 0.72%) equal to or less than 0.2 μm^2^ and a larger population of RNPs sized 0.6–1.0 μm^2^ that accounts for more than 15% (15.89 ± 1.71%). These “small broad” RNAs have the largest fraction of RNPs greater than 1.0 μm^2^ at nearly 10% (9.38 ± 1.34%). The “large broad” cluster (*Adcy1*, *Bsn*, *Aco2* and *Cyfip2*) also shows a broad distribution with the largest relative peak (38.85 ± 1.12%) between 0.2–0.3 μm^2^ and a larger population of RNPs sized 0.6–1.0 μm^2^ that accounts for ~15% (15.31 ± 1.05%). However, these “large broad” RNPs have only ~3% of RNPs larger than 1.0 μm^2^ (2.69 ± 0.60%). Small and large populations are denoted on the representative images with magenta and blue arrows, respectively (Fig. 4D). It is interesting to note that even some of the most abundant dendritic RNAs visualized here contain consistently small populations of RNPs (i.e., *Ddn*, *Calm1*). These data extend our findings from Fig. 3 to show that RNAs, regardless of abundance, vary considerably in RNP sizes, both within a transcript population and across different transcripts. While it is likely that the differently sized RNPs reflect copy number variation, it is not clear whether differences in RNP size reflect functional differences, such as associations with other RNAs and/or their capacity for translation.

### Putative FMRP-targeted dendritic RNAs colocalize based on abundance

**Fig 5.**
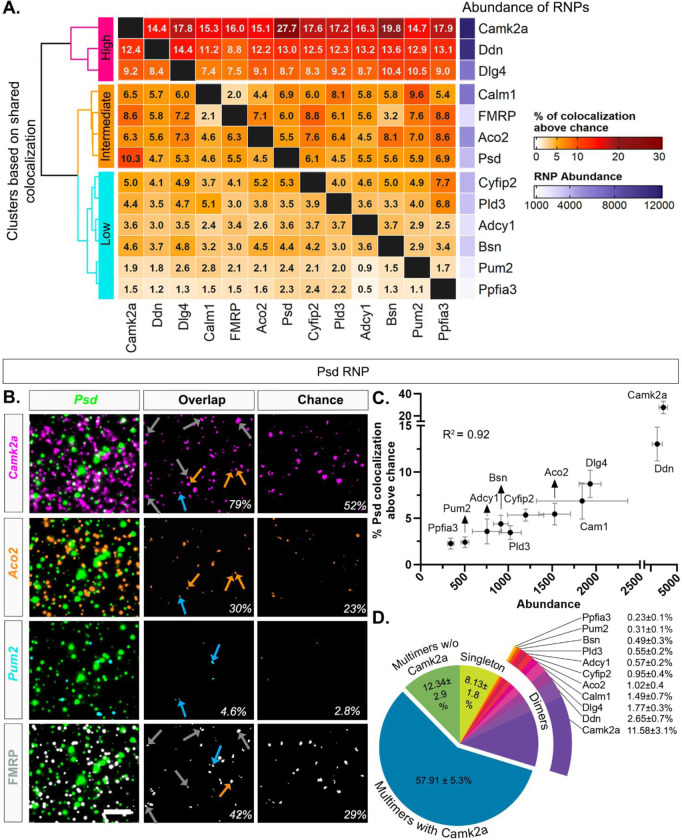
RNP pairwise colocalization patterns correlate with abundance **A.** Percentage of RNPs colocalized with each other above chance in pairwise combinations. Percentage was calculated by dividing the number of overlapping puncta by the total number of RNPs that correspond to each channel. **B.** Representative images of colocalization. The first column consists of merged images of *Psd* (green,) with i) *Camk2a* (magenta), ii) *Aco2* (orange), iii) *Pum2* (cyan) and iv) FMRP (white). The middle column shows the intersecting area of each pair of RNPs *Psd*/ *Camk2a* (magenta,85 out of107 or 79% of *Psd* RNPs colocalize with *Camk2a* in this image), *Psd*/*Aco2* (orange, 33/107, 30%), *Psd*/ *Pum2* (cyan, 5/107, 4.6%) and *Psd*/ FMRP (white,45/107, 42%). The third column shows the intersect of *Psd* by chance (rotated 90 degrees) (*Psd*/ *Camk2a* = 56/107, 52%; *Psd*/ *Aco2* = 25/107, 23%; *Psd*/ *Pum2* = 3/107, 2.8%; *Psd*/ FMRP = 32/107, 29%). **C.** Correlation plot of percent *Psd* colocalized with other 11 RNPs after chance subtraction and their abundance (R^2^ = 0.92, p<0.0001). **D.** Pie chart of *Psd* RNP compositions. The data shown here is from four 512X512 ROIs per animal then averaged across N=4 mice. 91.86 ± 1.77% (vs. 65.1 ± 5.37% chance in Supp. Fig. 5) of *Psd* RNPs have overlapping pixels from all other RNA channels combined, i.e., colocalized RNAs that include dimers (only one other RNA) and multimers (at least two other RNAs). The percentage of colocalized *Psd*-dimers is almost at a level that would be expected by chance, except for *Camk2a*, where the colocalization is significantly higher. This is shown by 0.23 ± 0.1% of *Psd-Ppfia3* dimers (vs. chance 0.23 ± 0.1%) compared to 11.58 ± 3.1% of *Psd-Camk2a* dimers (vs. chance 6.68 ± 1.1%). 57 ± 5.3% of *Psd* RNPs are multimers that have *Camk2a* and at least one other colocalized RNA which is considerably higher than chance (34.89 ± 4.9%). 12.34 ± 2.9% of *Psd* RNPs are also multimers (vs. chance 8.29 ± 1.6%) but do not have *Camk2a*. Lastly, 8.13 ± 1.8% of *Psd* RNPs are not colocalized with any of the other RNAs in our dataset that is noticeably lower than expected by chance (34.9 ± 5.4%). Scale: B. 5μm

To systematically characterize whether any particular FMRP target RNAs display similar colocalization profiles (and thus suggestive of co-regulation), we measured overlapping pixels across RNA channels to determine pairwise colocalization values. Pairwise RNA-RNA colocalization percentages were computed by counting the number of overlapping puncta in between two channels of interest and expressed as a percentage separately for each RNA of the pair. The degree of colocalization across pairs in properly registered experimental images ranged from 4.21 ± 0.48% (*Ppfia3*/*Adcy1*) to 71.38 ± 4.91% (*Camk2a*/*Ppfia3*) (Supp. Fig. 3A). The degree of colocalization that would be expected by chance (one image from every pair rotated 90 degrees, which controls for the expression of both RNAs in the pair) ranged from 3.07 ± 0.41% (*Aco2*/*Ppfia3*) to 53.74 ± 4.75% (*Pum2*/*Camk2a*) (Supp. Fig. 3B). We then subtracted the chance colocalization percentage from the percentage obtained from the properly registered experimental images, anticipating that chance colocalization subtraction would eliminate the relationship with abundance, and visualized the result as a heatmap (Fig 5A). The range of colocalization percentages above chance spanned from 1.11 ± 1.28% to 27.74 ± 5.70%. Thus, after chance correction some RNAs were rarely colocalized whereas others showed ~20–30 times more colocalization suggesting a difference in the propensity of RNA species to be colocalized. We hierarchically clustered the shared colocalization patterns which revealed three distinct clusters displaying consistently “high”, “intermediate”, or “low” levels of colocalization across all pairwise comparisons. Unexpectedly, levels of colocalization increased with RNA abundance such that the three most abundant dendritic RNAs in our dataset, *Camk2a*, *Ddn* and *Dlg4* (# of RNPs: *Camk2a*= 12,829 ± 1,646; *Ddn*= 11,114 ± 1,262, *Dlg4* = 6,426 ± 424, N=4 mice, Supp Fig. 2) exhibited uniformly high colocalization patterns with each of the other RNAs. RNAs with intermediate levels of abundance (*Calm1*= 6,451 ± 2,096, *Aco2* = 5,054 ± 450, *Psd* = 3,648 ± 764; N=4 mice, Supp. Fig. 2) consistently demonstrated intermediary levels of colocalization across all pairwise comparisons. RNAs with relatively lower levels of abundance (*Pld3* = 3,457 ± 427, *Cyfip2* = 3,919 ± 725, *Adcy1* = 2,327 ± 407, *Bsn* = 3,157 ± 450, *Pum2* = 1,694 ± 208, *Ppfia3* = 1,162 ± 178) typically showed lower levels of colocalization across all pairwise comparisons. Representative images of high (*Camk2a*), intermediate (*Aco2*) and low (*Pum2*) levels of colocalization with *Psd* RNP are shown in Fig. 5B, including the intersecting pixel overlaps for properly registered experimental and rotated/chance images. We chose *Psd* as an example RNA due to its intermediate level of abundance, high percent colocalization with *Camk2a,* and its broad size distribution reflective of multiple populations of homomeric clusters.

To visualize the influence of RNP abundance on colocalization, we plotted the percent *Psd* colocalization above chance versus abundance in a correlation plot (Fig. 5C). We found that the abundance of other RNAs is highly correlated with the percent *Psd* colocalization (R^2^= 0.92). We observed the same pattern regardless of the expression of the RNA that is being colocalized (Supp. Fig. 4, *Ddn* (high) & *Pum2* (low). As pairwise colocalization cannot portray the scale of heteromeric RNP compositions, we then quantified the percentage of *Psd* RNPs that are localized singly or in association with at least one (dimers) or more species of RNAs (multimers) in both properly registered experimental and rotated chance images (Fig. 5D and Supp. Fig. 5). The pie chart of *Psd* RNP compositions shows that only 8.13 ± 1.8% of *Psd* RNPs are not colocalized with any RNA (singleton), which is much lower than would be expected by chance (34.9 ± 5.4%). This suggests that the greater proportion of the *Psd* RNPs are present as heteromeric RNPs, containing different types of mRNA transcripts. These include *Psd*-dimers, which have *Psd* colocalized with only one other type of transcript and *Psd*-multimers which contain more than two different transcripts (Fig. 5D and Supp. Fig. 5). The percentage of *Psd*-dimers range from 0.23 ± 0.1% (*Psd*/*Ppfia3*) to 11.58 ± 3.1% (*Psd*/*Camk2a*) that also mirrors the abundance of the RNAs it colocalized with in properly registered experimental images. However, only the percentage of *Psd/Camk2a* dimers (11.58 ± 3.1%) is higher than chance (6.68 ± 1.1%); all other *Psd*-dimers are near chance levels. Importantly, we observed that 70.25 ± 4.8% of *Psd* RNPs have at least three or more (including *Psd*) transcripts (multimers) of which the greatest fraction has *Camk2a* (57.91 ± 5.3%). Only 12.34 ± 2.9% of multimeric *Psd* RNPs were without *Camk2a*, underscoring the dominating presence of *Camk2a* in both *Psd* dimers and multimers in our data. In contrast, the percentage of *Psd* multimers with (34.89 ± 4.9%) and without *Camk2a* (8.29 ± 1.6%) are noticeably lower in the chance rotated images.

Furthermore, when other RNAs were quantified similarly, we found that an average of 88.6 ± 0.73% of each dendritic RNA is localized with at least one other RNA in our dataset compared to 65.62 ± 0.66% expected by chance (all 12 comparisons are significantly higher than chance, unpaired two sample welch’s t-test with FDR correction) (Supp. Fig. 6). Thus, only ~11.40 ± 0.73% of the dendritic RNAs are not colocalized or more likely associated with other RNAs not visualized here. Combined together, our HiPlex data demonstrate that 1) RNP size heterogeneity likely reflects dendritic RNPs composed of varying amounts of the same RNA and 2) the vast majority of RNPs are localized as multiple different species of RNAs (heteromeric RNPs) that scale with RNA abundance, and this effect stands significant despite being corrected for colocalization that would occur by chance.

## DISCUSSION

In this study, we examined the variety of flavors of neuronal RNPs and their co-assemblies in intact mouse hippocampus using high resolution multiplexed smFISH. First, we provide evidence supporting the substantial diversity of dendritic RNP compositions that encompasses varying amounts of a single RNA species and/or a combination of multiple different types of RNA species. Second, our findings suggest that dendritic RNAs targeted by the same trans-acting element, FMRP, tend to colocalize more than they do with an RNA not targeted by FMRP. Third, by simultaneously visualizing a dozen putative FMRP target mRNAs in an attempt to uncover whether transcripts selectively colocalize more or less based on any discernible molecular logic (molecular function or targeted location), we found that every RNA, regardless of its abundance, colocalizes more with highly abundant RNAs compared to RNAs lower in abundance. This result stands after correcting for the colocalization expected by chance of the two RNAs being compared. Lastly, we show limited evidence that dendritic RNPs are composed of only one type of RNA species, suggesting RNAs are co-transported to dendrites. Taken together, RNA abundance may be one of the crucial drivers of heteromeric RNA cargo assembly; a model that could potentially satisfy how neurons maneuver trafficking thousands of transcripts over long distances to maintain and modify neuronal communication.

### Dendritic RNPs contain varying amounts of a single RNA species

Neurons require the localization of thousands of different types of RNAs of variable abundance and subcellular distributions to support synaptic function. Yet, few studies have systematically characterized how dendritic RNAs are sorted into RNPs and how their compositions could support the delivery of thousands of RNAs encoding proteins involved in many different biological processes. Mikl and colleagues (Mikl et al., 2011) investigated the localization of *Map2* and *Camk2a* RNAs in hippocampal neurons in culture, showing that these mRNAs are present in dendrites in distinct RNPs, each containing as few as one or only a few copies of the same transcript with minimal colocalization between the two transcripts. Another study by Batish and colleagues (Batish et al., 2012), visualized pairwise combinations of eight dendritically localized transcripts with smFISH in hippocampal cultured neurons, also showing unimodal distribution of RNA puncta fluorescence intensities and ~4% of colocalization between pairs of RNAs, suggesting that mRNA molecules are trafficked singly and independently of others in neurons. In addition, there is evidence from in situ studies assessing individual RNA content that supports the idea that RNPs localize in variable copy number states. Single molecule FISH detected 𝛃-actin mRNA in hippocampal cultured neurons showed that RNPs may contain multiple copies of 𝛃-actin mRNA and the copy number decreased with increasing distance from the cell soma (Park et al., 2014). Variations in size and intensity of individual *Camk2a*, *Arc* and neurogranin (*Ng)* RNPs in developing neurons were also reported by Gao et al. (Gao et al., 2008). Further, a recent study by Donlin-Asp and colleagues (Donlin-Asp et al., 2021) used molecular beacons in cultured neurons to individually track endogenous mRNAs, *Camk2a* and *Psd95*, and, in addition to detecting single mRNA transport events, they observed mRNAs in heterogeneous copy number states. While extremely informative, most of these studies were done in primary neuronal cultures and limited in the number of species of dendritic RNAs investigated, demonstrating a need to evaluate RNP copy number and composition for the growing list of dendritically localized RNAs in intact neuronal circuits.

Our data, obtained using single- and multiplexed-smFISH to measure RNP sizes in three different cell types in the intact rat and mouse hippocampus (DG, CA1, CA2), corroborate previous observations in culture that RNP content varies from low-copy number RNAs to higher order homomeric RNA clusters of the same transcript (multiple copies of the mRNA derived from the same gene). While we cannot exclude the possibility that the differences in RNP sizes could somehow arise from the multiple labeling or imaging techniques, we did not observe any particular round of imaging or fluorophore wavelength to behave in a certain way that would explain the observation. Further, it is difficult to imagine a technical explanation for observed differences within the same transcript species, unless there is a biological mechanism restricting access to specific populations of transcripts that is impenetrable to protease digestion. Observations from non-neuronal systems, such as drosophila RNA germ granules (Niepielko et al., 2018; Trcek et al., 2020) also indicate that localized RNAs sort into homotypic clusters. However, with the limited number of dendritically localized RNAs visualized for RNP composition, it is not yet clear whether the existence of distinct copy number states is a transcript-specific feature or a transcriptome-wide phenomenon. Our evidence of heterogeneous copy number RNPs within and across 15 dendritic RNAs is consistent with the idea that multiple RNP assembly states coexist for localizing RNPs, potentially providing flexibility to respond to a diverse range of synaptic inputs/ signals. RNA constituents of transporting or localized RNA granules identified by synaptoneurosome- or brain lysate-fractionation are present in monosomes as well as in translationally silent stalled polysomes (Hafner et al., 2019; Kanai et al., 2004; Krichevsky & Kosik, 2001). Therefore, whether different sized RNPs reflect their association with ribosomes or translational status is yet to be determined. Work on other types of cytoplasmic granules, p-bodies and stress granules, show that granule size correlates with increased granule stability (Moon et al., 2019). Further studies are needed to identify whether differences in RNP size and composition reflect different structural properties and/or functional RNP states.

### RNAs targeted by same RBP preferentially colocalize

Previous work in cultured neurons showed that RNAs (*Camk2a*, *Arc* and *Ng*) with similar cis-acting A2RE sequences were targeted by the same RBP, hnRNPA2, which was necessary and sufficient to mediate their individual transport to dendrites. When microinjected at similar levels, these RNAs displayed a high degree of colocalization, indicating they were coassembled in the same RNP. However, the endogenously detected RNAs, which had different levels of expression, showed a greater variability in colocalization, albeit greater than a non-A2RE target RNA (𝛃-actin), suggesting potential differences in RNP copy number influences RNP composition for localized RNAs targeted by the same RBP (Gao et al., 2008). Observation in yeast cells using both transcript-specific RNA pulldowns and smFISH showed that RNAs that encode proteins functioning in a common biological pathway are multiplexed into the same RNP complexes (Gerber et al., 2004; Nair et al., 2021). Consistent with these studies, our data visualizing three G-protein signaling dendritic RNAs also show that RNAs targeted by the same RBP (FMRP targets *Adcy1* and *Ppp1r9b*) demonstrated a bias for colocalization compared to an RNA that is not a demonstrated target of FMRP (*Rgs14*). However, we cannot exclude the fact that lower colocalization of *Adcy1*/ *Rgs14* could also be driven by lower *Rgs14* abundance in CA2 and CA1 compared to *Ppp1r9b*, as would be predicted by our HiPlex results. Further experiments using RNAs with comparable abundance and with and without similar FMRP binding motifs are needed to strengthen our interpretation.

### Majority of dendritic RNPs colocalize with multiple species of RNAs

Observations in previous smFISH studies have reported conflicting conclusions on mRNA colocalization in dendrites (Batish et al., 2012; Gao et al., 2008; Mikl et al., 2011; Tübing et al., 2010). The percentage of colocalized RNA molecules in pairwise combinations ranged from 0.33 – 3.36% (Batish et al., 2012), 5.7 – 8.3% (Mikl et al., 2011), 7 – 32% (Farris et al., 2014) depending on the quantitative definition of colocalization, analysis technique, as well as the RNA combinations that were compared. However, it is unclear 1) whether these observations took into account the colocalization of transcripts that would occur by chance and 2) whether these interpretations (i.e predominantly single RNA transport) would remain valid if more than a few molecules were visualized at once. Our HiPlex data shows pairwise colocalization of twelve RNAs, calculated as percentage of each RNA of interest, ranged from ~1% to ~30% over chance. Since pairwise colocalization is not sufficient to map the extent of multi-transcript RNP composition, we then quantified the percentage of a given RNA that localizes with any of the other eleven RNAs. We found that ~90% (~65% chance) of any RNA species are colocalized with others indicating co-assembly and not singly trafficked as the predominant form of localization. When we looked at the RNP composition of a particular RNA (*Psd*) with 11 other dendritic RNAs, we found that the percentage of singletons was much lower than predicted by chance (~8% vs ~34%). Further, the percentage of multimers was considerably higher than chance (~57% vs ~34%), consistent with our interpretation that a larger fraction of dendritically localized RNPs consist of multiple RNAs rather than single RNAs. It is noteworthy to mention that the majority of *Psd*-dimers were at the level of chance, suggesting it is unlikely for these RNAs to be exclusively co-packaged within the same RNP in the absence of other RNAs. RNPs containing multiple types of transcripts can possibly indicate a mechanism of transport that prefers efficiency over precision. There are multiple lines of evidence that show FMRP targets are differentially altered in the absence of FMRP at the level of RNA localization (Dictenberg et al., 2008; Miyashiro et al., 2003; Steward et al., 1998). It will be interesting for future studies to assess whether distinct RNP identity and compositions make the cargoes more or less vulnerable to FMRP loss. Moreover, there is compelling evidence of FMRP granule remodeling after synaptic activity to support local protein synthesis (Kharod et al., 2023; Sidorov et al., 2013). If the majority of RNPs are assemblies of many different types of RNA transcripts, how selectivity and specificity is achieved to supply nascent synaptic proteomes needs further investigation. *Camk2a* mRNA has been shown to interact with multiple other RBPs in addition to FMRP (RNG105, CPEB, and Staufen) (Moon et al., 2019; Ortiz et al., 2017; Shiina et al., 2005; L. Wu et al., 1998). In our data, *Camk2a* was also present in the highest percentage of heteromeric (both dimeric and multimeric) RNPs. It seems reasonable to hypothesize that *Camk2a*-containing heteromeric RNPs might show some degree of selectivity that is dependent upon which RBP(s) is present in the RNP.

### RNP composition tracks with RNA abundance

The composition of certain types of specialized RNA granules (e.g. stress granules, germ granules ) is demonstrated to be influenced by mRNA abundance/ accumulation (Bauer et al., 2022; Trcek et al., 2020; Van Treeck et al., 2018). Data on neuronal transport RNPs, however, in the context of being influenced by RNA abundance is comparatively limited. Biophysical studies provide evidence that RNA concentration, in addition to RNA structure and stability, favors in vitro RNA-Protein condensate formation (Boeynaems et al., 2019; Garcia-Jove Navarro et al., 2019; Roden & Gladfelter, 2021). Experiments in *Drosophila* germ cell granules (non-neuronal) show that highly abundant RNAs have higher seeding events to initiate homomeric RNP formations through self-recruitment and subsequently recruit other mRNAs to the RNPs (Niepielko et al., 2018). Our pairwise colocalization data, using p17 mouse brain, shows that highly abundant RNAs (*Camk2a, Ddn, Dlg4*) are colocalized the most with the other RNAs indicating availability of transcripts can possibly dominate RNA co-packaging into heteromeric dendritic RNPs. Consistent with our data, Wang & colleagues visualized 950 RNA transcripts in DIV 18 neuronal dendrites using barcode based labeling (Multiplexed Error-Robust Fluorescence In Situ Hybridization- a barcode-based imaging method- MERFISH), expansion microscopy, and spatial proximity clustering and showed that dendritic RNAs with high abundance spatially cluster together (*Camk2a, Ddn, Dlg4, Ppp1r9b, Shank1, Palm*) (Wang et al., 2020). As observed in germ granules, it is plausible to hypothesize that highly abundant dendritic RNAs may have greater advantage of being present in large number of small RNPs that then assemble into larger RNA transport granules (as found by Krichevsky and coworkers) (Krichevsky & Kosik, 2001). We cannot rule out the potential that other RNA-specific features, in addition to their high abundance, may also contribute to the observed colocalization. For example, *Camk2a, Ddn*, *Dlg4* and *Ppp1r9b* transcripts, in addition to being highly abundant, are also the mRNAs that are highly translated in the dendrite as shown by their high ribosomal densities/ associations (Glock et al., 2020). Because studies in neurons and non-neuronal cell types have shown that RNA localization is influenced by several sequence-specific features including, but not limited to, sequence length, RBP-binding motifs, and other cis sequences influencing stability (Lipshitz & Smibert, 2000; Middleton et al., 2019; Wu et al., 2020, Farris et al., 2019), further investigation into the sequence-specific features of the twelve mRNAs in our dataset is needed to determine whether and/or how highly abundant dendritic RNPs sequester other classes of RNPs into the same granule.

In summary, by taking advantage of advanced tools in spatial imaging, the results of this study provide evidence for dendritic RNPs in intact neural circuits having heterogeneous copy number states and diverse RNA combinations along with suggesting a potential role of highly abundant mRNAs in driving the assembly of RNA packages for localization. Subsequent studies should focus on elucidating how different flavors of RNPs functionally influence the localization and translation of messages at synapses.

## Limitations

Our definition of colocalization is based on 2D overlapping in situ signals, which may over- or under-estimate the true level of colocalization. Thus, we cannot exclude the possibility that our techniques are failing to detect every instance of colocalization or detecting false positives due to the limits of our x-y resolution (250 nm). Therefore, additional super-resolution techniques (i.e. STORM) are required to prove whether any RNAs investigated in this study are physically interacting (<250 nm) within an RNP. However, similar to what we report here using isoform specific probes targeting *Shank2*, our previous work showed that probes targeting different regions of *Arc* RNA (*Arc* 5’ and 3’ UTRs) colocalized ~ 30% in rat hippocampal neuropil (Farris et al 2014). Colocalization in both instances was less than 100% likely due to the different efficiencies of the probes to bind, e.g. 5’UTR has more structure and detected fewer RNPs compared to the probe targeting the 3’UTR. However, there was a high degree of colocalization when more than one RNA transcript was present, i.e., in transcriptional foci in the nucleus where 100% colabeling of 5’ and 3’ probes was detected. Thus, we reason that this technique (RNAscope/HiPlex) is more limited in its ability to detect co-labeling of the same individual RNA transcript with two probes (~30%), perhaps due to steric hindrance or competition of the DNA-based labeling approach, but it is highly likely to detect colocalization when more than one transcript is being labeled (e.g. two transcripts of the same RNA or two distinct dendritically localized RNAs).

## METHODS

### Animals

Sexually naive adult female Sprague Dawley rats were used for the Arc dilution studies in Fig. 1. Both male and female C57BL/6J were used at 8–16 weeks of age for Fig. 2 & 3 and at p17 (post-natal day 17) age for Fig. 4 & 5. Animals were group-housed under a 12:12 light/dark cycle with access to food and water ad libitum. All procedures were approved by the Animal Care and Use Committee of Virginia Tech or University of California Irvine and were in accordance with the National Institutes of Health guidelines for care and use of animals.

### Stimulation Paradigm

The stimulation paradigm used in Fig. 1 was as previously described (Steward and Worley, 2001) with the following modifications. Briefly, an electroconvulsive seizure (ECS) was induced in unanesthetized adult female Sprague Dawley rats by delivering AC current (60Hz, 40mA for 0.5s). Anesthesia was induced immediately after ECS by i.p. injection of 20% urethane. The animals were then placed in a stereotaxic apparatus and a stimulating electrode was positioned to selectively activate one side of the medial perforant path projections (1.0 mm anterior to transverse sinus and 4.0 mm lateral from the midline). The electrode depth was empirically determined to obtain a maximal evoked response in the dentate gyrus at a minimal stimulus intensity, typically 3–4mm deep from the cortical surface. A recording electrode was positioned in the molecular layer of the dorsal blade of the dentate gyrus (3.5 mm posterior from bregma, 1.8 mm lateral from the midline, 3–3.5 mm from the cortical surface based on evoked responses generated by stimulation). Single test pulses were then delivered at a rate of 1/10 s for 20 minutes to determine baseline response amplitude. Two hours after the ECS delivery, high frequency stimulation (trains of eight pulses at 400hz) were delivered at a rate of 1/10 s. After 60 minutes the brain was removed and flash frozen. Brains were embedded in OCT and sectioned in the coronal plane on a cryostat at 20 μm and processed for FISH as described below.

### Fluorescent in situ hybridization (FISH)

FISH was performed as previously described (Guzowski et al., 1999; Farris et al 2014) to examine *Arc* mRNA puncta in dendritic fields of the dentate gyrus. For the dilution experiments, a 1X saturating stock of full length dig-labeled *Arc* probe was serially diluted with full length unlabeled *Arc* probe at 1:2 and 1:4.

### Quantitative Analyses of *Arc* puncta number and size

Sections processed for FISH were imaged across the molecular layer of the dentate gyrus at 63X using a confocal microscope. The size and number of *Arc*-positive puncta were determined using imageJ particle analysis function (NIH). Briefly, the images were overlaid using DAPI and a region of interest (ROI) was determined so as to count each *Arc*-puncta only once. The entire image was then set to an unbiased threshold and watershed to segment individual *Arc*-puncta. The ROI was then cropped out of the original image and subjected to particle analysis. The number and feret’s diameter of *Arc*-puncta were averaged across three sections from one animal and data are presented as mean +/− SEM across animals.

### Single Molecule Fluorescence in situ hybridization

Brains were embedded in OCT and sectioned in the horizontal plane (mouse studies) on a cryostat at 20 μm and processed for single molecule FISH according to the RNAscope Fluorescent Multiplex kit instructions (Advanced Cell Diagnostics, Hayward, CA). The following mus musculus specific probes were used with the RNAscope fluorescent multiplex reagent kit (Cat# 320850): Rgs14 (Cat #416651), *Adcy1* (Cat #451241), *Shank2*-Pan (Cat #513711, NM_001113373.3/ENSMUST00000105900.8), *Shank2*-O3 (Cat # 851661-C2, ENSMUST00000146006.2/NM_001113373.3), *Shank2*-O4 (Cat # 852961-C3, ENSMUST00000105900.8/NM_001081370.2), mouse 3 plex positive control (Cat # 320881), 3 plex negative control (Cat # 320871). Mus musculus specific probes used for HiPlex assay includes *Adcy1* (Cat #451241-T1), *Aco2* (Cat #1120581-T2), *Psd* (Cat #449711-T3), *Dlg4* (Cat #462311-T4), *Calm1* (Cat #500461-T5), *Bsn* (Cat #1119681-T6), *Camk2a* (Cat #445231-T7), *Pum2* (Cat #546751-T8), *Ddn* (Cat #546261-T9), *Pld3* (Cat #507241-T10)*, Ppfia3* (Cat #1119691-T11), *Cyfip2* (Cat #561471-T12), HiPlex positive control (Cat #324321) and negative control (Cat #324341).

### smFISH Image Acquisition and Quantification

All images were acquired on a confocal microscope using a 40X oil immersion lens. Acquisition parameters were set using 3plexed negative controls (cDNA probes against bacterial RNAs not present in mouse tissue) in each of the 3 channels (Alexa 488, Atto 550, Atto 647) so that any signal above the level of background was acquired. Area CA2 borders were identified using Pcp4 or Rgs14 as molecular markers; area CA1 was identified using its defined anatomical location. Each image was auto thresholded and particle number was quantified across the entire image (354.25 × 354.25μm) or a square region of interest (ROI, of constant size) using the analyze particle function in Fiji (NIH, v2) (Schindelin et al., 2012). Particle counts per subregion were averaged across sections (typically 2–4 sections per animal) to obtain one value per animal, and data are represented as mean particle count across animals ± SEM. All statistical analyses were carried out using Graphpad PRISM 7 software, and significance was determined using an alpha level of 0.05.

### *Shank2* smFISH, image acquisition, and analysis

smFISH was performed according to the instructions provided in the kit (Cat# 320850). Probes labeling *Shank2e*-long, *Shank2a*-short and pan (both 2a and 2e) were imaged in channels with LEDs Alexa-647, Atto-555 and Atto-488 respectively. ROIs captured from CA2 cell body were 211 μm X 211 μm in x-y plane and 5μm in z (25 steps, step size: 0.21μm) at 63X magnification using a Leica thunder (Leica DMi 8) wide-field fluorescence microscope. Images were denoised and deconvolved in NIS elements AR to increase signal to noise ratio and remove background. After all computational processing steps for signal optimization, maximum projection was used for further analysis. A binary segmentation layer was created using the “bright spot” command in NIS AR based on the fluorescent intensity of the puncta. For overlapping signals across individual channels, additional binary layers were created combining binaries from individual layers. Data was then extracted to excel. Colocalization fraction was calculated as the percentage of overlapping puncta relative to the total number of puncta positive for that individual channel of interest. Example equation is as follows:

$$Percentage\;of\;shank2\;-\;pan\;colocalzing\;with\;any\;5^{'}(\mathrm{shank}2a\;or\;shank2e)=\frac{\;\mathrm{No.}\;\mathrm{of}\;\mathrm{shank}2\;-\;\mathrm{pan}\;\mathrm{puncta}\;\mathrm{that}\;\mathrm{overlaps}\;\mathrm{with}\;\mathrm{any}\;\mathrm{puncta}\;\mathrm{in}\;\mathrm{shank2e}\;\mathrm{or}\;\mathrm{shank2a}\;}{\;\mathrm{Total}\;\mathrm{number}\;\mathrm{of}\;\mathrm{Shank}2\;-\;\mathrm{pan}\;\mathrm{puncta}\;}X100$$

For calculation of pixels that are randomly colocalized (chance), each image in a pair of comparisons was rotated 180 degrees and then the computation was repeated again and presented as ‘chance’ in Fig. 2D.

### Multiplex smFISH, image acquisition, and analysis

Mouse brains were flash frozen in chilled isopentane followed by blocking in OCT. 20μm cryosections were slide mounted for smFISH. smFISH was performed using RNAscope fluorescent multiplex reagent kit as described in the protocol. 211 μm X 211 μm ROI (in x-y plane) of 5μm thickness Z-stack images (25 steps, step size 0.21μm) were acquired by a Leica thunder (Leica DMi 8) wide-field fluorescence microscope at 63X magnification (Numerical aperture 1.4, refractive index 1.51). CA2 was identified using Rgs14 and *Adcy1* labeling. *Rgs14, Adcy1* and *Ppp1r9b* were imaged using 488, 550 and 647nm LED. CA1 and CA2 proximal and distal dendritic regions were imaged from N=4 adult mouse hippocampus. Exported TIFF images were then processed using NIS elements AR. Each image including negative control was computationally processed by denoising and automatic deconvolution algorithm. A binary segmentation layer was created per channel on the post-processing max projection images based on fluorescent intensity and manually edited to best represent the data. Any signal overlapping plus 10% of DAPI was excluded from quantification to confirm only RNAs in the pyramidal neuron dendrites but not in glia or interneurons are included in the analysis. After manually editing each binary layer, size data was exported to excel and plotted with prism.

Size data of *Rgs14, Adcy1, Ppp1r9b* from proximal and distal dendrites of CA2 and CA1 was plotted in prism as individual histograms (size in X-axis and number of RNAs in y-axis) of each animal at first (data not shown). Although, total number of RNA puncta varied for each mRNA species from mouse to mouse, a consistent pattern of size distribution was noted when plotted as a percentage fraction for size distribution. Due to consistent patterns across dendritic laminae, data from proximal and distal dendrites were then combined to represent the size histogram as the relative percentage of RNAs of different sizes per hippocampal region (Fig. 3. D&E). For RNPs of size 0.0 to 0.48μm^2^, bin width is kept at 0.12 and for RNPs of area>0.48μm^2^, bin width is kept as 0.24 as plotted on the X-axis (Fig. 3D & E). Anything equal to or greater than 5 μm^2^ is stacked into the same bin using overflow binning in excel.

Colocalization between individual channels was defined as touching or overlapping binary objects from two separate channels of interest. For object-based colocalization analysis, additional binary layers were created as “*Adcy1* mRNA puncta having any overlapping pixel from *Rgs14*” and “*Adcy1* mRNA puncta having any overlapping pixel from *Ppp1r9b*”. The number of *Adcy1* puncta that colocalized with either *Rgs14* or *Ppp1r9b* was then divided by the total number of *Adcy1* puncta and plotted as a percentage (Fig. 3F&G). To calculate the percentage of randomly overlapping pixels, *Adcy1* image was rotated 90 degrees and the steps of creating an additional binary layer that includes signal overlaps from Rgs14 and Ppp1r9b was repeated, and the percentage was calculated against the total number of *Adcy1* particles.

### Hiplex smFISH, image acquisition and analysis

smFISH was performed on slide mounted 20μm sections using RNAscope HiPlex Assay V2(Cat# 324120). After fixation, samples were dehydrated in % ethanol and treated with protease IV for 30 mins. Samples were hybridized at 40℃ with the twelve probes for 2 hours followed by signal amplification steps (check notes). T1-T4 fluorophores were added to image *Adcy1, Aco2, Psd* and *Dlg4* mRNAs in round one. For each round, 488, 550, 647 and 750 nm LED were used to image four RNAs at 63X magnification (Numerical aperture 1.4, refractive index=1.51). Leica Thunder epifluorescence microscope (Leica DMi 8) was used for imaging with recorded stage positions to acquire the same ROIs across rounds. *Adcy1* mRNA probe was in round one and used as a CA2 marker and DAPI signal was acquired using a 390 nm LED. After round one, coverslips were taken off by keeping slides in 4X SSC, fluorophores were cleaved and FFPE reagent was used to decrease background. Subsequently T5-T8 fluorophores were added to image *Calm1, Bsn, Camk2a* and *Pum2* in the second round. This was followed by similar steps of cleaving the fluorophores and background removal. For the final round, T9-T12 fluorophores were added to image *Ddn, Pld3, Ppfia3, Cyfip2* mRNAs. Exposure was adjusted in each round matching with the expression of individual RNAs but kept consistent across all animals. After completion of smFISH, fluorophores were cleaved and slides were washed in TBS for 2X5mins, blocked in TSA-blocking solution for 30 mins and incubated with anti-rabbit-FMRP primary antibody (1: 100, Abcam, Cat# ab17722, Lot# 632949982) at 4C for two consecutive nights. Subsequently slides were washed in TBS-T (0.05% Tween) for 3X5 mins and 2% H2O2 in TBS for 10 mins at room temperature. Following that, slides were incubated with goat-anti-rabbit HRP (1:250, Jackson Immunoresearch, Cat#111035144, Lot# 149770) for 2 hours at room temperature. Slides were washed in TBS-T before they underwent incubation with brand new TSA-Cy3 kit (1:50, Cat# NEL704A001KT, Lot# 210322048) for 30 mins at room temperature. Slides were then washed in TBS-T for several times and coverslipped with prolong gold antifade mounting medium. Images of FMRP immunostaining were done using tissue from N=2 animals, imaged using 550 LED and signals were adjusted using negative control slide. ROIs from proximal and distal dendrites of CA1 and CA2 were imaged that were 211μm X 211 μm in x-y plane and 5μm in z-plane (step size 0.21μm) at 63X magnification (Numerical aperture 1.4, refractive index 1.51). All z-stack images of individual channels and rounds were exported as TIFF images and converted to nd2 format for further processing on NIS elements AR. Denoising ai and Richardson-Lucy deconvolution algorithm was used to increase the signal to noise ratio and minimize background pixel intensity. Images were then max projected and registered using ACD RNAscope Hiplex image registration software based on the DAPI signal of each round. After registration, a composite image of 12 RNA channels and DAPI was created for segmentation and puncta size and colocalization analysis. Each channel was segmented to a binary layer based on fluorescence intensity threshold and manually edited to best represent the ground truth. Any binary object/ fluorescence signal overlapping DAPI was removed from the analysis. Size data was exported per RNA channel for N=4 mice and then averaged for plotting on the heatmap (Fig. 4C). For colocalization analysis, four 512X512 ROIs were cropped from each registered set of image. For each RNA, subsequent binary layers were created that would contain only RNA puncta having any overlap from each of the other channels/ RNAs in consideration. Thus, eleven binaries were created for each RNA channel to calculate the number of that RNA having pixel overlaps with any of the other eleven RNAs. This number was then expressed as a percentage of the given RNA (Supp Fig. 3A). For the quantification of the chance level of overlaps, the same method of calculation was followed only after rotating the image of the given RNA to 90 degrees right (Supp Fig. 3B). Percentage of colocalization due to chance was subtracted from the percentage of colocalization calculated from experimental images and plotted in a heatmap (Fig. 5A). Calculation of *Psd* percent colocalization with any RNA in HiPlex dataset was done by creating a union layer of all intersect binary layers for *Psd*. Thus “*Psd* having *Camk2a* (RNA 1)”, “*Psd* having *Ddn* (RNA 2)”....”*Psd* having *Ppfia3* (RNA 11)” all layers were merged to create a union layer that includes *Psd* puncta that has pixel overlaps from any of the other 11 channels. This data was then exported for N=4 mice, averaged and plotted with abundance of RNAs in Fig. 5C. Similarly, correlation of RNP colocalization with RNA abundance was calculated for *Ddn* and *Pum2* (Supp. Fig. 4).

### Statistical analyses

All statistical analyses were done using graphpad prism (Graphpad Prism 10) with a significance level of 0.05 or lower (**α**=0.05).

## Supplementary Material

Supplement 1

## MAIN POINTS

- Dendritic RNAs exist as diversely composed RNPs that contain variable quantities of an individual transcript as well as combination of multiple different species of transcripts.
- RNAs targeted by the same RBP display a bias to localize together as opposed to being colocalized with a non-target RNA.
- Dendritically localized RNAs, regardless of their individual abundance, colocalize more with highly abundant RNAs compared to less abundant RNAs, even after correcting for random colocalization based on the abundance of RNAs being compared. These data are consistent with the possibility that RNA abundance plays a role in driving the assembly of heteromeric RNA cargos, facilitating the transport of thousands of transcripts to distal compartments
- There is limited evidence of dendritic RNPs being composed of a single RNA species, suggesting that dendritic RNA cargoes are multimeric complexes *in vivo*.
