# Supplementary material for: Multiplexed smFISH reveals the spatial organization of neuropil localized mRNAs is linked to abundance
Source: bioRxiv. 2025 May 5:2024.07.13.603387. Originally published 2024 Jul 17. Preprint. [Version 4] doi: 10.1101/2024.07.13.603387 (PMC11275876; doi:10.1101/2024.07.13.603387)
Supplement: Supplement 2 [file NIHPP2024.07.13.603387v4-supplement-2.pdf]

bioRxiv preprint doi: https://doi.org/10.1101/2024.07.13.603387; this version posted May 5, 2025. The copyright holder for this preprint (which was not certified by peer review) is the author/funder, who has granted bioRxiv a license to display the preprint in perpetuity. It is made available under aCC-BY 4.0 International license.

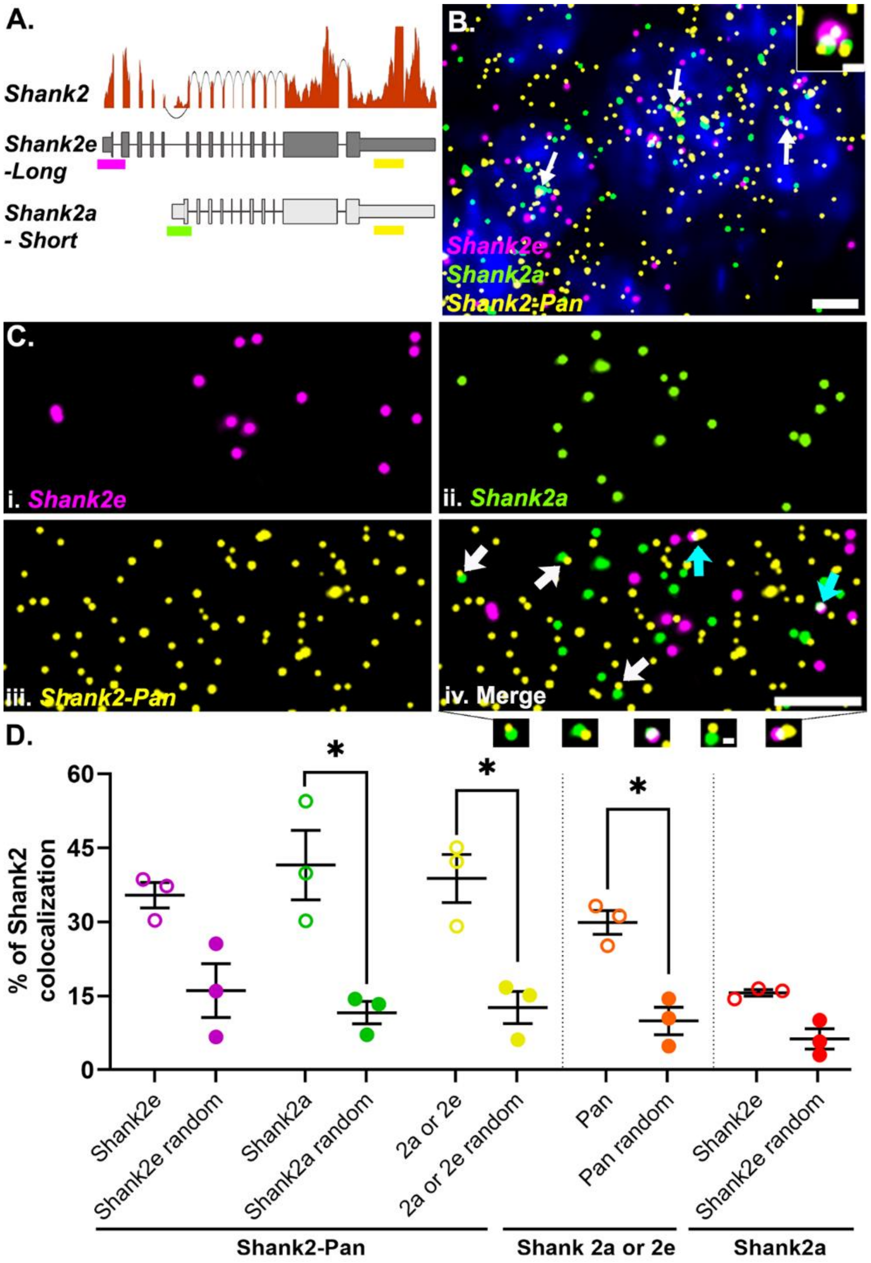

Supplemental Fig. 1. *Shank2* isoform-specific 5' probes are highly colocalized with the Pan 3' probe.

A. *Shank2* isoform gene models with RNAseq read depth data showing the relative expression levels in hippocampal CA2. Sequences from either long (*Shank2e*) or short (*Shank2a*) transcripts targeted by different 5' probes (magenta and green, respectively) and both targeted by the Pan 3' probe (yellow) are shown.

B. Representative image of the three *Shank2* probes in CA2 cell bodies. Nuclei are labeled with DAPI (blue). White arrows indicate example transcriptional foci. Dashed white box is the inset showing a transcriptional focus labeled by all three probes.

C. High-magnification images of (i) *Shank2e*, (ii) *Shank2a*, (iii) *Shank2*-Pan and (iv) the merged image. Arrows indicate example colocalization of *Shank2*-Pan 3' probe with either *Shank2e* 5' probe (cyan arrows) or *Shank2a* 5' probe (white arrows) as shown below.

D. Quantification of the % colocalization between the *Shank2e* (magenta) and *Shank2a* (green) or both (yellow) with the *Shank2*-Pan probe (open circles) compared to that observed by random colocalization (closed circles). The percent of *Shank2*-Pan colocalized with either *Shank2a* or *Shank2e* (orange) compared to random colocalization and the percent of *Shank2e* colocalizing with *Shank2a* (red) compared to random, many of which are transcription foci, as shown in B.
Error bars indicate SEM; N=3 mice; * denotes $p < 0.05$ from paired one-tailed t-test. Scale bars: B) 5 µm, 1 µm, C) 5 µm, 0.5 µm.

bioRxiv preprint doi: https://doi.org/10.1101/2024.07.13.603387; this version posted May 5, 2025. The copyright holder for this preprint (which was not certified by peer review) is the author/funder, who has granted bioRxiv a license to display the preprint in perpetuity. It is made available under aCC-BY 4.0 International license.

Supp. Table 1 (Refers to Fig. 2 and 3): HiPlex probe information. See Supplemental file for full datasheet.

bioRxiv preprint doi: https://doi.org/10.1101/2024.07.13.603387; this version posted May 5, 2025. The copyright holder for this preprint (which was not certified by peer review) is the author/funder, who has granted bioRxiv a license to display the preprint in perpetuity. It is made available under aCC-BY 4.0 International license.

**Probe**	**Gene Symbol**	**NCBI Transcript ID**	**Ensembl ID**	**Uniprot**	**Allen Brain Atlas**	**Protein Name (Symbol)**	**Protein Function**	**Subcellular Localization**	**FMRP CLIP Rank** (Darnell et al., 2011)	**CA2 Neuropil Expression** (Log2) Farris et al 2019
T1	*Adcy1*	NM_001281768.2	ENSMUSG00000020431	https://www.uniprot.org/uniprotkb/O88444/entry	https://mouse.brain-map.org/gene/show/129123	Adenylate cyclase type 1 (ADCY1)	Adenylate cyclase activity, synaptic transmission, G-protein signalling, Post-syaptic density	Cytoplasm	7	16.44
T2	*Aco2*	NM_080633.2	ENSMUSG00000022477	https://www.uniprot.org/uniprotkb/Q99KI0/entry	https://mouse.brain-map.org/experiment/show?id=67978674	Aconitate hydratase, Aconitase mitochondrial (ACO2)	Essential enzyme in the tricarboxylic acid cycle, isocitrate metabolic processes, mitochondrial metabolism	Cytoplasm, mitochondrial matrix	228	14.47
T3	*Psd*	NM_028627.2	ENSMUSG00000037126	https://www.uniprot.org/uniprotkb/Q5DTT2/entry	https://mouse.brain-map.org/gene/show/49569	Pleckstrin homology and SEC7 domain-containing protein 1 (PSD, also known as Exchange factor for ARF6)	Dendritic spine, postsynaptic density, phospholipid binding	Dendritic spine	297	13.76
T4	*Dlg4*	NM_007864.3	ENSMUSG00000020886	https://www.uniprot.org/uniprotkb/Q62108/entry	https://mouse.brain-map.org/gene/show/13164	Disks large homolog 4 (DLG4, also known as PSD95)	Postsynaptic scaffolding protein, synaptic plasticity, postsynaptic density	Dendritic spine	52	15.25
T5	*Calm1*	NM_001313934.1	ENSMUSG00000001175	https://www.uniprot.org/uniprotkb/P0DP26/entry	https://mouse.brain-map.org/gene/show/12098	Calmodulin-1 (CALM1)	Calcium signal transduction pathway	Cytoplasm	453	16.09
T6	*Bsn*	NM_007567.2	ENSMUSG00000032589	https://www.uniprot.org/uniprotkb/O88737/entry	https://mouse.brain-map.org/gene/show/12003	Bassoon (BSN)	Presynaptic scaffolding protein, protein localization to synapse	Axonal cytoplasm	1	15.4
T7	*Camk2a*	NM_009792.3	ENSMUSG00000024617	https://www.uniprot.org/uniprotkb/P11798/entry	https://mouse.brain-map.org/gene/show/12107	Calcium/calmodulin-dependent protein kinase type II subunit alpha (CAMKIIa)	Calmodulin-binding, synaptic plasticity, dendritic spine development, post-synaptic density	Dendritic Spine	39	18.87
T8	*Pum2*	NM_001160219.1	ENSMUSG00000020594	https://www.uniprot.org/uniprotkb/Q80U58/entry	https://mouse.brain-map.org/experiment/show?id=68845514	Pumilio homolog 2 (PUM2)	Cytosolic RNA-binding protein, translation regulation	Cytoplasm	398	13.35
T9	*Ddn*	NM_001013741.1	ENSMUSG00000059213	https://www.uniprot.org/uniprotkb/Q80TS7/entry	https://mouse.brain-map.org/experiment/show?id=71212512	Dendrin (DDN)	Enables RNA polymerase II cis-regulatory region sequence-specific DNA binding activity	Cytoplasm, dendritic spine membrane	180	16.32
T10	*Pld3*	NM_001317355.2	ENSMUSG00000003363	https://www.uniprot.org/uniprotkb/O35405/entry	https://mouse.brain-map.org/experiment/show?id=77464848	Phospholipase D3 (PLD3)	Lysosomal protein, phospholipase activity,	Lysosomal membrane, endoplasmic reticulum membrane	537	13.31
T11	*Ppfia3*	NM_029741.2	ENSMUSG00000003863	https://www.uniprot.org/uniprotkb/P60469/entry	https://mouse.brain-map.org/experiment/show?id=69202693	Liprin-alpha-3 (PTPRF-interacting protein alpha-3, PPFIA3)	Synaptic vesicle docking, presynaptic active zone cytoplasmic component	Cytoplasm	736	12.26
T12	*Cyfip2*	NM_001252459.1	ENSMUSG00000020340	https://www.uniprot.org/uniprotkb/Q5SQX6/entry	https://mouse.brain-map.org/experiment/show?id=74357791	Cytoplasmic FMR1-interacting protein 2 (CYFIP2)	Actin filament reorganization, neuronal projection development	Cytoplasm	9	15.13

bioRxiv preprint doi: https://doi.org/10.1101/2024.07.13.603387; this version posted May 5, 2025. The copyright holder for this preprint (which was not certified by peer review) is the author/funder, who has granted bioRxiv a license to display the preprint in perpetuity. It is made available under aCC-BY 4.0 International license.

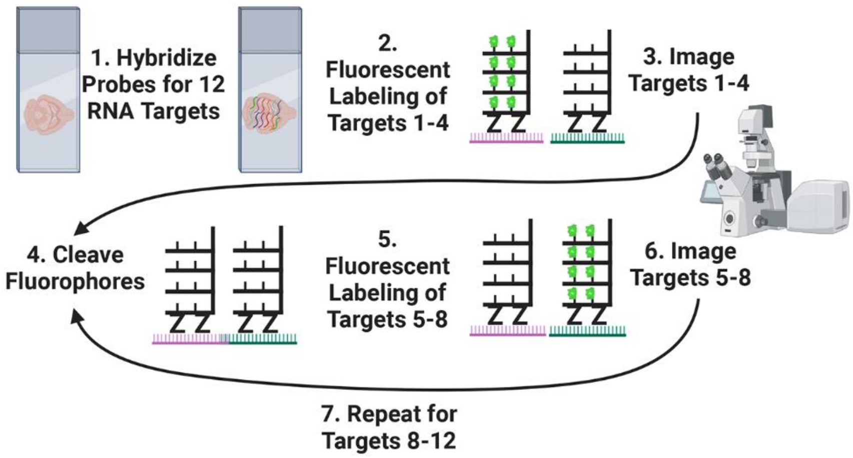

Supplemental Fig. 2 (Refers to Fig. 2 and 3): Schematic showing workflow of HiPlex smFISH.

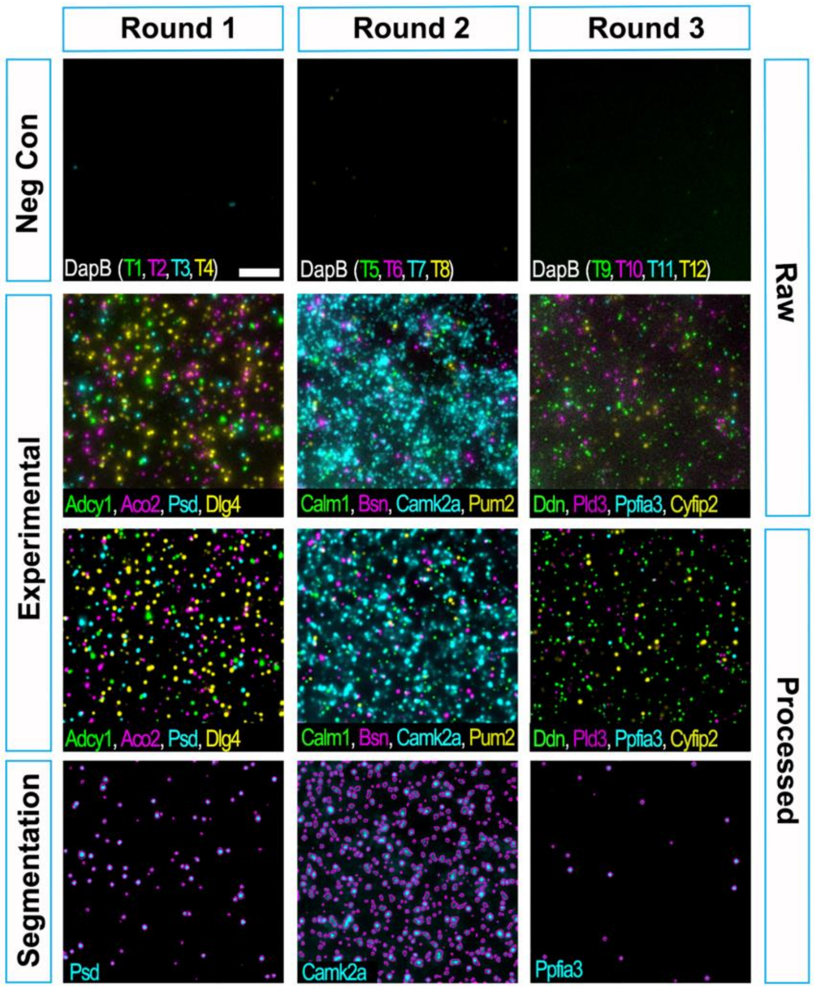

Supplemental Fig. 3 (Refers to Figs. 2-4): HiPlex image processing and segmentation. Raw and processed negative control images probed for the bacterial RNA *DapB* in each channel. Negative control images were acquired with identical acquisition parameters as experimental images shown below from all three rounds of HiPlex smFISH. Experimental images are presented with the same intensity thresholds as the corresponding negative control channels. The last row displays segmented binary layers for the *Psd, Camk2a*, and *Ppfia3* channels, created using intensity thresholds determined from the negative control image of the corresponding channels in each round. Scale: 5 µm.

bioRxiv preprint doi: https://doi.org/10.1101/2024.07.13.603387; this version posted May 5, 2025. The copyright holder for this preprint (which was not certified by peer review) is the author/funder, who has granted bioRxiv a license to display the preprint in perpetuity. It is made available under aCC-BY 4.0 International license.

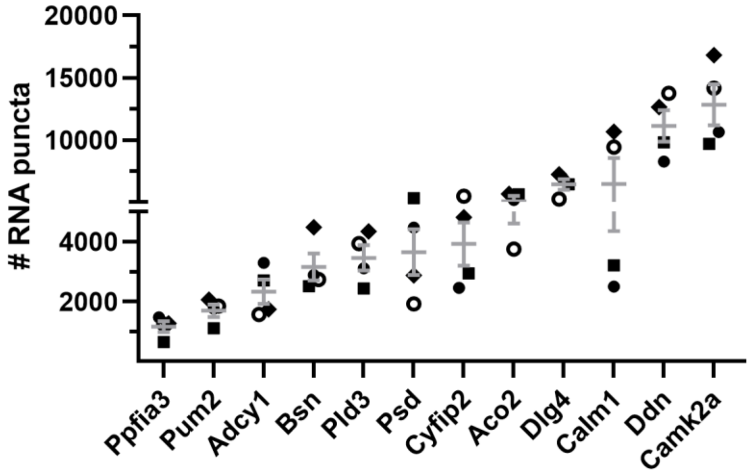

Supplemental Fig. 4 (Refers to Fig. 2 and 3): Abundance of each mRNA in the CA2 neuropil. Each symbol represents data from a biological replicate (N=4 mice). Error bars indicates SEM.

Supplemental Table 2: Average median size of twelve mRNAs visualized in HiPlex smFISH (N=4 mice).

**RNA**	**Average median puncta area (μm^2)**
Calm1	0.20 ± 0.03
Ddn	0.24 ± 0.02
Pld3	0.25 ± 0.03
Camk2a	0.24 ± 0.02
Psd	0.30 ± 0.05
Adcy1	0.25 ± 0.01
Bsn	0.32 ± 0.03
Cyfip2	0.29 ± 0.02
Aco2	0.28 ± 0.01
Ppfia3	0.34 ± 0.01
Pum2	0.34 ± 0.02
Dlg4	0.36 ± 0.02

bioRxiv preprint doi: https://doi.org/10.1101/2024.07.13.603387; this version posted May 5, 2025. The copyright holder for this preprint (which was not certified by peer review) is the author/funder, who has granted bioRxiv a license to display the preprint in perpetuity. It is made available under aCC-BY 4.0 International license.

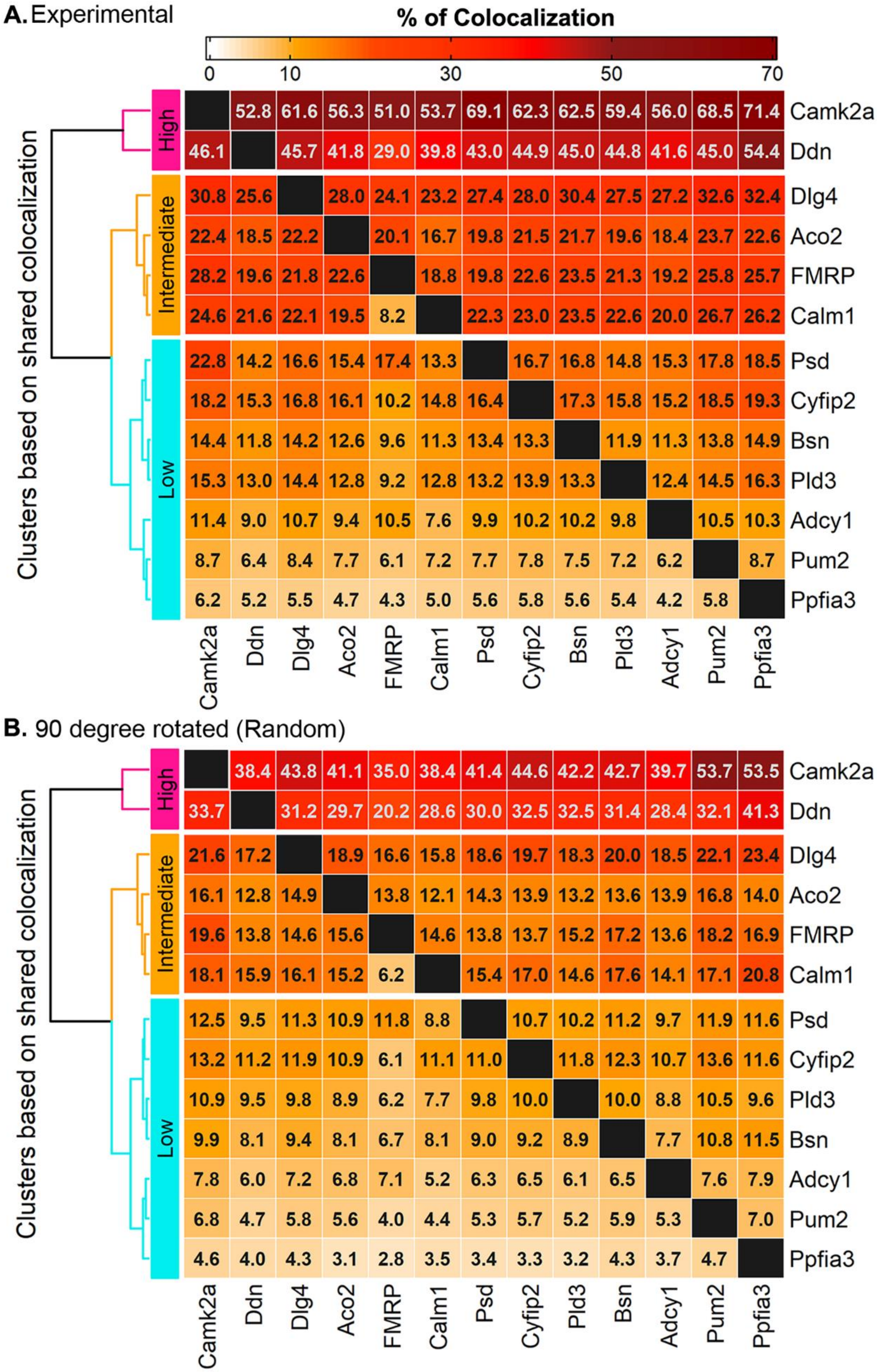

Supplemental Fig. 5 (Refers to Fig. 3A): Heatmaps showing the total average pairwise colocalization of mRNAs in properly registered (experimental) images **(A)** and in rotated (random) images **(B)**. The percentage values in each column are calculated by dividing the number of column mRNAs colocalizing with each row mRNA by the total number of column mRNAs, i.e. 5.2% of *Ddn* colocalizes with *Ppfia3*, whereas 54.4% of *Ppfia3* colocalizes with *Ddn* before random colocalization subtraction (see Fig. 4A). Values are the average of N=4 mice (four 52 X 52 μm^2 images averaged per mouse).

bioRxiv preprint doi: https://doi.org/10.1101/2024.07.13.603387; this version posted May 5, 2025. The copyright holder for this preprint (which was not certified by peer review) is the author/funder, who has granted bioRxiv a license to display the preprint in perpetuity. It is made available under aCC-BY 4.0 International license.

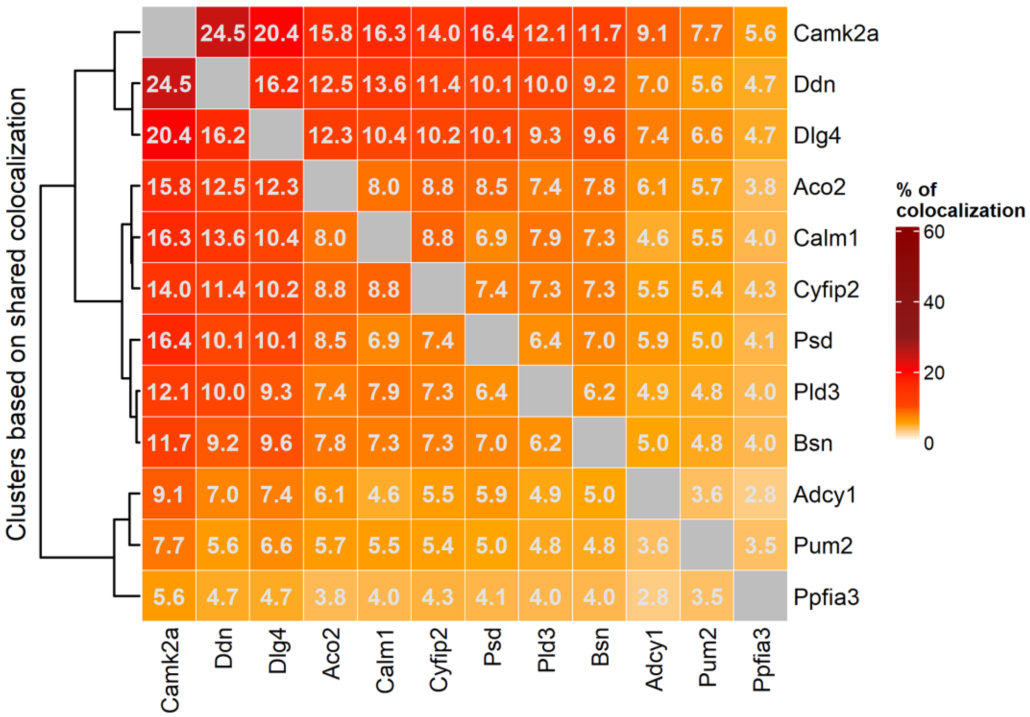

	Camk2a	Ddn	Dlg4	Aco2	Calm1	Cyfip2	Psd	Pld3	Bsn	Adcy1	Pum2	Ppfia3
Camk2a		24.5	20.4	15.8	16.3	14.0	16.4	12.1	11.7	9.1	7.7	5.6
Ddn	24.5		16.2	12.5	13.6	11.4	10.1	10.0	9.2	7.0	5.6	4.7
Dlg4	20.4	16.2		12.3	10.4	10.2	10.1	9.3	9.6	7.4	6.6	4.7
Aco2	15.8	12.5	12.3		8.0	8.8	8.5	7.4	7.8	6.1	5.7	3.8
Calm1	16.3	13.6	10.4	8.0		8.8	6.9	7.9	7.3	4.6	5.5	4.0
Cyfip2	14.0	11.4	10.2	8.8	8.8		7.4	7.3	7.3	5.5	5.4	4.3
Psd	16.4	10.1	10.1	8.5	6.9	7.4		6.4	7.0	5.9	5.0	4.1
Pld3	12.1	10.0	9.3	7.4	7.9	7.3	6.4		6.2	4.9	4.8	4.0
Bsn	11.7	9.2	9.6	7.8	7.3	7.3	7.0	6.2		5.0	4.8	4.0
Adcy1	9.1	7.0	7.4	6.1	4.6	5.5	5.9	4.9	5.0		3.6	2.8
Pum2	7.7	5.6	6.6	5.7	5.5	5.4	5.0	4.8	4.8	3.6		3.5
Ppfia3	5.6	4.7	4.7	3.8	4.0	4.3	4.1	4.0	4.0	2.8	3.5	

Supplemental Fig. 6: Pairwise colocalization of neuropil localized mRNAs analyzed as in Batish et al. (Batish et al., 2012)**.** For each pair of comparisons, the number of overlapping RNA puncta between two channels was divided by the combined count of the two RNAs being compared and expressed as a percentage (average of N=4 mice). Hierarchical clustering of the data revealed a very similar pattern (as shown in Fig 3A) showing that every RNA is colocalized more with highly abundant RNAs (*Camk2a, Ddn, Dlg4*) and show fewer instances of colocalization with RNAs that are of lower abundance (*Pum2, Ppfia3*). RNAs in intermediary clusters also show a similar trend although their specific orders are more variable compared to Fig 3A.

bioRxiv preprint doi: https://doi.org/10.1101/2024.07.13.603387; this version posted May 5, 2025. The copyright holder for this preprint (which was not certified by peer review) is the author/funder, who has granted bioRxiv a license to display the preprint in perpetuity. It is made available under aCC-BY 4.0 International license.

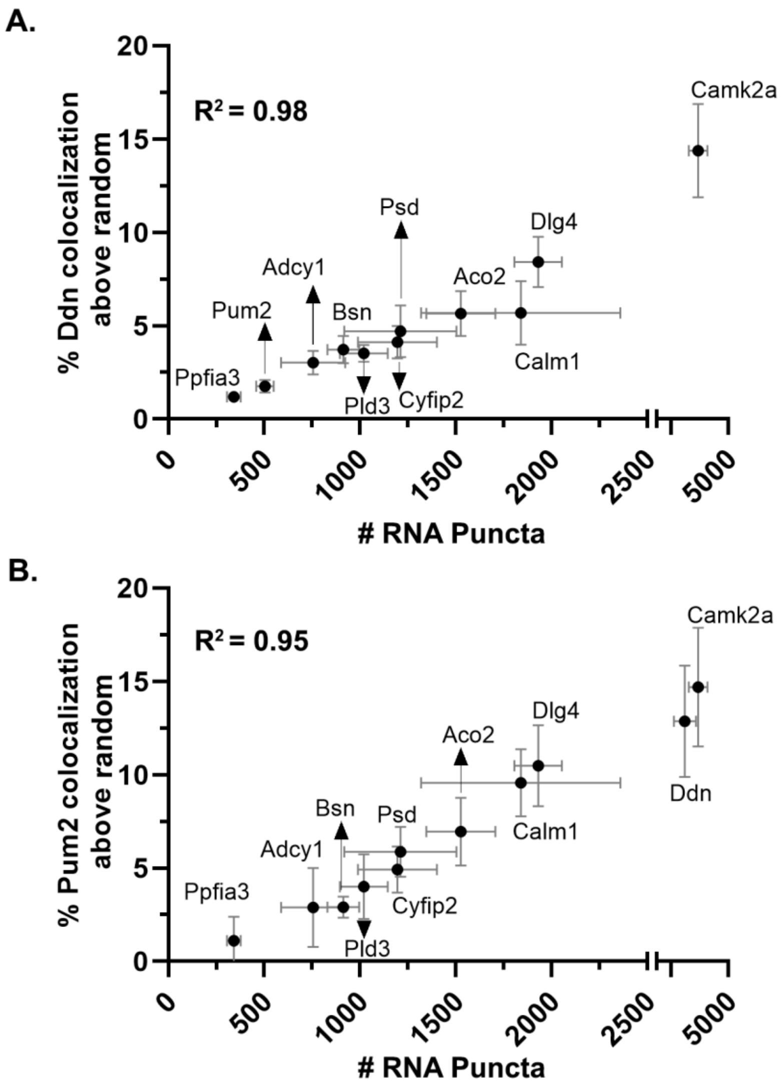

Supplemental Fig. 7 (Refers to Fig. 3C): The positive correlation between pairwise colocalization and mRNA abundance exists regardless of expression. *Ddn* **(A)** and *Pum2* **(B)** exhibit high and low abundance, respectively, in CA2 neuropil. However, these mRNAs display a consistent positive correlation between % colocalization (random colocalization subtracted) and the abundance of the 11 paired mRNAs (*Ddn* R^2 = 0.98 and *Pum2* R^2 = 0.95). (N=4 mice. Error bars indicate SEM.)

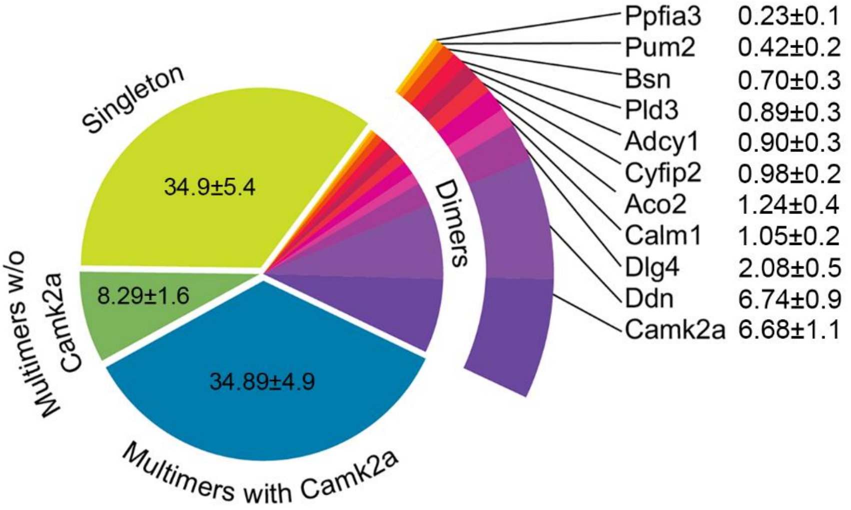

Supplemental Fig. 8 (Refers to Fig. 3D): Pie chart of *Psd* mRNA composition that was observed due to random overlap of mRNA fluorescent puncta. *Psd* image was rotated 90 degrees and colocalization of *Psd* with other eleven mRNAs combined were quantified and averaged from the same four 52X52 μm^2 ROIs per animal as done for the registered experimental images. Individual animal averages were then averaged across N=4 mice. 65.1 ± 5.4% of *Psd* mRNA puncta (vs. 91.86 ± 1.8% in properly registered images) overlap randomly with at least one other mRNA that include dimers (*Psd* with only one other mRNA) or multimers (*Psd* with at least two other mRNAs). Consistent with the pairwise colocalization data where the extent of colocalization scales with mRNA abundance, the percentage of randomly colocalized dimers increases as mRNA abundance increases. However, the percentage of random dimers is equal to or greater than the percentage of dimers from properly registered images, with the exception of *Psd/Camk2a* dimers that are present at lower percentage than experimental (random *Psd/Camk2a* dimers 6.68 ± 1.1% versus properly registered *Psd/Camk2a* dimers 11.58 ± 3.1%). Random *Psd*-multimers with *Camk2a* (34.9 ± 5.0%) and without *Camk2a* (8.29 ± 1.6%) are appreciably lower than experimental images (57.91 ± 5.3% and 12.34 ± 2.9%, respectively), indicating multimer populations dominate colocalized Psd RNA puncta compositions in our data.

bioRxiv preprint doi: https://doi.org/10.1101/2024.07.13.603387; this version posted May 5, 2025. The copyright holder for this preprint (which was not certified by peer review) is the author/funder, who has granted bioRxiv a license to display the preprint in perpetuity. It is made available under aCC-BY 4.0 International license.

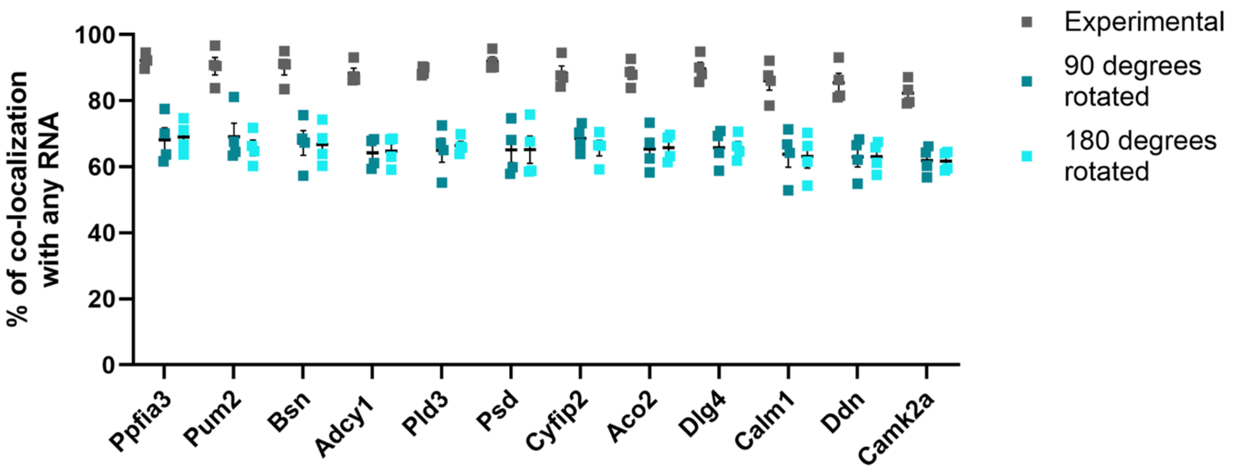

Supplemental Fig. 9 (refers to Fig. 3D): The majority of neuropil localized mRNAs spatially interact with at least one other RNA. Total % colocalization of each mRNA with any of the other 11 mRNAs from properly registered experimental images and 90 degree as well as 180-degree rotated images. Each symbol represents a biological replicate. % colocalization from experimental images were significantly higher compared to that in 90-degree rotated images (unpaired two sample multiple t-test with Welch's correction, $p<0.01$ for every pair) and 180-degree rotated images (unpaired two sample multiple t-test with Welch's correction, $p<0.01$ for every pair). N=4 mice. Error bars indicates SEM.

bioRxiv preprint doi: https://doi.org/10.1101/2024.07.13.603387; this version posted May 5, 2025. The copyright holder for this preprint (which was not certified by peer review) is the author/funder, who has granted bioRxiv a license to display the preprint in perpetuity. It is made available under aCC-BY 4.0 International license.

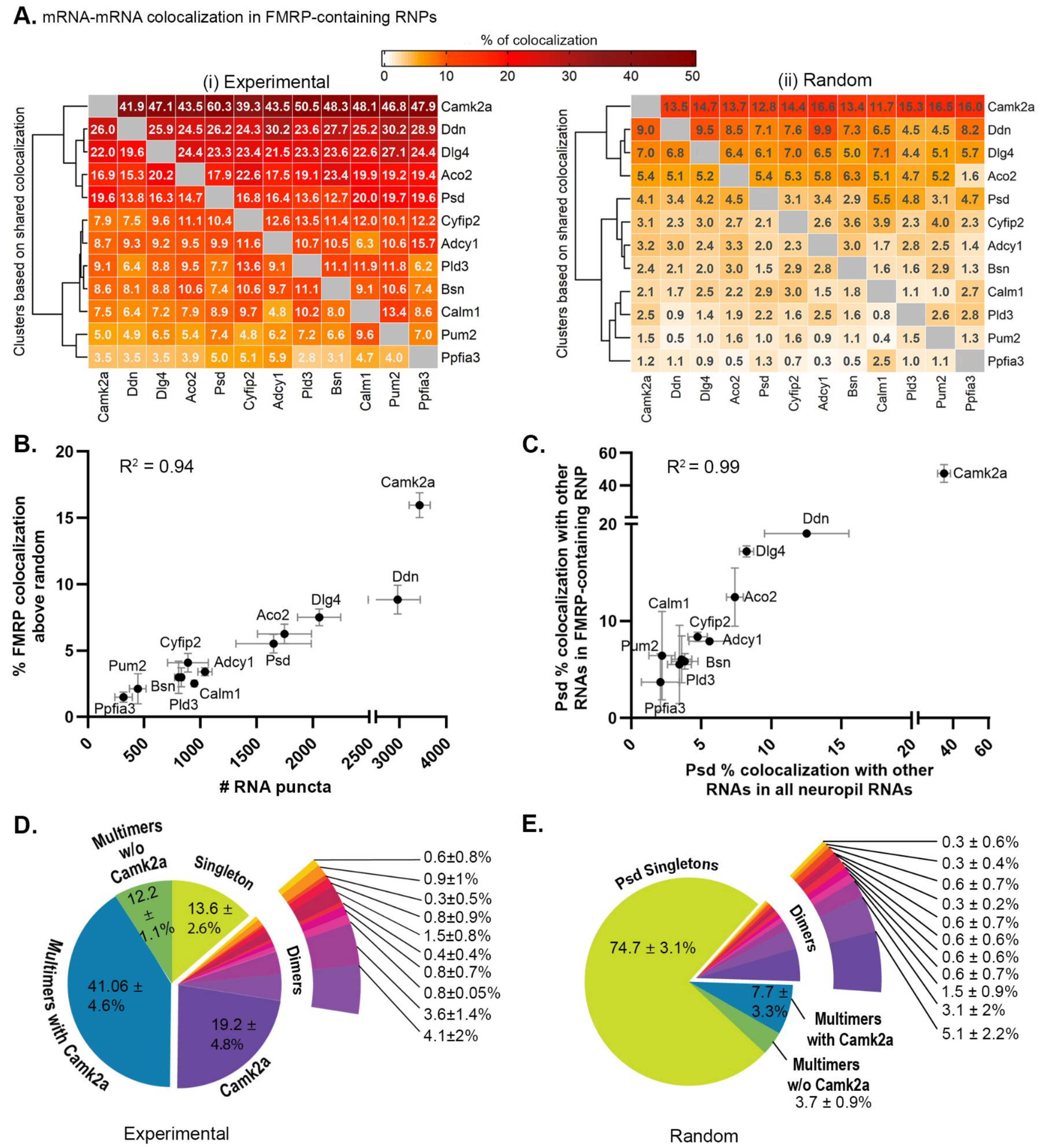

Supplemental Fig. 10 (Refers to Fig. 3): Colocalization of mRNAs within FMRP-containing RNPs.

A. Heatmaps of experimental (Ai. left) and random (Aii. right, 90 degree rotated) % colocalization of each mRNA pair within FMRP containing RNP. Percentage was calculated by dividing the number of overlapping puncta by the total number of the column mRNA puncta.

B. Correlation plot of the percent FMRP colocalized with each mRNA (random colocalization subtracted) and mRNA abundance (R^2 = 0.94).

C. Correlation plot of pairwise *Psd* colocalization percentage with other RNAs within all neuropil RNA puncta (X-axis) and FMRP-containing RNPs (Y-axis).

D. FMRP-containing *Psd* RNP compositions in experimental images.

E. FMRP-containing *Psd* RNP compositions in random 90-degree rotated images. (N=2 mice).

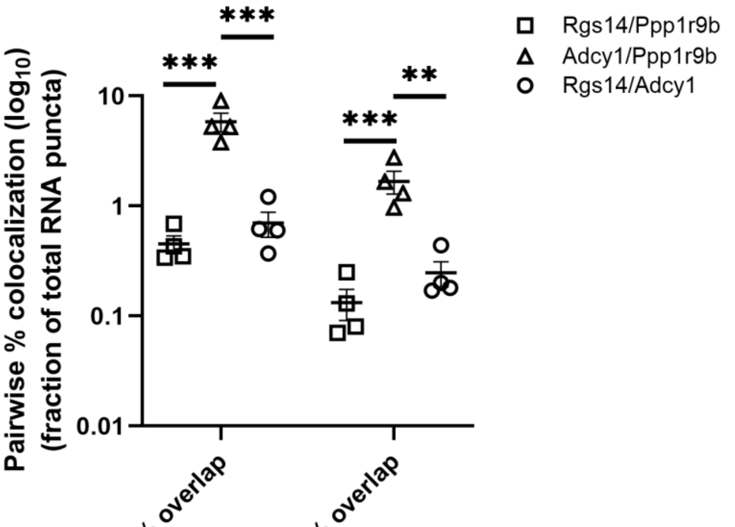

Supplemental Fig. 11 (refers to Fig. 4): Correlation of mRNA pairwise % colocalization with mRNA abundance is consistent across stringent definitions of colocalization. mRNA pairwise colocalization is expressed as a percentage of the combined total mRNA puncta count in DG. Two highly abundant mRNAs *Adcy1* and *Ppp1r9b* are colocalized (5.84 ± 1.13%) significantly higher than *Adcy1/Rgs14* (0.70 ± 0.18%) and *Rgs14/Ppp1r9b* (0.45 ± 0.08%) mRNA pairs when colocalization is defined as >1% overlap (significant effect of mRNA pair, RM ANOVA: $F = 49.26$, $p = 0.0002$, N=4 mice) and this effect is significant for both comparisons

bioRxiv preprint doi: https://doi.org/10.1101/2024.07.13.603387; this version posted May 5, 2025. The copyright holder for this preprint (which was not certified by peer review) is the author/funder, who has granted bioRxiv a license to display the preprint in perpetuity. It is made available under aCC-BY 4.0 International license.

(*Adcy1/Ppp1r9b* vs *Adcy1/Rgs14*: p = 0.0006; *Adcy1/Ppp1r9b* vs *Rgs14/Ppp1r9b*: p = 0.0002, Tukey's post hoc tests). This difference in colocalization between mRNA pairs was recapitulated when colocalization was defined as >50% overlap between channels (significant effect of mRNA pair, RM ANOVA, F = 28.80, p = 0.0008, N=4 mice) and this was also significant for both comparisons (*Adcy1/Ppp1r9b* vs *Adcy1/Rgs14*: p = 0.0040; *Adcy1/Ppp1r9b* vs *Rgs14/Ppp1r9b*: p = 0.0008, Tukey's post hoc tests). Stats were run on the transformed ($\log_{10}$) values as plotted to meet the normality assumption. Tukey's post hoc tests reported on the plot.

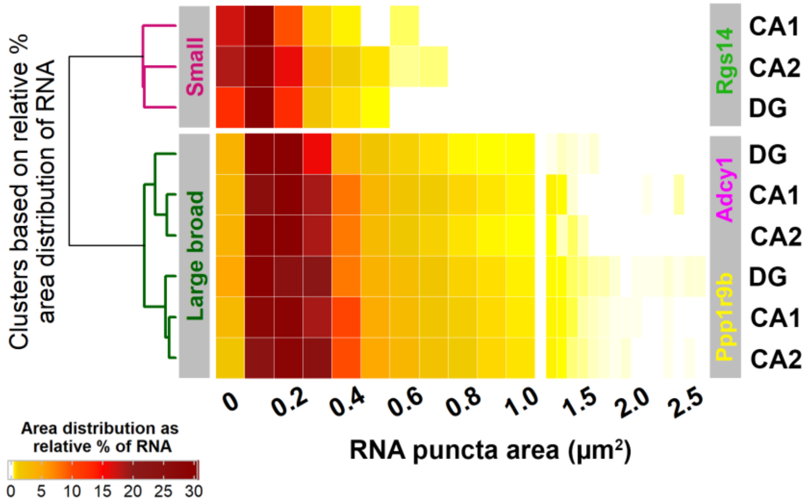

Supplemental Fig. 12 (refers to Fig. 4): *Rgs14*, *Adcy1* and *Ppp1r9b* are variable in fluorescent puncta areas across mRNAs but not cell types. Hierarchical clustering of relative percent area distribution of *Rgs14*, *Adcy1* and *Ppp1r9b* in CA2, CA1 and DG of adult mouse hippocampus. Median puncta areas were not significantly different across cell types for each mRNA although heterogeneity in area distribution across mRNAs was observed similar to the HiPlex data. *Rgs14* median puncta area CA1: 0.10 ± 0.01 μm^2, CA2: 0.10 ± 0.01 um^2, DG: 0.10 ± 0.02 um^2 (no effect of cell-type, RM ANOVA, F = 0.026, p = 0.9744, N=4 mice). *Adcy1* median puncta area CA1: 0.22 ± 0.03 μm^2, CA2: 0.22 ± 0.03 μm^2, DG: 0.19 ± 0.02 μm^2 (no effect of cell type, RM ANOVA: F = 0.5406, p = 0.6083, N=4 mice). *Ppp1r9b* median puncta area CA1: 0.22 ± 0.01 μm^2, CA2: 0.24 ± 0.02 μm^2, DG: 0.22 ± 0.03 μm^2 (no-effect of cell type, RM ANOVA, F = 0.5507, p = 0.6032, N=4 mice). Since *Adcy1* and *Ppp1r9b* median puncta area data were more likely to be a lognormal distribution, we repeated the RM ANOVA on log10 transformed values, which did not change the statistical result.
