# Supplementary material for: Dendritically localized RNAs are packaged as diversely composed Ribonucleoprotein particles with heterogeneous copy number states
Source: bioRxiv. 2024 Jul 17:2024.07.13.603387. Preprint. [Version 1] doi: 10.1101/2024.07.13.603387 (PMC11275876; doi:10.1101/2024.07.13.603387)
Supplement: Supplementary file 1 [file NIHPP2024.07.13.603387V1-supplement-1.pdf]

bioRxiv preprint doi: https://doi.org/10.1101/2024.07.13.603387; this version posted July 17, 2024. The copyright holder for this preprint (which was not certified by peer review) is the author/funder, who has granted bioRxiv a license to display the preprint in perpetuity. It is made available under aCC-BY 4.0 International license.

Supplemental Table 1: Hiplex RNA probe information

Probe	Gene Symbol	NCBI Transcript ID	Ensembl ID	Uniprot	Allen Brain Atlas	Protein Name (Symbol)	Protein Function	Subcellular Localization	FMRP CLIP Rank (Darnell et al., 2011)	CA2 Neuropil Expression (Log2) Farris et al 2019
T1	*Adcy1*	NM_001281768.2	ENSMUSG00000020431	https://www.uniprot.org/uniprotkb/O88444/entry	https://mouse.brain-map.org/gene/show/129123	Adenylate cyclase type 1 (ADCY1)	Adenylate cyclase activity, synaptic transmission, G-protein signalling, Post-syanptic density	Cytoplasm	7	16.44
T2	*Aco2*	NM_080633.2	ENSMUSG00000022477	https://www.uniprot.org/uniprotkb/Q99KI0/entry	https://mouse.brain-map.org/experiment/show?id=67978674	Aconitate hydratase, Aconitase mitochondrial (ACO2)	Essential enzyme in the tricarboxylic acid cycle, isocitrate metabolic processes, mitochondrial metabolism	Cytoplasm, mitochondrial matrix	228	14.47
T3	*Psd*	NM_028627.2	ENSMUSG00000037126	https://www.uniprot.org/uniprotkb/Q5DTT2/entry	https://mouse.brain-map.org/gene/show/49569	Pleckstrin homology and SEC7 domain-containing protein 1 (PSD, also known as Exchange factor for ARF6)	Dendritic spine, postsynaptic density, phospholipid binding	Dendritic spine	297	13.76
T4	*Dlg4*	NM_007864.3	ENSMUSG00000020886	https://www.uniprot.org/uniprotkb/Q62108/entry	https://mouse.brain-map.org/gene/show/13164	Disks large homolog 4 (DLG4, also known as PSD95)	Postsynaptic scaffolding protein, synaptic plasticity, postsynaptic density	Dendritic spine	52	15.25
T5	*Calm1*	NM_001313934.1	ENSMUSG00000001175	https://www.uniprot.org/uniprotkb/P0DP26/entry	https://mouse.brain-map.org/gene/show/12098	Calmodulin-1 (CALM1)	Calcium signal transduction pathway	Cytoplasm	453	16.09
T6	*Bsn*	NM_007567.2	ENSMUSG00000032589	https://www.uniprot.org/uniprotkb/O88737/entry	https://mouse.brain-map.org/gene/show/12003	Bassoon (BSN)	Presynaptic scaffolding protein, protein localization to synapse	Axonal cytoplasm	1	15.4
T7	*Camk2a*	NM_009792.3	ENSMUSG00000024617	https://www.uniprot.org/uniprotkb/P11798/entry	https://mouse.brain-map.org/gene/show/12107	Calcium/calmodulin-dependent protein kinase type II subunit alpha (CAMKIIa)	Calmodulin-binding, synaptic plasticity, dendritic spine development, post-synaptic density	Dendritic Spine	39	18.87
T8	*Pum2*	NM_001160219.1	ENSMUSG00000020594	https://www.uniprot.org/uniprotkb/Q80U58/entry	https://mouse.brain-map.org/experiment/show?id=68845514	Pumilio homolog 2 (PUM2)	Cytosolic RNA-binding protein, translation regulation	Cytoplasm	398	13.35
T9	*Ddn*	NM_001013741.1	ENSMUSG00000059213	https://www.uniprot.org/uniprotkb/Q80TS7/entry	https://mouse.brain-map.org/experiment/show?id=71212512	Dendrin (DDN)	Enables RNA polymerase II cis-regulatory region sequence-specific DNA binding activity	Cytoplasm, dendritic spine membrane	180	16.32
T10	*Pld3*	NM_001317355.2	ENSMUSG00000003363	https://www.uniprot.org/uniprotkb/O35405/entry	https://mouse.brain-map.org/experiment/show?id=77464848	Phospholipase D3 (PLD3)	Lysosomal protein, phospholipase activity,	Lysosomal membrane, endoplasmic reticulum membrane	537	13.31
T11	*Ppfia3*	NM_029741.2	ENSMUSG00000003863	https://www.uniprot.org/uniprotkb/P60469/entry	https://mouse.brain-map.org/experiment/show?id=69202693	Liprin-alpha-3 (PTPRF-interacting protein alpha-3, PPFIA3)	Synaptic vesicle docking, presynaptic active zone cytoplasmic component	Cytoplasm	736	12.26
T12	*Cyfip2*	NM_001252459.1	ENSMUSG00000020340	https://www.uniprot.org/uniprotkb/Q5SQX6/entry	https://mouse.brain-map.org/experiment/show?id=74357791	Cytoplasmic FMR1-interacting protein 2 (CYFIP2)	Actin filament reorganization, neuronal projection development	Cytoplasm	9	15.13

bioRxiv preprint doi: https://doi.org/10.1101/2024.07.13.603387; this version posted July 17, 2024. The copyright holder for this preprint (which was not certified by peer review) is the author/funder, who has granted bioRxiv a license to display the preprint in perpetuity. It is made available under aCC-BY 4.0 International license.

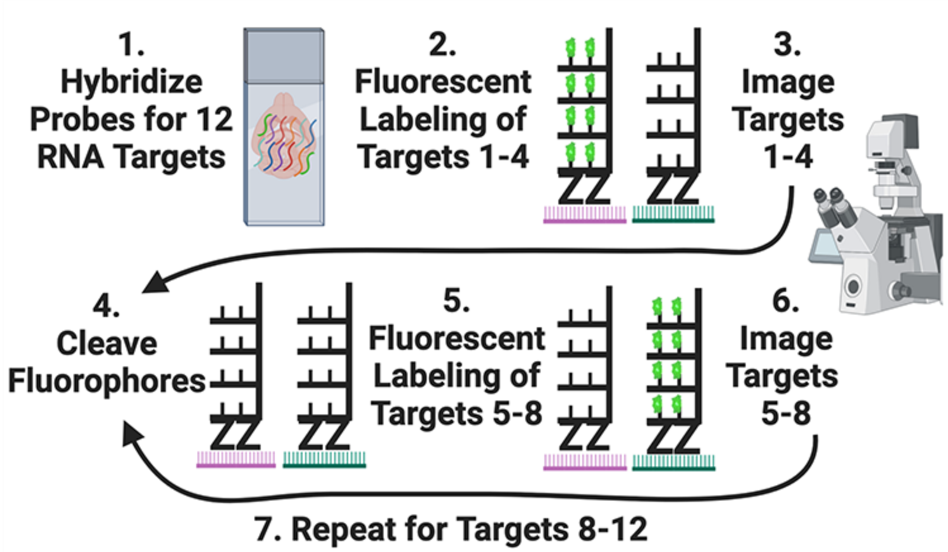

.

Supplemental Fig 1 (Refers to Fig 4AB): Schematic showing workflow of Hiplex smFISH.

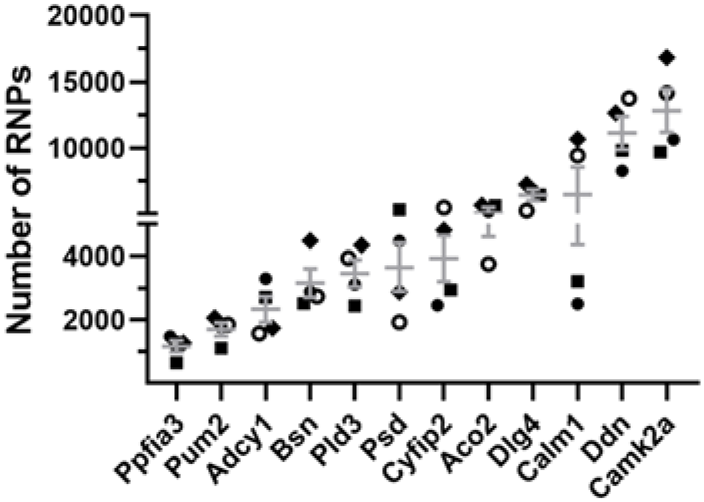

Supplemental Fig 2 (Refers to Fig 4): Abundance of RNAs in CA2 dendrite (N=4 mice). Each symbol represents data from biological replicates. Error bar indicates SEM.

bioRxiv preprint doi: https://doi.org/10.1101/2024.07.13.603387; this version posted July 17, 2024. The copyright holder for this preprint (which was not certified by peer review) is the author/funder, who has granted bioRxiv a license to display the preprint in perpetuity. It is made available under aCC-BY 4.0 International license.

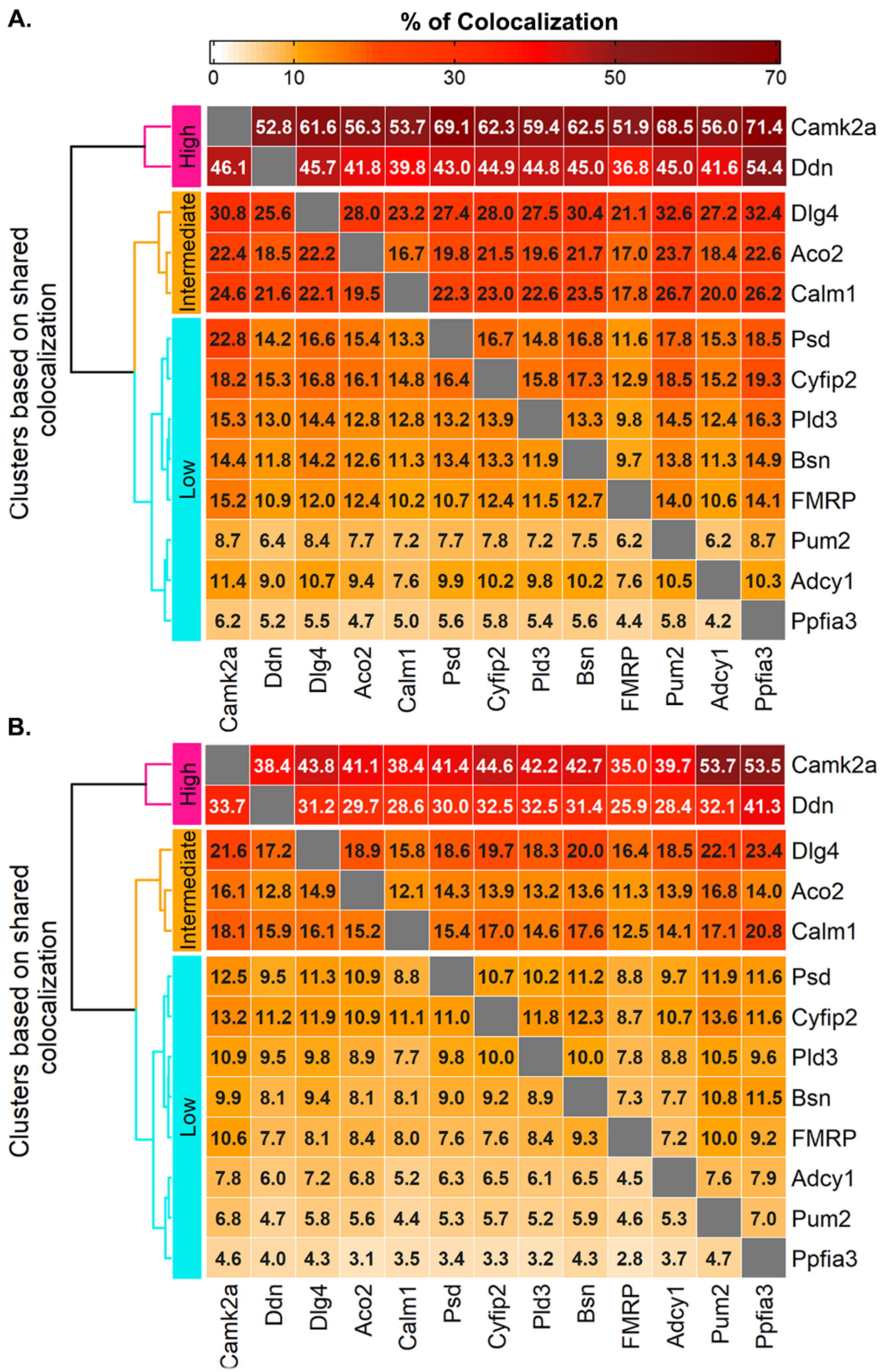

Supplemental Fig 3 (Refers to Fig 5A): Heatmaps showing the total average pairwise colocalization of RNPs in properly registered images **(A)** and in rotated images (chance colocalization) **(B)**. The percentage values in each column are calculated by dividing the number of column RNAs colocalizing with each row RNA by the total number of column RNAs, i.e. 5.2% of Ddn colocalizes with Ppfia3, whereas 54.4% of Ppfia3 colocalizes with Ddn before chance subtraction (see Fig 5A). Values are the average of N=4 mice (four 512 x 512 images quantified per mouse).

bioRxiv preprint doi: https://doi.org/10.1101/2024.07.13.603387; this version posted July 17, 2024. The copyright holder for this preprint (which was not certified by peer review) is the author/funder, who has granted bioRxiv a license to display the preprint in perpetuity. It is made available under aCC-BY 4.0 International license.

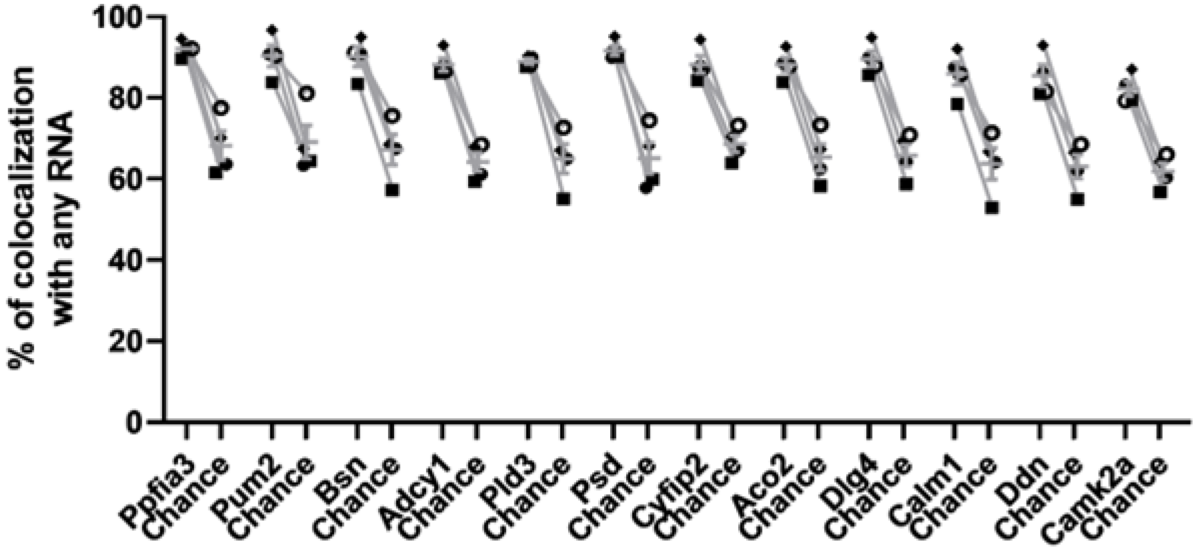

Supplemental Fig 4 (refers to figure 5D): The majority of dendritic RNAs are co-assembled with other RNAs. Total percent colocalization of each RNA with any of the other 11 RNAs. Each symbol represents a biological replicate. Error bar indicates SEM.

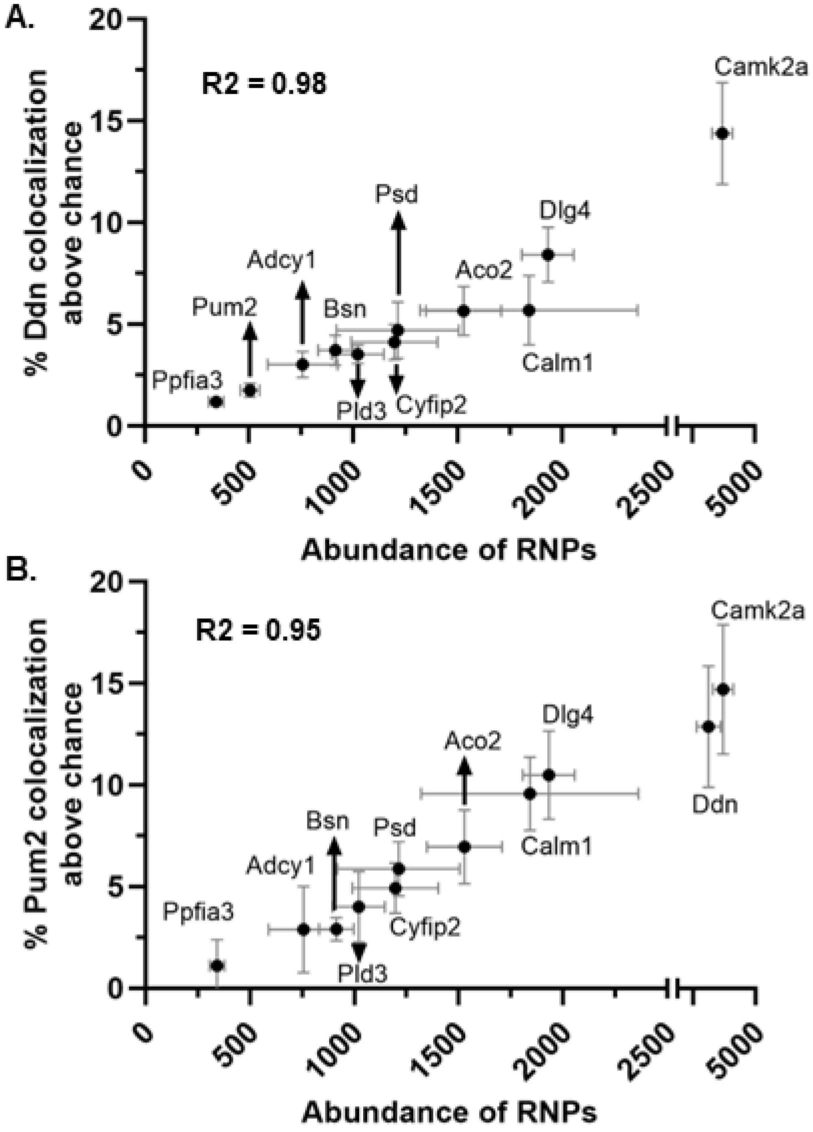

bioRxiv preprint doi: https://doi.org/10.1101/2024.07.13.603387; this version posted July 17, 2024. The copyright holder for this preprint (which was not certified by peer review) is the author/funder, who has granted bioRxiv a license to display the preprint in perpetuity. It is made available under aCC-BY 4.0 International license.

Supplemental Fig 5 (Refers to Fig 5C): The positive correlation between pairwise colocalization and abundance exists regardless of the colocalizer's expression *Ddn* **(A)** and *Pum2* **(B)** exhibit high and low abundance, respectively, in CA2 dendrites. However, these RNAs display a consistent positive correlation between relative percent colocalization (above chance) and the abundance of the 11 paired RNAs (*Ddn* $R^2 = 0.98$ and *Pum2* $R^2 = 0.95$). Error bars indicate SEM.

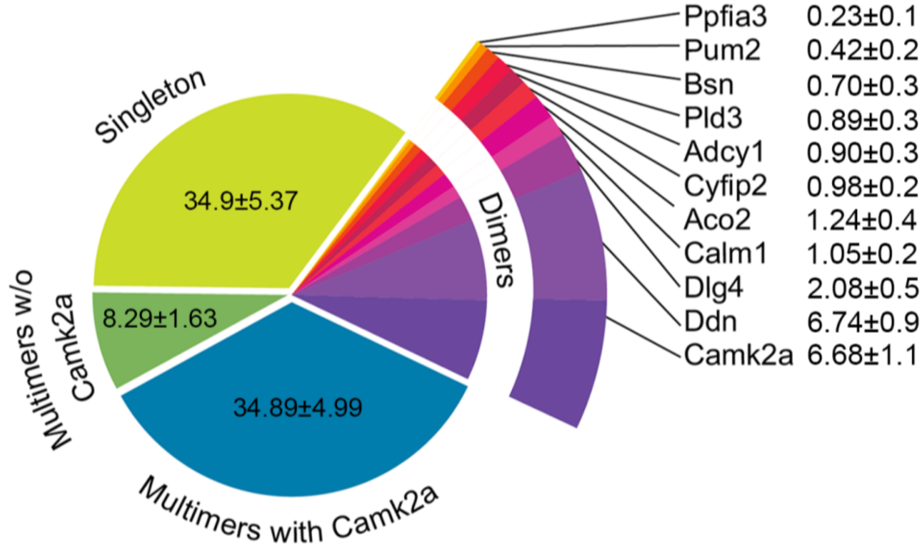

Supplemental Fig 6 (Refers to Fig 5D): Pie chart of *Psd* RNP composition that would be expected due to random overlap of pixels. *Psd* image was rotated 90 degrees and colocalization of *Psd* with each RNA as well as any RNA was quantified from the same four 512X512 ROIs per animal as experimentally registered images then averaged across N=4 mice. 65.1 ± 5.37% of *Psd* RNPs have overlapping pixels from all other RNA channels combined, i.e., colocalized RNAs that include dimers (only one other RNA) or multimers (at least two other RNAs) expected by chance. The percentage of all dimers individually is higher than the percentage from properly registered images except *Psd*/*Camk2a* dimers (6.68 ± 1.1% vs 11.58 ± 3.1%). Consistent with the experimental images, % of dimers due to chance also correlates with the increasing abundance of the RNAs that colocalize with *Psd*. 34.89 ± 4.99% of *Psd* RNPs are multimers that have *Camk2a* (vs 57.91 ± 5.3% experimental) and at least one other RNA colocalized. 8.29 ± 1.63% of *Psd* RNPs are also multimers but do not have *Camk2a*. Lastly, 34.9 ± 5.37% of *Psd* RNPs are not colocalized with any of the other RNAs (vs 8.13 ± 1.8% in experimental images) in our dataset.
